# Integrating Human and Ecosystem Health Through Ecosystem Services Frameworks

**DOI:** 10.1007/s10393-015-1041-4

**Published:** 2015-09-24

**Authors:** Adriana E. S. Ford, Hilary Graham, Piran C. L. White

**Affiliations:** Environment Department, University of York, Heslington, York, YO10 5DD UK; Department of Health Sciences, Seebohm Rowntree Building, University of York, Heslington, York, YO10 5DD UK

**Keywords:** conceptual framework, determinants of health, environmental drivers, public health, socio-ecological systems

## Abstract

The pace and scale of environmental change is undermining the conditions for human health. Yet the environment and human health remain poorly integrated within research, policy and practice. The ecosystem services (ES) approach provides a way of promoting integration via the frameworks used to represent relationships between environment and society in simple visual forms. To assess this potential, we undertook a scoping review of ES frameworks and assessed how each represented seven key dimensions, including ecosystem and human health. Of the 84 ES frameworks identified, the majority did not include human health (62%) or include feedback mechanisms between ecosystems and human health (75%). While ecosystem drivers of human health are included in some ES frameworks, more comprehensive frameworks are required to drive forward research and policy on environmental change and human health.

**Electronic supplementary material:**

The online version of this article (doi:10.1007/s10393-015-1041-4) contains supplementary material, which is available to authorized users.

## Introduction

The Earth’s climate is changing at an unprecedented rate, with rising atmosphere and ocean temperatures, snow and ice retreat, sea-level rise and growing concentrations of greenhouse gases (IPCC 2014). These anthropogenic changes pose serious threats to human health (Myers and Patz 2009; McMichael 2013). Health risks include an increase in thermal stress and injury from floods, storms and bushfires, proliferation of vector-borne disease and dangerous microbes, increased cardiovascular and respiratory disease resulting from air pollution, and a rise in malnutrition and other secondary health effects from the loss of crops, fisheries and livelihoods and displacement (Haines et al. 2006; McMichael et al. 2006; IPCC 2014). Environmental change and its associated health risks are unequally distributed across and within societies; poorer communities in poorer health have contributed least to changes in the Earth’s life-support systems but are and will be disproportionately affected by its adverse effects (McMichael et al. 2008; Woodward and Mcmillan 2015).

The pressing challenge of environmental change is underlining the need for environmental interventions with dual conservation and human health goals (e.g. Bhatia and Wernham 2009; Costello et al. 2009; Harris et al. 2009). Yet the natural environment remains poorly integrated into health research and policy (Lang and Rayner 2012; Reis et al. 2013).

One avenue for promoting this integration is through an ecosystem services (ES) approach (Keune et al. 2013). Increasingly used in environmental research and policy (Fisher et al. 2009), it focuses on the benefits people obtain from ecosystems (MEA 2005a), including health. We focus on ES frameworks to explore how ES approaches represent linkages between the natural (biophysical) environment and human health. Frameworks represent complex processes and pathways in a simplified visual form and can facilitate communication across disciplines and between science and policy (Reis et al. 2013). They can therefore promote shared understandings and joined-up approaches to complex challenges that cross traditional scientific and policy boundaries. Our aim is to examine the opportunities for ES frameworks to provide this bridge, noting frameworks where features relevant to ecosystem and human health are integrated.

In undertaking this analysis, we recognise that ES frameworks serve a broad range of functions and audiences (e.g. stakeholder participation, decision-making or economic evaluation); many are therefore not designed to represent ecosystem and human health. But, taken together, they provide a potential resource for linking environmental and health science and reaching across policy sectors. Some analyses of ES frameworks have been undertaken (Nahlik et al. 2012; Fisher et al. 2013); however, to our knowledge, there has been no broad review of their representation of the links between the natural environment and human health.

To guide our review, we identified the key features that an integrated ecosystem-human health framework should possess. The inclusion of both human and ecosystem health would be essential. In this regard, we define ‘human health’ as “a state of complete physical, mental, and social wellbeing and not merely the absence of disease or infirmity” (WHO 1998). When considering human health, we also include the related term ‘human wellbeing’, which we define as “a multidimensional concept covering physical, psychological, and social aspects of wellness. It includes the presence of positive emotions and moods (e.g. contentment, happiness), and the absence of negative emotions (e.g. depression, anxiety), satisfaction with life, fulfilment, resilience and positive functioning” (drawing on Centers for Disease Control and Prevention 2013). We define ‘ecosystem health’ as “the state of an ecosystem and its associated structure and processes in relation to its ability to function normally, in particular regarding its ability to deliver ecosystem services” (adapted from Rapport et al. 1998b). A healthy ecosystem is therefore one that shows resilience in its structure and function and delivery of ecosystem services in the face of external pressure, and exhibits no obvious signs of distress (see Health of Populations and Ecosystems 2014).

To promote positive states and prevent harm, the determinants of ecosystem and human health—including their interdependency—should also be represented in an integrated ecosystem-human health framework. Time and space are also important, given the temporal and spatial scales over which environmental and social changes have effects (MEA 2005a) and the potential for ecological and human-health tipping points or thresholds, where abrupt system changes occur, or a small change in the driving force results in large health responses (Groffman et al. 2006; McMichael et al. 2006).

## Methods

### Searching and Screening

Scoping reviews are used for emergent areas of research characterised by diversity of approaches. We adopted the methods recommended by Arksey and O’Malley (2005), which include broad search terms and inclusion criteria without quality appraisal filters. During August and September 2014, we searched, with no date restrictions, for papers published in English using search terms relating to ecosystem services frameworks (see Searches 1–6, Table 1). We combined an electronic database search of Web of Science^TM^ (Thomson Reuters 2014) with hand-searching (using citations and searching policy websites). Two broad inclusion criteria were applied.

**Table 1:** Identification Process of Publications Containing Ecosystem Services Frameworks.

	Search terms	No. of additional publications from each search (no. in brackets is the total including repetitions from previous searches)	Excluded publications (total no. excluded/no. which were excluded due to inaccessibility)	Included publications
In title of publication				
Search 1	Ecosystem + services + framework	91 (N/A)	64 (5)	27
Search 2	Ecosystem + services + conceptual	3 (12)	2 (0)	1
Search 3	Ecosystem + services + model	93 (103)	90 (1)	3
In ‘topic’ of publication				
Search 4	‘Ecosystem services framework’	24 (38)	18 (1)	6
Search 5	‘Ecosystem services conceptual framework’	1 (1)	0 (0)	1
Search 6	‘Ecosystem services model’	3 (3)	3 (0)	0
Other publications identified (hand-searched)		N/A	N/A	33
Total no. of publications		215 (248)	177^a^	71

^a^Some publications were excluded because, despite including eligible frameworks, these were republications of pre-existing frameworks rather than new/modified frameworks. Although excluded, these publications were used as a source for indentifying further possible frameworks.

Firstly, the framework should include ES (or closely related terms) or the natural environment, with direct and prominent reference to how the framework links to ES within the text of the publication. Frameworks focusing on a specific habitat (e.g. wetlands), health problem (e.g. infectious diseases), environmental problem (e.g. flooding), or ecosystem service (e.g. food) were only included where they were generic in form. Secondly, the framework should be a visual representation (i.e. a figure/diagram); this included representations of decision-making processes relating to ES and other representations of ES-related problems or scenarios. The full range of socio-ecological frameworks was therefore not included; however, we consider that we identified the majority of frameworks in which ES is a significant feature or improved ES management is a primary objective.

The electronic searches were conducted in succession (Searches 1–6), generating a total of 215 unique potential publications (Table 1). Each publication was assessed against the two inclusion criteria. This resulted in 177 exclusions and 38 inclusions. We also included 33 publications identified through hand-searching. From this total of 71 publications, we identified 84 conceptual frameworks for analysis (some publications contained more than one relevant framework). These are summarised in chronological order in Appendix 1. The frameworks themselves (i.e. in diagrammatic form) are presented in Appendix 2.

### Key Feature Analysis

The frameworks were examined with respect to seven key features: (i) human health; (ii) ecosystem health; (iii) determinants or drivers of human health; (iv) determinants or drivers of ecosystem health; (v) feedback mechanisms (between ecosystem and human health); (vi) time; and (vii) space. All authors were involved in the assessment process which was led by one author with checks on progress and consistency by the other authors. Disagreements were resolved by discussion. The representation of the seven key features in each framework was assessed via its inclusion of relevant terms (e.g. for human health, terms included ‘wellbeing’ ‘welfare’ and ‘health’). While this ensured an objective, transparent and consistent approach, it may have resulted in the scope and potential of frameworks with more oblique reference to these features being inadequately recognised.

For each framework, we provide (Appendix 3) a textual description and a ‘traffic light’ summary of the representation of each feature as follows:

- Red—feature absent or represented in a very limited way;
- Amber—feature partially represented (e.g. related terms or concepts used) and/or present as a minor feature only; and
- Green—feature clearly represented and/or present as a major feature in the framework.

Most traffic-light coding was straight-forward; however, particularly with respect to human and ecosystem health and their determinants, finer judgments were required. We therefore developed guidelines to improve consistency (see Table 2). As with all reviews, a subjective element nonetheless remained. Each framework was assessed and assigned a traffic-light score for each of the seven key features. This was used as a simple way of gauging the level of representation of each of the key features across all of the frameworks.

**Table 2:** Rules for Traffic-Light Coding of Human and Ecosystem Health and Their Determinants.

Traffic-light code	Human health	Ecosystem health	Determinants/drivers of human/ecosystem health
Red	No inclusion of health or well-being, and/or just generic inclusion of social systems etc.	No mention of ecosystems/nature *(not applicable for a review of ES frameworks)* or just generic inclusion of the environment: e.g. nature, ecosystems, or ecosystem services etc. (i.e. no clear reference to ecological processes)	No drivers/determinants shown; (also absence of human/ecosystem health or related concepts automatically means no determinants of that feature is shown)
Amber	Inclusion of human health/well-being/welfare as a minor feature; *and/or* inclusion of related concepts but not directly health e.g. human needs	Inclusion of ecological processes, functioning, structure/components, fluxes etc. as a feature but without suggestion of its condition or complexity of interactions, or further details; *or* inclusion of a breakdown of ecosystem services into types that include intermediate or supporting services (which closely relate to ecological functioning)	Inclusion of generic drivers only without further detail or complexity (e.g. ‘decision-making’, or ‘human actions’, ‘socio-economic drivers’ etc); or (for human health determinants) if only ecosystem-related determinants shown to directly impact on human health/well-being
Green	Inclusion of human health/well-being/welfare as a clear and (fairly) prominent feature	Inclusion of ecological resilience, resistance, integrity, condition, processes, functioning, structure/components, fluxes etc. as a clear and (fairly) prominent feature, particularly if a change in condition is implied and/or details or complexity of interactions are depicted	Inclusion of specific external driving forces with examples or added complexity (e.g. exotic species introductions, changes in land use cover etc.)

## Results: Ecosystem Services Frameworks Analysis

**Figure 1:**
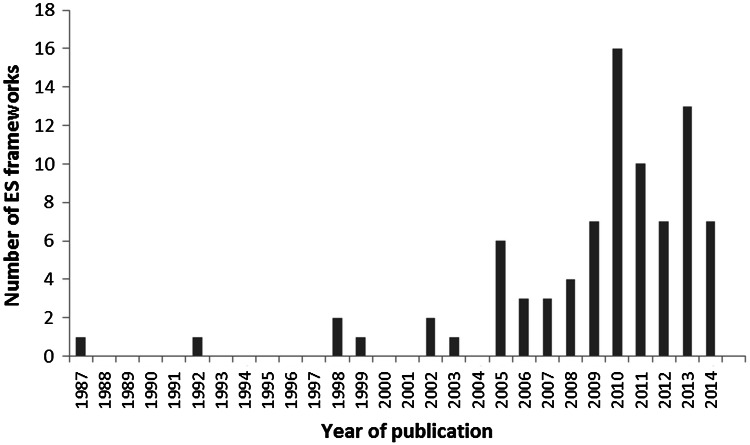
Number of ecosystem services frameworks published from 1987 to 2014.

We identified 84 ES (or ES-related) frameworks, the earliest dating from 1987. The majority (90%) were published from 2005 (see Fig. 1), suggesting that the Millennium Ecosystem Assessment (Millennium Ecosystem Assessment (MEA) 2005a, b) acted as a catalyst for the ES approach (Fisher et al. 2009). Below, we consider the frameworks’ representation of the seven key features.

### Human Health

Most frameworks (62%) do not represent human health (or well-being or welfare) in any way. Of those that do, 23 frameworks (27%) include it as a relatively major feature, whilst nine include it either as a minor feature or via a related concept e.g. human needs. Even those that depict human health prominently, many do so in generic terms e.g. ‘human health risks’ (Rapport et al. 1998a) or ‘health’ as component of ‘human welfare’ (Ekins et al. 2003).

The MEA (2005a) framework was the first to consider different dimensions of human health, including ‘strength’, ‘feeling well’ and ‘access to clean air and water’. The MEA (2005b) framework from the *Health Synthesis* also describes different health impacts of ecosystem change—(i) direct health impacts (e.g. floods, heat waves, water shortage, landslides etc.), (ii) ‘ecosystem-mediated’ health impacts (e.g. altered infectious disease risk, reduced food yields, depletion of natural medicine etc.), and (iii) indirect, deferred and displaced health impacts (e.g. diverse health consequences of livelihood loss, population displacement etc.). We have not found any ES framework published since the MEA that depicts human health more comprehensively, although Balmford et al. (2011) distinguish between ‘physical health’ and ‘psychological well-being’ and Daw et al. (2011) indicate how the human beneficiaries of ES, and hence their well-being, should be disaggregated in ES management.

### Ecosystem Health

Most frameworks (64%) represent ecosystem health in some form, although only 19 (23%) do so clearly. Despite the focus on ES, 30 (36%) have only a generic inclusion of the environment (e.g. ‘ecosystems’, or ‘ecosystem services’). Terms related to ecosystem health in a broad sense are used, such as ecological processes, functioning and structure (e.g. de Groot et al. 2010a, b; Collins et al. 2010; Haines-Young and Potschin 2010 etc.); in some cases, terms more specifically related to ecosystem health are used, such as resilience, integrity, condition and resistance (e.g. MEA 2005a). Cowling et al. (2008) go further with their axis of ‘status of social-ecological system’ depicting a continuum of states from vulnerable to resilient. Rapport et al. (1998a) provide the clearest representation of ecosystem health as ‘changed ecosystem structure and function’, using examples of ‘decreased biodiversity and resilience, increased disease, change in community structure towards r-selected species, eutrophication’, all which suggest negative changes in ecosystem health.

### Determinants and Drivers of Human Health

Most (63%) frameworks have no, or very limited, representation of health determinants or drivers. In the 22 frameworks where human health is relatively prominent, the primary focus is on ES as a determinant of human health, with a strong focus on positive impacts through provisioning, regulating, cultural and supporting services and goods (e.g. UK NEA 2011). More rarely, negative impacts of ecosystems on human health are included (e.g. the MEA *Health Synthesis* framework, 2005b), for example through climate change, stratospheric ozone depletion, forest clearance and land cover change. In a number of frameworks, external driving forces are shown to ultimately drive the ecosystem (thus affecting ES provision and hence human health), but without indication of any additional direct determinants of human health (e.g. de Groot et al. 2010b; Potschin and Haines-Young 2010; Maes et al. 2013 etc.).

Only seven frameworks (8%) represent the determinants of human health in a comprehensive way. The MEA (2005a) conceptual framework illustrates how direct drivers of change (e.g. changes in local land use cover) and indirect drivers of change (e.g. demographic factors) determine human health and well-being both directly and indirectly. Some frameworks also represent drivers of human health beyond those related to ecosystems. For example, Scoones (1998) illustrates how improvement of well-being is determined by livelihood strategies, which are affected by various factors including context, resources and institutional processes. Additionally, White et al. (2010) and Bastian et al. (2013), although both representing ES as a determinant of human health, also include the role of social factors in ES delivery, including legislation, incentives, technological development, governance and equity (White et al. 2010) and the role of stakeholders and valuation (Bastian et al. 2013). Reis et al. (2013) explicitly refer to ‘determinants of health’ in their framework, drawing upon the ‘social determinants of health’ (SDH) model (Dahlgren and Whitehead 1991), a model that has underpinned global, national and local strategies on public health and health inequalities (WHO 2008).

### Determinants and Drivers of Ecosystem Health

Most frameworks (68%) include some determinants or drivers of ecosystem health (or ecosystems more generally), with 13 (15%) depicting them strongly. The earliest example is Rapport et al. (1998a) who include ‘human pressures on ecosystems and landscapes’, such as harvesting, waste residuals, physical restructuring, magnified extreme events and exotic species introductions. More recent frameworks, including the MEA (2005a) conceptual framework and de Groot et al. (2010b), also include direct and indirect drivers of change upon ecosystems and ES delivery. Human drivers are often represented as having negative impacts on ecosystems (e.g. Rapport et al. 1998a, b; de Groot et al. 2010b; White et al. 2010) or a negative impact is implied, such as ‘pressures’ (e.g. Haines-Young and Potschin 2010).

There are two other noteworthy approaches to conceptualising ecosystem health drivers. Firstly, there is the Framework for Ecosystem Service Provision (FESP), based on a Driver-Pressure-State-Impact-Response (DPSIR) framework (Rounsevell et al. 2010). It depicts ‘states’ (which include the supporting system) determined by ‘pressures’ (e.g. climate change, land use change, invasive species, air pollution), which are determined by ‘drivers’ (e.g. economy, demography, society, technology) and ‘responses’ (policy, strategic decisions and management). A similar approach is taken by Kelble et al. 2013. Secondly, there is the Press-Pulse Dynamics framework (Collins et al. 2010). This shows the ecological system determined by pulse events (sudden events e.g. fire, drought, storms, dust events etc.) and press events (extensive, pervasive, and subtle changes e.g. climate change, nutrient loading, sea-level rise etc.). These events are also shown to be influenced by external drivers such as climate and globalisation, and human behaviour and outcomes.

### Feedback Mechanisms (Between Human Health and Ecosystem Health)

With human health absent from most frameworks, feedback mechanisms between human and ecosystem health are largely absent too. Where human health is present, most only show a one-directional pathway from ecosystem health to human health outcomes, e.g. the MEA (2005a) framework on harmful effects of ecosystem change on human health. Only 25% (21) of the frameworks include feedback mechanisms, for example the overall MEA (2005a) framework, which includes the impact of human well-being directly on ecosystems and indirectly on ecosystem drivers, thus closing the loop.

A few frameworks go further and identify the mechanisms involved. For example, de Groot et al. (2010b) include governance and decision-making as mechanisms which impact upon the determinants of ecosystems and biodiversity, and similarly Duraiappah et al. (2014) link natural systems and human well-being via institutions and governance. Bastian et al. (2013) include the mechanisms ‘use, management, decision, participation, steering’ to close the loop between ecosystem benefits (which include well-being), and ecosystem properties. White et al. (2010) illustrate how legislation, incentives and voluntary agreements influence the outputs (e.g. waste and pollution) from social development and well-being, which then impact upon the ecosystem either directly or indirectly. Some frameworks depict decision-making and governance processes in further detail, e.g. Lopes and Videira (2013) describe the feedback mechanism between policy formation and assessment with decision-making and implementation, in the context of ES. However these processes relate to feedback mechanisms solely within ES management, rather than describing the feedback mechanisms between human health and ecosystem health.

### Time

Time is not represented in the majority (82%) of the frameworks. Eleven have time as a minor feature, and only four frameworks (5%) have a prominent representation of time. The earliest example of the inclusion of time is the MEA (2005a), although not as a significant feature.

Among the frameworks where time is strongly represented, Chapin et al. (2006) distinguish between ‘slow variables’ (relatively constant over years to decades) and ‘fast variables’ (change on daily, seasonal and inter-annual timescales) as key features. Wallace (2007) represents time as a change between two time-points, for example changes following ES management. The framework by Bastian et al. (2013) also includes the ‘temporal aspect’; ‘time scale, driving forces, changes and scenarios’ links to the five pillars of their system—although, like the framework of Wallace (2007), this does not necessarily capture longer timeframes. The framework developed by Reed et al. (2013) includes a longer time perspective by showing the impact on the natural and social system of ‘future change’ in comparison to ‘current context’. However overall, ES frameworks pay relatively little attention to ecosystem-human health inter-relationships over time.

### Space

The spatial dimension is absent from 69% of the frameworks. While only 12 frameworks (14%) include it as a prominent feature, they do so in innovative ways. The framework by Cowling et al. (2008) has a spatial axis from regional to local which corresponds to different stages for safeguarding ES and Bastian et al. (2012) depict space as a continuum from local/micro through to global/macro- space, with examples of different types of natural or constructed space for each spatial level. López-Hoffman et al. (2010) take a different approach, illustrating the relationship between two world regions, linked by transboundary interventions. Both Fisher et al. (2009) and Serna-Chavez et al. (2014) depict the spatial relationships between ES production areas and service benefit areas.

While the focus on space can mean other dimensions are given less attention, some frameworks include space within an integrated and comprehensive approach, e.g. Robinson et al. (2009) and Carpenter et al. (2009) divide their framework into spatial levels. Possibly the best example is the framework by Duraiappah et al. (2014). Here, space is depicted in three ways, relating to (i) society (household, community, nation, region, globe); (ii) institutions and governance (community, national, regional, international) and (iii) ecosystems (resource system, landscape, bioregion, biome, planet). At these different scales, the natural and social systems are linked via institutions and governance, the productive base (capital) and ES. As this suggests, space has been more extensively incorporated into ES frameworks than time.

### Highly Rated Frameworks

The traffic-light system enabled us to identify frameworks that gave prominence to ecosystem and human health and included most of the other five features well or moderately well. Three frameworks stood out using this scoring system. These were (i) the Press–Pulse Dynamics Framework by Collins et al. (2010); (ii) the conceptual framework of The Economics of Ecosystems and Biodiversity (TEEB) by de Groot et al. (2010b), which builds on the MEA main conceptual framework and the cascade model by Haines-Young and Potschin (2010); and (iii) the efficiency framework for an ES approach to sustainability by White et al. (2010) (see Table 3).

**Table 3:** The Three Top-Scoring Frameworks Based on Overall Scores for all Seven Features (Excluding Those Not Scoring ‘Green’ for Ecosystem Health and Human Health).

Publication	Title/brief description	Purpose	Summary of main components
Collins et al. (2010)	Press–pulse dynamics framework	To build trans-disciplinary knowledge of social–ecological systems and contribute to the development and testing of theory within these disciplines.	Consists of a social template (human behaviour and human outcomes) and a biophysical template (community structure and ecosystem function), linked together through ecosystem services and by pulse and press events (‘press’ referring to extensive, pervasive, and subtle change, and ‘pulse’ referring to sudden events). The systems are influenced by external drivers such as climate and globalisation
de Groot et al. (2010b)	Conceptual framework for linking ecosystems and human well-being	Conceptual framework of The Economics of Ecosystems and Biodiversity (TEEB) to provide a basis for the TEEB report, in relation to linking ecosystems with human well-being	Consists of the interlinking components of: ecosystems and biodiversity; services; human well-being; governance and decision-making; and direct/indirect drivers. Based on the MEA (2005a) overall conceptual framework
White et al. (2010)	Efficiency framework for an ecosystem services approach to sustainability	To provide a conceptual basis for assessing the components of social-ecological systems and the links between them, based around magnitudes and efficiencies of conversion between states	Consists of three broad sub-systems: ecosystem functions, ecosystem services, and social development and well-being. These systems interact with each other (e.g. through impacts, consumption, and trade-offs), representing a transfer of state (e.g. from ecosystem functions to ecosystem services). Within each sub-system feedback loops and mechanisms (e.g. governance, incentives etc.) are depicted

A number of other frameworks also provide examples of how features relevant to ecosystem and human health can be integrated. These include the following:

- Ekins et al. (2003), showing environmental functions and attributes in relation to human influences and welfare;
- Rapport et al. (1998a), showing the linkages between pressures from human activity, ecosystem change and degradation of ecosystem and human health;
- The main MEA (2005a) conceptual framework, showing the relationship between indirect and direct drivers of change with ES and human well-being/poverty alleviation. However, this framework, along with that by López-Hoffman et al. (2010) which also scored highly overall (based on the MEA overall framework and depicting transboundary ES), only scored moderately for their representation of ecosystem health;
- The MEA (2005a) framework depicting biodiversity as response variable affected by global change drivers;
- Maes et al. (2013)—the conceptual framework for EU-wide ecosystem assessments, which emphasises the role of biodiversity in delivering ES and human well-being; and
- Bastian et al. (2013)—the extended Ecosystem Properties, Potentials, and Services (EPPS) framework.

## Conclusions

Today’s economic systems, together with the lifestyles that they sustain, are driving environmental change and its associated impacts on ecosystem and human health. To address the interacting processes linking ecosystem and human health, new ways of working across science and policy are urgently required. This in turn demands ways of working across communities—for example, across disciplines and across local, national and global policy—which, until recently, have developed and operated separately (Myers and Patz 2009; Keune et al. 2013; Woodward and Mcmillan 2015). Frameworks are recognised as vehicles through which knowledge and perspectives can be shared across boundaries; they can help open up the common ground necessary for cooperation around shared goals (Star and Griesemer 1989). ES frameworks have particular potential in this respect.

The relationship between ecosystems and human well-being underpins the ES approach, an approach increasingly used within environmental science and policy (Gómez-Baggethun et al. 2010). ES frameworks therefore provide an exciting avenue for advancing dual conservation and human health outcomes. The 84 ES frameworks identified in our review had varied objectives; nonetheless, they provide a basis on which to build an integrated ecosystem and human health framework capable of informing environmental interventions with dual conservation and human health goals.

To promote this integrated perspective, we make the following suggestions. With respect to human health, we recommend building upon the MEA (2005b) approach of ‘direct’, ‘ecosystem-mediated’, and ‘indirect, deferred and displaced’ health impacts, which could be illustrated using specific health conditions linked to specific changes in ES delivery. In this context, it is also important to consider that biodiversity’s impact on human health, for example in relation to infectious disease, is not always beneficial, and may be considered as a ‘disservice’ rather than a ‘service’ (Dunn 2010). Recognising such plurality of concepts is important in building bridges across disciplines (Keune et al. 2013) and frameworks that seek to integrate ecosystems and public health should be flexible to accommodate these negative impacts of ecosystems, as well as positive ones. For ecosystem health, we recommend approaches that, like Rapport et al. (1998a, b), indicate what constitutes poor versus good ecosystem health. This would support the use of standard measures of ecosystem health and aid interpretation by the public health community for which measurement and monitoring are well established tools supporting policy-making and impact assessment.

With respect to health determinants, more attention needs to be given to non-environmental determinants and their effects on both ecosystem and human health. Potential resources here are public health frameworks with the social determinants of health (e.g. Dahlgren and Whitehead 1991; WHO 2008) and the wider ecosystem (e.g. McLeroy et al. 1988; Lang and Rayner 2012) as their primary focus. For ecosystem drivers, the press-pulse approach (Collins et al. 2010) would complement the representation of human health in MEA (2005b). Pulse events are likely to have substantial direct health impacts, whilst press events are likely to have significant indirect, deferred and displaced health impacts. Both press and pulse events have the potential to have substantial ecosystem-mediated health impacts. This could be combined with FESP, in order to incorporate pressures, drivers, and responses more effectively.

We suggest incorporating feedback mechanisms between ecosystem and human health (e.g. White et al. 2010; Bastian et al. 2013). These are essential for policy guidance on how to promote ecosystem and human health co-benefits by enhancing positive feedbacks and minimising negative feedbacks.

Space figures centrally in a number of ES frameworks, and the use of spatial levels (e.g. local, national, global) would align well with resource allocation and planning in the environmental and public health communities. Time needs to be equally prominent, and represented at a scale extending beyond the short-term. This is particularly important for capturing longer-term ecosystem and human health benefits of ecosystem interventions and for risk assessments of potential thresholds or tipping points in ecosystem and human health (Lenton 2011).

While our review points the way to a new framework incorporating these features, its development would require detailed work and extensive consultation. A parallel review of how ecosystem processes are represented in public health frameworks would also be needed to ensure any new framework spoke to both the environmental and the health sector. What our review demonstrates however is the scope for ES approaches to invigorate and inform cross-sector working, alerting researchers and decision-makers to co-dependencies between ecosystem and human health and highlighting the opportunities for policies that can benefit ecosystems and the health of humans who depend upon them.

## Electronic supplementary material

Supplementary material 1 (PDF 291 kb)

Supplementary material 2 (PDF 7,275 kb)

Supplementary material 3 (PDF 141 kb)
