# Supplementary material for: Integrating Human and Ecosystem Health Through Ecosystem Services Frameworks
Source: Ecohealth. 2015 Sep 24;12:660–71. doi: 10.1007/s10393-015-1041-4 (PMC4700085; doi:10.1007/s10393-015-1041-4)
Supplement: Supplementary file 1 — Supplementary material 1 (PDF 291 kb) [file 10393_2015_1041_MOESM1_ESM.pdf]

Appendix 1: Summary of ecosystem services frameworks

Author(s)	Figure number (see A2)	Full reference	Title/ brief description	Purpose	Summary of main components
De Groot 1987	A2.1	de Groot, R.S. (1987) Environmental functions as a unifying concept for ecology and economics. *The Environmentalist*, 7:105-109	The relationship between natural processes and human needs	To highlight the importance of nature conservation for human well-being.	Links natural processes and components with human needs and activities through a two-way functional process, which emphasises their respective influence on each other.
Costanza & Daly 1992	A2.2	Costanza, R. & Daly, H.E. (1992) Natural capital and sustainable development. *Conservation Biology*, 6: 37-46	Types of natural and human-made capital stocks, good and service flows, and their interdependence	To encourage maintenance of a total natural capital stock at or above the current level, which is needed to achieve 'strong sustainability'.	Illustrates the interdependence between natural capital (non-renewable and renewable), human capital and manufactured capital, with economic and ecosystem goods and service flows.
Rapport et al 1998	A2.3	Rapport, D.J., Costanza, R. & McMichael, A.J. (1998) Assessing ecosystem health. *Trends in Ecology and Evolution*, 13:397-402	Linkages between pressures from human activity, ecosystem change and degradation of ecosystem and human health	To encourage more active collaboration between the ecological, social and health sciences.	The framework consists of five components: human pressures on ecosystems and landscapes, changed ecosystem structure and function, decreased ES, human health risks, and human society's responses.
Scoones 1998	A2.4	Scoones, I. (1998). Sustainable rural livelihoods: a framework for analysis. *IDS Working Paper,* 72: 1–22	Sustainable livelihoods framework: a framework for analysis	To support the analysis and operationalization of the sustainable livelihoods approach.	Consists of several key features for livelihoods analysis: context, livelihood resources, institutional processes, livelihood strategies, and livelihood outcomes.
Leach et al 1999	A2.5	Leach, M., Mearns, R. & Scoones, I. (1999) Environmental entitlements: dynamics and institutions in community-based natural resource management. *World Development*, 2: 225-247	Environment entitlements framework	To highlight the central role of institutions and social actors in mediating environment-society relationships and commanding ES.	Links differentiated social actors (and their capabilities) with environmental goods and services, through processes of endowment and entitlement mapping. This is influenced by institutions at different spatial scales.
Perrings et al 2002	A2.6	Perrings, C., Folke, C. & Mäler, K.G. (2002) The ecology and economics of biodiversity loss: the research agenda. *Ambio,* 21: 201-211	The economy as a part of its life-supporting environment	To encourage maintenance of biodiversity at a level that ensures ecosystem resilience, in order to provide for human consumption and existence. Also to stress the importance of an interdisciplinary approach to biodiversity.	The economic system sits within the ecological system, and receives ecosystem services, natural resources and energy from that ecological system. The economic system expels degraded energy, resources and pollution.

De Groot et al 2002	A2.7	de Groot, R.S., Wilson, M.A. & Boumans, R.M.J. (2002) A typology for the classification, description and valuation of ecosystem functions, goods and services. *Ecological Economics*, 41: 393–408	Framework for integrated assessment and valuation of ecosystem functions, goods and services	To provide a standardised ecosystem services framework in order to improve assessment of goods and services.	Illustrates the relationship between ecosystem structure and process, ecosystem functions, ecosystem goods and services, different values to society, and decision-making processes.
Ekins et al 2003	A2.8	Ekins, P. Simon, S., Deutsch, L., Folke, C. & de Groot, R. (2003) A framework for the practical application of the concepts of critical natural capital and strong sustainability. *Ecological Economics*, 44: 165-185	Environmental functions and attributes: human influences and welfare	To help show how sustainability standards can be derived, and to enable policy makers to determine and reduce the 'sustainability gap' and thus move towards environmental sustainability.	Illustrates the relationship between influences (e.g. social, economic etc.) and natural capital (both its elements and functions), with 'functions for people', which in turn affects human welfare.
Millennium Ecosystem Assessment 2005a	A2.9	MEA (2005a) *Ecosystems and human well-being: current state and trends, Volume 1*. Island Press: Washington DC. Available at: http://www.unep.org/maweb/en/Condition.aspx [Accessed 22 July 2014]	Linkages between ecosystem services and human well-being	To demonstrate the benefits that people derive from nature.	ES are categorised into provisioning, regulating, cultural and supporting services, and human well-being is comprised of security, basic material for a good life, health, good social relations, and freedom of choice and action.
Millennium Ecosystem Assessment 2005a	A2.10	MEA (2005a) *Ecosystems and human well-being: current state and trends, Volume 1*. Island Press: Washington DC. Available at: http://www.unep.org/maweb/en/Condition.aspx [Accessed 22 July 2014]	Millennium Ecosystem Assessment Conceptual Framework	To provide a theoretical basis for the MEA, governments, the private sector and civil society to assess and factor in options to enhance the contribution of ecosystems to human well-being.	Shows the relationship between indirect drivers of change (such as population, technology and lifestyle) and direct drivers of change (such as changes in local land use and cover) with ES and human well-being/poverty alleviation.
Millennium Ecosystem Assessment 2005a	A2.11	MEA (2005a) *Ecosystems and human well-being: current state and trends, Volume 1*. Island Press: Washington DC. Available at: http://www.unep.org/maweb/en/Condition.aspx [Accessed 22 July 2014]	Different stages of assessment for linking ecosystem condition with human well-being	To support the implementation of the MEA (2005) overall conceptual framework.	Highlights the key stages of assessment for linking ecosystem condition, ES and human well-being, including assessing ecosystem condition and trends, value of ES for human well-being, and evaluation of trade-offs in ES.
Millennium Ecosystem Assessment 2005a	A2.12	MEA (2005a) *Ecosystems and human well-being: current state and trends, Volume 1.* Island Press: Washington DC. Available at: http://www.unep.org/maweb/en/Condition.aspx [Accessed 22 July 2014]	Biodiversity as response variable affected by global change drivers and as factor modifying ecosystem processes and services and human well-being	To illustrate the role of biodiversity in ES and provide basis for MEA (2005) analysis.	Consists of six interlinking components: biodiversity, functional traits, ecosystem processes, ecosystem services, human well-being, and global changes.

Millennium Ecosystem Assessment 2005b	A2.13	MEA (2005b) *Ecosystems and Human Well-being: Health Synthesis.* Island Press: Washington DC. Available at: http://www.millenniumassessment.org/documents/document.357.aspx.pdf [Accessed 11 March 2014]	Harmful effects of ecosystem change on human health	Framework of the MA Health Synthesis to illustrate why ecosystems are important to human health.	Describes the causal pathway from escalating human pressures on the environment through to ecosystem changes resulting in diverse health consequences, including 'direct', 'ecosystem-mediated' and 'indirect, deferred and displaced' health impacts.
National Research Council 2005	A2.14	National Research Council (2005) *Valuing Ecosystem Services: Toward Better Environmental Decision Making.* National Academies Press: Washington, D.C., USA	Connections between ecosystem structure and function, services, policies, and values.	To summarise current state of knowledge regarding the links between ecosystems and ES values.	Consists of ecosystem structure and function leading to ecosystem goods and services, which in turn leads to different values. This results in human actions that affect the ecosystem.
Hein et al 2006	A2.15	Hein, L., van Koppen, K., de Groot, R.S. & van Ierland, E.C. (2006) Spatial scales, stakeholders and the valuation of ecosystem services. *Ecological Economics,* 57: 209– 228	The ecosystem valuation framework	To aid the valuation of ES, including a procedure to avoid double-counting of regulating services.	Describes four valuation steps: (i) specification of ecosystem boundaries, (ii) assessment of the ES supplied, (iii) valuation, and (iv) aggregation/ comparison of the values.
Chapin et al 2006	A2.16	Chapin, F.S., Lovecraft, A.L., Zavaleta, E.S., Nelson, J., Robards, M.D., Kofinas, G.P. et al. (2006) Policy strategies to address sustainability of Alaskan boreal forests in response to a directionally changing climate. *Proceedings of the National Academy of Sciences of the United States of America,* 103:16637-16643	Diagram of a social–ecological system	To facilitate understanding of the linkages between global-scale changes and local-scale dynamics of human–environment interactions, in order to develop sustainability policy strategies.	Consists of an ecological and a social subsystem, and range of variables at different temporal and spatial scales. Human actors are affected by environmental and social impacts as well as ES, and these human actors also affect the social-ecological system through a variety of institutions.
Chapin et al 2006	A2.17	Chapin, F.S., Lovecraft, A.L., Zavaleta, E.S., Nelson, J., Robards, M.D., Kofinas, G.P. et al. (2006) Policy strategies to address sustainability of Alaskan boreal forests in response to a directionally changing climate. *Proceedings of the National Academy of Sciences of the United States of America,* 103:16637-16643	Ecological institutions that influence ecosystem services	To highlight how different types of institutions differ in their ecological goals and consequences, which needs to be considered to develop sustainability policy strategies.	Consists of an ecological and a social subsystem, which affect 'human actors', which in turn affect the subsystems through four types of institution (resource-harvest, hazard-reduction, resource-conservation and ecological-externality producing).

Brauman et al 2007	A2.18	Brauman, K.A. Daily, G.C., Duarte, T.K. & Mooney, H.A. (2007). The nature and value of ecosystem services: an overview highlighting hydrologic services. *Annual Review of Environment and Resources,* 32:67–98	Policy-relevant questions for understanding, assessing, and managing ecosystem services	To aid the operationalization of the ES approach as a basis for policy decisions.	Four key interconnecting themes of policy questions are depicted: the biophysical generation of ES; beneficiaries and producers; valuation; and policy.
Wallace 2007	A2.19	Wallace, K.J. (2007) Classification of ecosystem services: Problems and solutions. *Biological Conservation*, 139: 235-246	Description of the relationships among ecosystem elements and processes	To support decisions in natural resource management	Depicts the relationship between structure and composition of ecosystems (e.g. biodiversity, air etc.), with ecosystem processes (e.g. water cycle). It includes a temporal element where ecosystem processes redistribute matter and energy amongst assets, or evolve new assets, between two points in time.
Wallace 2007	A2.20	Wallace, K.J. (2007) Classification of ecosystem services: Problems and solutions. *Biological Conservation,* 139: 235-247	Simplified scheme of the ecosystem pathways for delivering five ecosystem services	To illustrate how a manager may construct a diagram of ecosystem pathways when consider management options/ needs.	The ecosystem pathway starts with abiotic and biotic assets and processes, through to the delivery of ES. It is demonstrated with a specified set of five services and includes processes such as surface water flow and water treatment.
Cowling et al 2008	A2.21	Cowling, R.M., Egoh, B., Knight, A.T., O'Farrell, P.J., Reyers, B., Rouget, M. et al (2008) An operational model for mainstreaming ecosystem services for implementation. *Proceedings of the National Academy of Sciences of the United States of America*, 105: 9483-9488	An operational model for implementing the safeguarding of ecosystem services	To help stakeholders to implement effective on-the-ground management that will lead to ecological resilience and safeguarding of ES.	Consists of three project phases (assessment, planning and management) in relation to spatial scale, status of the socio-ecological system and level of stakeholder collaboration. Three types of assessment are depicted – social, biophysical and valuation.
Fisher et al 2008	A2.22	Fisher, B., Turner, K., Zylstra, M., Brouwer,R., de Groot, R., Farber, S. et al. (2008) Ecosystem services and economic theory: integration for policy-relevant research. *Ecological Applications,* 18(8): 2050–2067	Stylized relationships among representative intermediate services, final services, and benefits	To provide an operational, meaningful and consistent definition of ES.	Shows the relationship between intermediate ES (such as soil formation, primary production etc.), with final services (such as clean water provision and food production), which in turn lead to 'benefits' (such as drinking water and fruit). Also depicts the input of other capital (e.g. human and built) to produce 'benefits'.
Loring et al 2008	A2.23	Loring, P.A., Chapin III, F.S. & Gerlach, S.C. (2008) The Services-oriented architecture: Ecosystem services as a framework for diagnosing change in social ecological systems. *Ecosystems,* 11: 478-489	Factors that guide agency and path dependence in ecosystem service use	To support a diagnostic approach for evaluating stability and change in ES using principles of computational thinking and services-orientated architecture.	Links ecosystem services with consumer and provider behaviour, through four pathways: service viability, service execution context, service interactions and service outcomes.

Turner & Daily 2008	A2.24	Turner, R.K & Daily, G.C. (2008) The ecosystem services framework and natural capital conservation. *Environmental and Resource Economics,* 39:25–35	The Ecosystem Services Framework (ESF)	To aid with ES-based decision-making processes.	Includes several processes, starting with identification and scaling through to post-policy appraisal. Consists of an ES and governance component, both affected by environmental change processes, and includes socio-economic elements (e.g. valuation and stakeholder participation).
Carpenter et al 2009	A2.25	Carpenter, R.S., Mooney, H.A., Agard, J., Capistrano, D., DeFries, R.S., Diaz, S. et al. (2009) Science for managing ecosystem services: Beyond the Millennium Ecosystem Assessment. *Proceedings of the National Academy of Sciences of the United States of America*, 106: 1305–1312.	Multi-scale relationship between governance and ecosystem services	To provide guidance for matching the scales of ecosystem governance to those of ecosystem dynamics, and to aid integration of social and ecological disciplines.	Depicts multi-scale linkages (local, regional, global) between governance and ecosystems, with emphasis on local feedbacks between local ecosystems and services, local governance, and community and individual well-being.
Daily et al 2009	A2.26	Daily, G.C., Polasky, S., Goldstein, J., Kareiva, P.M., Mooney, H.A., Pejchar, L. et al. (2009) The role of ecosystem services in conservation and resource management. *Frontiers in Ecology and the Environment*, 7:21-28	An iterative process for integrating ecosystem services into decisions	To aid integration of ES into decision-making, and used in the InVEST tool, which informs managers /policy makers about the impacts of alternative resource management choices.	Includes stakeholder engagement through several components – staging, scenarios (e.g. relating to change in management, population or climate), models (for quantifying and mapping ecosystem services) and outputs (biophysical, economic cultural).
Daily et al 2009	A2.27	Daily, G.C., Polasky, S., Goldstein, J., Kareiva, P.M., Mooney, H.A., Pejchar, L. et al. (2009) The role of ecosystem services in conservation and resource management. *Frontiers in Ecology and the Environment*, 7:21-29	A framework showing how ecosystem services can be integrated into decision making	To aid integration of ES into decision-making.	Includes five key components: decisions, ecosystems, services, values and institutions, with linking mechanisms such as models, information, action and scenarios, and incentives.
Fisher et al 2009	A2.28	Fisher, B., Turner, R.K. & Morling, P. (2009) Defining and classifying ecosystem services for decision making. *Ecological Economics,* 68:643-653	Conceptual relationship between intermediate and final services	To provide an operational, meaningful and consistent definition of ecosystem services.	Very similar to Fisher et al (2008), this framework shows the relationship between intermediate services, final services and benefits, along with the contribution of other forms of capital to realize these benefits.
Fisher et al 2009	A2.29	Fisher, B., Turner, R.K. & Morling, P. (2009) Defining and classifying ecosystem services for decision making. *Ecological Economics,* 68:643-654	Possible spatial relationships between service production areas and service benefit areas	Could be used to inform where management interventions should be concentrated.	Depicts four scenarios of service production/benefit areas, including service provision and benefit occurring in the same area, and services providing benefits omni-directionally to the surrounding landscape.

McLeod & Leslie 2009	A2.30	McLeod, K. & Leslie, H. (eds) (2009) *Ecosystem-Based Management for the Oceans*. Island Press: Washington, DC	Dynamic human and ecological systems referred to as 'coupled socio-ecological systems'.	To demonstrate the link between social and ecological systems in order to explain the relevance of ecosystem-based management (with particular reference to ocean ecosystems)	Ecosystem services link the ecological and social domains. Within each domain, multiple scales are depicted.
Robinson et al 2009	A2.31	Robinson, D.A., Libron, I. & Vereecken, H. (2009) On the definition of the natural capital of soils: a framework for description, evaluation, and monitoring. *Soil Science Society of America Journal,* 73: 1904–1911	A schematic diagram indicating an integrated process/economic modelling framework.	To demonstrate how natural capital fits within the ecosystem services framework and how it can be evaluated through integrated valuation and process-based models. Overall context of the research relates to soil natural capital.	Depicts the relationship between ecosystem natural capital, ecosystem services and valuation, and regional decision-making, with particular reference to valuation/modelling tools. Three spatial scales (regional, national and international) are depicted as interacting, in terms of information transfer.
Bryan et al 2010	A2.32	Bryan, B.A., Grandgirard, A. & Ward, J.R. (2010) Quantifying and exploring strategic regional priorities for managing natural capital and ecosystem services given multiple stakeholder perspectives. *Ecosystems*, 13:539–555	Ecosystem service-based goals hierarchy with individual services grouped into four ES types.	To categorise ES (through a participatory process) to provide structure for the quantification of management priorities.	Based on the MEA (2005), ES are categorised into four types- provisioning, regulating, supporting and cultural. Three new ES are added - 'Geological Resources', 'Energy' and 'Bequest, Intrinsic and Existence values'.
Collins et al 2010	A2.33	Collins, S.L., Carpenter, S.R., Swinton, S.M., Orenstein, D.E., Childers, D.L., Gragson, T.L. et al (2010) An integrated conceptual framework for long-term social–ecological research. *Frontiers in Ecology and the Environment,* 9: 351–357	Press–Pulse Dynamics Framework	To build transdisciplinary knowledge of social–ecological systems and contribute to the development and testing of theory within these disciplines.	Consists of a social template (human behaviour and human outcomes) and a biophysical template (community structure and ecosystem function), linked together through ecosystem services and by pulse and press events ('press' referring to extensive, pervasive, and subtle change, and 'pulse' referring to sudden events). The systems are influenced by external drivers such as climate and globalisation.
De Groot et al 2010a	A2.34	de Groot, R.S., Alkemade, R., Braat, L., Hein, L. & Willemen, L. (2010a). Challenges in integrating the concept of ecosystem services and values in landscape planning, management and decision making. *Ecological Complexity*, 7: 260–272	Framework for integrated assessment of ecosystem and landscape services.	Used in the Speerpunt Ecosystem & Landscape Services project to aid the integration of ES and values into landscape planning, management and decision making.	Links ecosystem and landscape character to five themes – services, values, trade-off analysis, planning tools and financing mechanisms.

De Groot et al 2010b	A2.35	de Groot, R., Fisher, B., Christie, M., Aronson, J., Braat, L., Gowdy J, et al. (2010b) *Integrating the ecological and economic dimensions in biodiversity and ecosystem service valuation*. In: Kumar, P. (ed.) The Economics of Ecosystems and Biodiversity: Ecological and Economic Foundations, London: Earthscan, 9–40.	The pathway from ecosystem structure and processes to human well-being	Designed to aid The Economics of Ecosystems and Biodiversity (TEEB) to disentangle the pathway from ecosystems and biodiversity to human wellbeing.	Depicts the pathway from biophysical structure or processes to human values, using the cascade model of Potschin & Haines-Young (2010). Also includes the influence of institutions and human judgments with regards to ecosystem services.
De Groot et al 2010b	A2.36	de Groot, R., Fisher, B., Christie, M., Aronson, J., Braat, L., Gowdy J, et al. (2010b) *Integrating the ecological and economic dimensions in biodiversity and ecosystem service valuation*. In: Kumar, P. (ed.) The Economics of Ecosystems and Biodiversity: Ecological and Economic Foundations, London: Earthscan, 9–40.	Conceptual framework for linking ecosystems and human well-being	Conceptual framework of The Economics of Ecosystems and Biodiversity (TEEB) to provide a basis for the TEEB report, in relation to linking ecosystems with human well-being.	Includes the interlinking components of: ecosystems and biodiversity; services; human well-being; governance and decision-making; and direct/indirect drivers. Based on the MA (2005a) overall conceptual framework.
Feld et al 2010	A2.37	Feld, C.K., Sousa, J.P., Martins da Silva, P. & Dawson, T.P. (2010) Indicators for biodiversity and ecosystem services: towards an improved framework for ecosystems assessment. *Biodiversity & Conservation,* 19:2895–2919	Schema showing the interdependence of the indicator suitability criteria	To support the development of suitable indicators for ecosystem assessment and monitoring.	Links seven indicator suitability criteria, relating to: purpose; indicator type; linkages to biodiversity and ES; spatial scale and scalability; threshold/references values; data and protocols; and remote sensing.
Haines-Young & Potschin 2010	A2.38	Haines-Young, R., & Potschin, M. (2010) *The links between biodiversity, ecosystem services and human well-being*. In: Raffaelli, D. and Frid, C. (eds) Ecosystem Ecology: A New Synthesis. BES Ecological Reviews Series, CUP. Cambridge: Cambridge University Press, 110–139.	The relationship between biodiversity, ecosystem function and human well-being.	To summarise the current thinking regarding ES and to also frame several important questions about the relationships between people and nature.	Depicts the relationship between biophysical structures and processes with human values, through a series of intermediate stages (including function, services and benefits), similar to a production chain.

Haslett et al 2010	A2.39	Haslett, J.R., Berry, P.M., Bela, G., Jongman, R.H.G., Pataki, G., Samways, M.J. & Zobel, M. (2010) Changing conservation strategies in Europe: a framework integrating ecosystem services and dynamics. *Biodiversity & Conservation*, 19:2963–2977	A framework for conservation in Europe integrating Ecosystem Services.	To join conventional biodiversity conservation with new requirements, particularly the demands of dynamic ecosystems and societal needs, in the context of European conservation.	Consists of an inner loop depicting traditional conservation strategy and a wider outer loop which includes societal needs and ES provision.
Klug & Jennewien 2010	A2.40	Klug, H. & Jennewien, P. (2010) Spatial modelling of agrarian subsidy payments as an input for evaluating changes of ecosystem services. *Ecological Complexity*, 7:368–377	Ecosystem goods and services unitized into three service categories: green, blue, and yellow services	To establish an inventory of spatially explicit environmental goods and services present in a landscape, for use in spatial modelling.	ES are categorised into provisioning, regulating, cultural and supporting services, and their connection with categories of green (environmental and landscape), yellow (socio-economic) and blue (water resource) services is shown.
López-Hoffman et al 2010	A2.41	López-Hoffman, L., Varady, R.G., Flessa, K.W. & Balvanera, P. (2010) Ecosystem services across borders: a framework for transboundary conservation policy. *Frontiers in Ecology and the Environment*, 8:84-91	Transboundary ecosystem services (shared by the US and Mexico).	Adapted from the MEA main conceptual framework, in order to frame ES in terms of transboundary conservation.	Consists of feedback loops between society/human well-being and biodiversity/ecosystems, interacting by means of ES and indirect/direct drivers. Two example loops are given in a global context (US and Mexico) to demonstrate the spatial interactions through transboundary interventions.
Maynard et al 2010	A2.42	Maynard, S., James, D. & Davidson, A. (2010) The development of an ecosystem services framework for South East Queensland. *Environmental Management,* 45:881–895	The process and key components of The South East Queensland (SEQ) Ecosystem Services Framework	To provide the tools to enable government, industry, business, researchers, non-government organizations and land managers to apply the concept of ecosystem services in their planning and management practices.	Depicts a set of processes linking components such as geographic location, ecosystem services and value or Relative Importance of Benefits. It also shows the participatory process involved in development of the framework.
Paetzold et al 2010	A2.43	Paetzold, A., Warren, P.H. & Maltby, L.L. (2010) A framework for assessing ecological quality based on ecosystem. *Ecological Complexity*, 7:273–281	Major steps in the development of an Ecosystem Services Profile (ESP)	To provide a way of assessing ecosystem quality which incorporates societal expectations for and the sustainable provision of ES, thus fostering a more integrative approach to ecosystem assessment and management.	Consists of six stages for the development of an Ecosystem Services Profile (ESP) (e.g. quantifying levels of provision and demand for ES), which involves stakeholder participation.

Potschin and Haines-Young 2010	A2.44	Potschin, M.B. & Haines-Young, R. H. (2010) Ecosystem services: Exploring a geographical perspective. *Progress in Physical Geography,* 35: 575–594	The ecosystem service cascade model	To summarise the current thinking regarding ES and to also frame several important questions about the relationships between people and nature.	Depicts the relationship between biophysical structures and processes with human values, through a series of intermediate stages (including function, services and benefits), similar to a production chain. Slightly modified from Haines-Young & Potschin (2010), including distinguishing between benefits and values.
Rounsevell et al 2010	A2.45	Rounsevell, M.D.A., Dawson, T.P. & Harrison, P.A. (2010) A conceptual framework to assess the effects of environmental change on ecosystem services. *Biodiversity & Conservation*, 19: 2823–2842	A Framework for Ecosystem Service Provision (FESP)	To aid the assessment of the effects of environmental change on ecosystem services provision.	Based on a Driver-Pressure-State-Impact-Response (DPSIR) framework, and thus comprised of each of these components. The 'state' component includes the supporting system as well as ecosystem services beneficiaries and providers. A stepwise implementation strategy for the conceptual framework is also shown.
Schreckenberg et al 2010	A2.46	Schreckenberg, K., Camargo, I., Withnall, K., Corrigan, C., Franks, P., Roe, D., et al (2010) *Social Assessment of Conservation Initiatives: A Review of Rapid Methodologies*. International Institute for Environment and Development (IIED): London.	The Modified Sustainable Livelihoods Framework	To highlight key components necessary or desirable to guide impact social impact assessments.	Consists of six sets of assets (social/cultural, physical, human, financial, natural and political/legal) and related opportunities that together help determine livelihood strategies. Also includes vulnerability context and drivers as additional influencing factors.
White et al 2010	A2.47	White, P.C.L., Godbold, J.A., Solan, M., Wiegand, J. & Holt, A.R. (2010) *Ecosystem services and policy: a review of coastal wetland ecosystem services and an efficiency-based framework for implementing the ecosystem approach*. In: Hester, R.E. & Harrison, R.M. (Eds) Ecosystem Services. Issues in Environmental Science and Technology, 30:29-51 http://dx.doi.org/10.1039/9781849731058-00029.	Efficiency framework for an ecosystem services approach to sustainability	To provide a conceptual basis for assessing the components of social-ecological systems and the links between them, based around magnitudes and efficiencies of conversion between states.	Consists of three broad sub-systems: ecosystem functions, ecosystem services, and social development and well-being. These systems interact with each other (e.g. through impacts, consumption, and trade-offs), representing a transfer of state (e.g. from ecosystem functions to ecosystem services). Within each sub-system feedback loops and mechanisms (e.g. governance, incentives etc.) are depicted.
Atkins et al 2011	A2.48	Atkins, J.P., Burdon, D., Elliott, M. & Gregory, A.J. (2011) Management of the marine environment: Integrating ecosystem services and societal benefits with the DPSIR framework in a systems approach. *Marine Pollution Bulletin*, 62:215–226	The DPSIR framework and the ecosystem services and societal benefits set within an overall framework of The Ecosystem Approach	To integrate the DPSIR framework with ecosystem services and societal benefits to help support decision-making in environmental management (with particular reference to the marine environment).	Consists of two frameworks sitting within an overall ecological approach: (i) the DPSIR (Drivers–Pressures–State Change–Impact–Response) approach, which can 'protect the natural system & benefits for society', and (ii) ES and social benefits, which consists of biota-ecological structure, physico-chemical, and biota-ecological functioning, which interact and deliver benefits for society.

Balmford et al 2011	A2.49	Balmford, A., Fisher, B., Green, R.E., Naidoo, R., Strassburg, B., Turner, R.K. & Rodrigues, A. S. L. (2011) Bringing ecosystem services into the real world: an operational framework for assessing the economic consequences of losing wild nature. *Environmental & Resource Economics*, 48:161–175	Framework for assessing the economic consequences of losing biodiversity and ecosystems	To aid the quantification of how the loss of ES and biodiversity compares with the costs incurred in retaining them, to help inform policy decisions.	Consists of five key elements - drivers of loss, state of the world, policy, natural science and economics - which form interlinked processes for assessing the differences in costs and benefits between two hypothetical states of the world.
Balmford et al 2011	A2.50	Balmford, A., Fisher, B., Green, R.E., Naidoo, R., Strassburg, B., Turner, R.K. & Rodrigues, A. S. L. (2011) Bringing ecosystem services into the real world: an operational framework for assessing the economic consequences of losing wild nature. *Environmental & Resource Economics,* 48:161–175	Illustration of the relationship between core ecosystem processes, beneficial ecosystem processes, and ecosystem benefits	To categorise ecosystem processes and benefits in a manner which avoids double-counting.	ES are disaggregated into three interlinking sets - core ecosystem processes, beneficial ecosystem processes and ecosystem benefits - whereby *processes* are biophysical functions, while *benefits* are the end goods and services that directly affect the human welfare function.
Comello & Lepech 2011	A2.51	Comello, S.D. & Lepech, M.D. (2011) *A framework for multiphysics modeling of natural environments for valuation of privately owned ecosystem services.* IEEE International Symposium on Sustainable Systems and Technology (ISSST), 16-18 May 2011, Chicago, IL	Firm-level ecosystem service valuation framework	To incentivise firms to incorporate environmental considerations into project development or management decisions, thus encouraging more sustainable management and development.	Depicts the relationship between business/ firm-level activity, ecosystem function impact, change in ES and change in ES value, and four assessment/analysis processes that firms need to carry out (Life Cycle Assessment, ecosystem function assessment, substitutability and decision analysis).
Daw et al 2011	A2.52	Daw, T., Brown, K., Rosendo, S. & Pomeroy, R. (2011) Applying the ecosystem services concept to poverty alleviation: the need to disaggregate human well-being. *Environmental Conservation*, 38: 370–379.	Conceptualizations of ES and human well-being that do not disaggregate human well-being (i.e. the beneficiaries).	To highlight the importance of disaggregating human well-being in ES management to help inform ES interventions (e.g. payments for ES) that contribute to poverty alleviation.	Simple diagram to illustrate three scenarios: (a) aggregated view of well-being and ES, (b) elements of human well-being are disaggregated, recognizing that different ES may contribute to different elements of well-being, and (c) ES are disaggregated to explore trade-offs between them.

Daw et al 2011	A2.53	Daw, T., Brown, K., Rosendo, S. & Pomeroy, R. (2011) Applying the ecosystem services concept to poverty alleviation: the need to disaggregate human well-being. *Environmental Conservation*, 38: 370–379.	Aspects of ES and human well-being relevant to poverty alleviation that are highlighted by disaggregating human beneficiaries of ES.	To highlight the importance of disaggregating human well-being in ES management to help inform ES interventions (e.g. payments for ES) that contribute to poverty alleviation.	Four scenarios are depicted that show ES flow to two potential beneficiaries (A and B) i.e. disaggregating human beneficiaries of ES. The scenarios show how ES flow to the different beneficiaries are affected by factors such as trade-offs, access barriers, well-being-context such as wealth, and indirect benefits through payments for consumption.
Kumar et al 2011	A2.54	Kumar, R., Horwitz, P., Milton, G.R., Sellamuttu, S.S., Buckton, S.T., Davidson, N.C. et al (2011) Assessing wetland ecosystem services and poverty interlinkages: a general framework and case study. *Hydrological Sciences Journal,* 56:1602-1621	Framework for assessing wetlands–livelihood interlinkages.	To support the assessment of ecological management goals and interlinkages with poverty reduction and sustainable livelihoods, in the context of wetland management.	Consists of several components- central being the ecosystem settings (comprised of 'capitals' and 'ecological character'), which sits within institutions and freedoms. This is linked to livelihood outcomes, via livelihood strategies. The ecosystem settings are also affected by the vulnerability context (drivers of change).
UK NEA 2011	A2.55	UK National Ecosystem Assessment (UK NEA) (2011) *UK National Ecosystem Assessment Technical Report.* UNEP-WCMC: Cambridge	The overall conceptual framework for the UK NEA	Used to underpin the UK NEA, based on the MA overall conceptual framework.	Demonstrate the links between ecosystems, ecosystem services, goods, human well-being, and drivers of change
UK NEA 2011	A2.56	UK National Ecosystem Assessment (UK NEA) (2011) *UK National Ecosystem Assessment Technical Report*. UNEP-WCMC: Cambridge	The full set of ecosystem processes, services, goods/benefits and values used in the UK NEA	Used to underpin the UK NEA, providing classifications of ecosystem services and goods.	Illustrates the relationship between ecosystem processes, services and goods and human well-being, which is valued in terms of economic, health and shared social values. Adapted from Fisher et al (2008).
Wainger & Mazzotta 2011	A2.57	Wainger, L. & Mazzotta, M. (2011) Realizing the potential of ecosystem services: a framework for relating ecological changes to economic benefits. *Environmental Managemen*t, 48: 710–733	Framework to estimate economic benefits of a management change	To provide guidance for producing ecological models /metrics for assessing economic benefits of policy/management changes.	Links a change in human action to a change in ecosystem stressors or condition, impacting upon ecological outcome which affects ecosystem services, which in turn affects social benefits.
Bastian et al 2012a	A2.58	Bastian, O., Hasse, D. & Grunewald, K. (2012a) Ecosystem properties, potentials and services – The EPPS conceptual framework and an urban application example. *Ecological Indicators*, 21:7–16	Ecosystem Properties, Potentials, and Services (EPPS) framework for the analysis of ecosystem/ landscape services	Drawing upon the landscape potential concept, the authors aim to provide an improved approach for ES assessment, and for linking ecosystem services and potentials to management practice.	Consists of three inter-related pillars - properties, potentials and services - of ecosystems & landscapes. Further details for each pillar are depicted, including the nature of the indicators. Additionally, driving forces affecting the pillars are illustrated, and the type of evidence/assessment (factual/valuation).

Bastian et al 2012b	A2.59	Bastian, O., Grunewald, K. & Syrbe, R.U (2012b) Space and time aspects of ecosystem services, using the example of the EU Water Framework Directive. *International Journal of Biodiversity Science, Ecosystem Services & Management*, 8: 5–16	Selected spatially relevant phenomena reflecting different scales.	To improve the incorporation of spatial aspects in ES management.	A range of different ecological structures or types of space are depicted on a spatial scale and an axis of natural/physical space to constructed space.
Bowd et al 2012	A2.60	Bowd, R., Quinn, N., Kotze, D.C., Hay, D.G. & Mander M. (2012) The identification of potential resilient estuary-based enterprises to encourage economic empowerment in South Africa: a toolkit approach. *Ecology and Society,* 17:15	A framework for analysing the robustness of social-ecological systems from an institutional perspective (showing examples of how it relates to an estuarine system)	To aid the transition between conceptual frameworks/ theory, to practical integration of ES into decision-making, through translating a conceptual framework into a practical toolkit.	Consists of four key components: the resource, the resource users, public infrastructure, and public infrastructure providers. The components are linked by four steps - Step 1 - ES supply and demand assessment; Step 2 - future estuary roles identification; Step 3 - enterprise opportunity identification; Step 4 - enterprise risk assessment. Estuarine example used but easily generalised. Adapted from a social-ecological system framework by Anderies et al. (2004).
Chan et al 2012a	A2.61	Chan, K.M.A., Satterfield,T. & Goldstein, J. (2012a) Rethinking ecosystem services to better address and navigate cultural values. *Ecological Economics,* 74: 8–18	The suggested use of the typologies of ecosystem services and values	To improve the incorporation of non-monetary/non-material, cultural values into decision-making.	Presents new typologies of ecosystem services, benefits and values, including a new set of eight value dimensions. The framework demonstrates the suggested use of the typologies and the links between services, benefits and values.
Chan et al 2012b	A2.62	Chan, K.M.A., Guerry, A.D., Balvanera, P., Klain, S., Satterfield, T., Basurto, X. et al (2012b) Where are cultural and social in ecosystem services? A framework for constructive engagement. *BioScience,* 62:744–756.	Framework for characterizing ES that might be affected by management or planning.	To facilitate the incorporation of diverse values associated with ecological and socioecological change, in order to aid decision-making, management and planning.	Five steps to aid ES management and planning are illustrated: 1- obtain consent, 2- determine the decision context, 3- determine the socio-ecological context, 4- determine the ES, benefits and values, and 5- influence diagrams and scenarios.
Comello et al 2012	A2.63	Comello, S. D., Lepech, M. D. & Schwegler, B. R. (2012) Project-level assessment of environmental effect : ecosystem services approach to sustainable management and development. *Journal of Management in Engineering,* 28:5-12	Firm-level ecosystem service valuation framework	To incentivise firms to incorporate environmental considerations into project development or management decisions, thus encouraging more sustainable management and development.	Depicts the relationship between four assessment/ valuation stages to be undertaken by a firm: (i) determine lifecycle inventory, (ii) assess ecosystem functions, (iii) perform functional substitutability and (iv) determine economic value. This is followed by decision analysis. Adapted from Comello & Lepech (2011).

Luck et al 2012	A2.64	Luck, G.W., Lavorel, S., McIntyre, S. & Lumb, K. (2012) Improving the application of vertebrate trait-based frameworks to the study of ecosystem services. *Journal of Animal Ecology*, 81:1065–1076	Trait-selection framework	To use a trait-selection approach to investigate species' response to environmental change and the consequences for the provision of ES.	Presents four decision stages relating to the selection of species traits from a full set of traits (pool 1) to a selected set of traits for assessing the impact of environmental change on ES (pool 5).
Bastian et al 2013	A2.65	Bastian, O., Syrbe, R.U., Rosenberg, M., Rahe, D., & Grunewald, K. (2013) The five pillar EPPS framework for quantifying, mapping and managing ecosystem services. *Ecosystem Services,* 4:15–24	Conceptual framework for the analysis of ecosystem services—the extended Ecosystem Properties, Potentials, and Services (EPPS) framework	The EPPS framework aims to provide an improved approach for ES assessment, and for linking ecosystem services and potentials to management practice - this extended version adds additional aspects such as benefits/values, beneficiaries and management, as well as spatial and temporal aspects.	Consists of five inter-related pillars - properties, potentials, services, benefits/values and users/beneficiaries. These sit within three overlapping levels- physical, intermediate and socio-economic. The use and management of ES impact upon the five pillars. The temporal aspect of assessment (time scale, driving forces, changes and scenarios) is illustrated through a number of processes, and spatial aspects (spatial scales, dimensions, patterns) are also depicted. The type of evidence/assessment (factual/valuation) is also shown.
Ernstson 2013	A2.66	Ernston, H. (2013) The social production of ecosystem services: a framework for studying environmental justice and ecological complexity in urbanized landscapes. *Landscape and Urban Planning*, 109:7– 17	A framework for studying the social production of ecosystem services	To relate ecosystem services to environmental justice	Consists of two diagrams representing three analytical moments (generation, distribution- shown in diagram A, and articulation- shown in diagram B) which are studied through moving between two different levels/scales of analysis, with different sets of methods. The 'distribution' aspect, in particular, relates to environmental justice. Urban environment used as an example, but could be generalised.
Honrado et al 2013	A2.67	Honrado, J.P., Vieira, C., Soares, C., Monteiro, M.B., Marcos, B., Pereira, H.M. & Partidário, M.R. (2013) Can we infer about ecosystem services from EIA and SEA practice? A framework for analysis and examples from Portugal. *Environmental Impact Assessment Review*, 40:14–24	Criteria for explicit analysis for consideration of ecosystem services in Strategic Environmental Assessment (SEA).	To facilitate the incorporation of ES into Strategic Environmental Assessment (SEA), as ES are often not explicitly considered in SEA.	Consists of six criteria for explicit analysis in SEA: ecosystems, ES identification, stakeholders, drivers of change, benefits, and valuation.

Kelble et al 2013	A2.68	Kelble, C.R., Loomis, D.K., Lovelace, S., Nuttle, W.K., Ortner, P.B, Fletcher, P. et al. (2013) The EBM-DPSER Conceptual Model: integrating ecosystem services into the DPSIR framework. *PLoS ONE*, 8: e70766	The Ecosystem-Based Management, Driver, Pressure, State, Ecosystem service, and Response (EBM-DPSER) model	To facilitate the inclusion and operationalization of ES into holistic ecosystem-based management strategies, thus improving management by better capturing human-natural interactions and societal benefits.	Five interacting components - Ecosystem services, State, Pressures, Drivers, and Responses- are presented, described in more detail in each box.
Lavorel et al 2013	A2.69	Lavorel, S., Storkey, J., Bardgett, R.D., de Bello, F., Berg, M.P. Le Roux, X. (2013) A novel framework for linking functional diversity of plants with other trophic levels for the quantification of ecosystem services. *Journal of Vegetation Science,* 24:942–948	Method for articulating functional responses and effects within and across two trophic levels to predict changes in ecosystem functioning, and methodological steps for its application	Using a trait-selection approach, the framework helps to link functional diversity of plants with other trophic levels for the quantification of ES, in order to predict the impact of environmental change on ecosystem service delivery.	Consists of four steps to identify relevant traits: 1- Identify traits that respond to environmental driver of interest, 2- Identify the trophic effect and response traits of the lower and upper trophic levels respectively, 3- Define and identify appropriate metrics of functional effect traits that determine efficiency of service delivery, and 4- Analyse linkages among different response and effect traits within each trophic level.
Lopes & Videira 2013	A2.70	Lopes, R. & Videira, N. (2013) Valuing marine and coastal ecosystem services: an integrated participatory framework. *Ocean & Coastal Management*, 84:153-162	Integrated participatory framework for the valuation of marine and coastal ecosystem services	To provide guidance for a participatory/ deliberative approach to ecosystem services valuation through involvement of different stakeholder groups.	Comprised of three main stages within the overall 'policy formation and assessment process': 1) set the scene, 2) deepen understanding and 3) articulate values. This leads to decision-implementation. Set within marine/ coastal context, but the framework is not itself habitat specific.
Lopes & Videira 2013	A2.71	Lopes, R. & Videira, N. (2013) Valuing marine and coastal ecosystem services: An integrated participatory framework. *Ocean & Coastal Management*, 84:153-163	Decision-making process in marine and coastal environments	To illustrate a formalised decision-making process based on the integrated environmental management	Consists of four phases of decision-making related to values of ES: (i) problem identification, (ii) analysing the problem; different alternative solutions/scenarios, (iii) selecting best solution; converting decision into action; methods and instruments, and (iv) monitoring. Adapted from a framework on integrated environmental management of the oceans by Antunes & Santos (1999).
Maes et al 2013	A2.72	Maes, .J, Teller, A., Erhard, M., Liquete, C., Braat, L., Berry, P., et al (2013) *Mapping and assessment of ecosystems and their services. An analytical framework for ecosystem assessments under action 5 of the EU biodiversity strategy to 2020.* Publications office of the European Union, Luxembourg.	Conceptual framework for EU wide ecosystem assessments	To aid EU member states with mapping and assessing the state of ecosystems and their services.	Consists of an ecosystems and a socio-economic component, linked together via ecosystem services and drivers of change. The ecosystems component includes a biodiversity ‘butterfly’ diagram which emphasises the different dimensions of biodiversity that contribute to ecosystem functioning, and hence ecosystem services. The socio-economic component consists of human well-being (benefits and values) and well as response.

Morse et al 2013	A2.73	Morse, W.C., McLaughlin, W.J., Wulfhorst , J.D. & Harvey, C. (2013) Social ecological complex adaptive systems: a framework for research on payments for ecosystem services. *Urban Ecosystems,* 16:53–77	Structuration of social ecological complex adaptive systems (SECAS)	To link structuration theory (social sciences) with complex adaptive systems theories (natural sciences) to provide an enhanced understanding of the human drivers and responses to environmental change.	Illustrates the relationship between the social system, including actors' capacity, and the ecological system, which includes patch level and patch mosaics, along with the effect of action/disturbance, and feedbacks/time.
Reed et al 2013	A2.74	Reed, M.S., Podesta, G., Fazey, I., Geeson, N., Hessel, R., Hubacek, K., et al (2013) Combining analytical frameworks to assess livelihood vulnerability to climate change and analyse adaptation options. *Ecological Economics*, 94:66–77	An integrated analytical framework for analysing livelihood vulnerability to climate change	Combines several theoretical frameworks in order to produce an integrated framework to better assess the vulnerability of rural livelihoods to climate change.	Consists of four broad steps, broadly related to determining: (i) exposure to climate change, (ii) sensitivity of capital stocks and ES to climate change; (iii) factors influencing decisions surrounding adaptation strategies, and (iv) potential trade-offs between adaptation options. Correlating to these stages, the framework illustrates factors affecting stocks & flows of capital assets, and the decision-making process.
Reis et al 2013	A2.75	Reis, S., Morris, G., Fleming, L.E., Beck, S., Taylor, T., White, M., Depledge, M.H., Steinle, S., Sabel, C.E., Hurley, F., et al. (2013) Integrating health and environmental impact analysis. *Public Health*, 1-7	Ecosystem-enriched DPSEEA (eDPSEEA)- a conceptual framework for an integrated assessment of human and ecosystem health and ecosystem service provision	To improve the integration of human health and environmental impact analysis.	Links five components - driver, pressure, state, exposure/ experience and effect- with 'action'. 'State' is comprised of ES and 'Exposure/experience' is comprised of a number of determinants of human well-being.
Reis et al 2013	A2.76	Reis, S., Morris, G., Fleming, L.E., Beck, S., Taylor, T., White, M., Depledge, M.H., Steinle, S., Sabel, C.E., Hurley, F., et al. (2013) Integrating health and environmental impact analysis. *Public Health,* 1-7	Illustrating the potential for feedback loops between Pressure, State and Exposure/ Experience which is manifest when considering relationships between ES and determinants of human health and well-being	To improve the integration of human health and environmental impact analysis.	Consists of three components- pressure, state and exposure, the latter two sitting within a broader context. Feedback between ES (within State) and experience (within Exposure) is illustrated.

Wang et al 2013	A2.77	Wang, S., Fu, B., Wei, Y. & Lyle, C. (2013) Ecosystem services management: an integrated approach. *Current Opinion in Environmental Sustainability,* 5:11-15	A framework of relationships between the natural ecosystem and the social and economic systems	To encourage/ illustrate a new macroeconomic paradigm which protects ES and leads to sustainable use of natural ecosystem, through integrating market, government and human values.	The natural ecosystem is shown to support the social and the economic systems through the delivery of ES, but is negatively affected by human impacts. The market regulates the economic system (consisting of consumer, firm, product and money), and government regulates the social system. Human values are identified as the 'third hand,' which when working with an integrated system of markets, governments, and human values result in the provision of sustainable ES.
Ahern et al 2014	A2.78	Ahern, J., Cillier, S. & Niemelä, J. (2014) The concept of ecosystem services in adaptive urban planning and design: A framework for supporting innovation. *Landscape and Urban Planning*, 125:254–259	A transdisciplinary adaptive design and planning model	To demonstrate how science, professional practice and stakeholder participation can be better integrated, in order to improve strategies for ES management and delivery (set in urban context but framework is generalizable).	Consists of six steps/actions (for example, defining ES goals, and monitoring & evaluation) which are shown to link with 'actors' (including planning & design professionals, scientists and transdisciplinary processes).
Clark et al 2014	A2.79	Clark, N.E., Lovell, R., Wheeler, B.W., Higgens, S.L., Depledge, M.H. & Norris, K. (2014) Biodiversity, cultural pathways, and human health: a framework. *Trends in Ecology & Evolution*, 29:198-204	The direct and indirect (cultural) pathways from biodiversity to human health	To illustrate the link between biodiversity change with human cultural values, well-being, and health, which may be important for biodiversity conservation and public health.	Illustrates the direct effects of biodiversity on human health (e.g. the regulation of the emergence and transmission of disease and pollution control) and also the indirect effects of biodiversity on human health via cultural pathways - biodiversity loss affects the provision of cultural goods, which reduces their value and, consequently negatively impacts upon human well-being and health.
Comello et al 2014	A2.80	Comello, S.D., Maltais-Landry, G., Schwegler, B.R. & Lepech, M.S. (2014) Firm-level ecosystem service valuation using mechanistic biogeochemical modeling and functional substitutability. *Ecological Economics,* 100:63-73	Firm-level ecosystem service valuation framework	To incentivise firms to incorporate environmental considerations into project development or management decisions, thus encouraging more sustainable management and development.	Depicts the relationship between four 'framework stages': business/ firm-level activity, ecosystem function impact, change in ES and change in ES value. Also shows five 'framework tools' that firms need to carry out (Life Cycle Assessment, ecosystem function assessment, functional substitutability & exergy analysis, corporate finance and decision analysis & corporate judgement). Adapted from Comello & Lepech (2011) and Comello et al (2012).

Duraiappah et al 2014	A2.81	Duraiappah, A.K., Asah, S.T., Brondizio, E.S., Kosoy, N., O'Farrell, P.J., Prieur-Richard A.H., et al. (2014) Managing the mismatches to provide ecosystem services for human well-being: a conceptual framework for understanding the New Commons. *Current Opinion in Environmental Sustainability,* 7:94–100	A multi-scale conceptual framework on nature, the productive base of societies and human well-being	To address the mis-matches and the multiple spatial scales of ES provision, to improve the understanding and assessment of key inter-linkages between nature and human well-being.	Consists of an interlinking social and ecological system at multiple scales. Both systems feed into the productive base and are affected by institutions and governance. The productive base contributes to human well-being, via human, productive and natural capital, the latter via ES provision. Human well-being also affects the productive base and nature's systems.
Mastrangelo 2014	A2.82	Mastrangelo, M.E., Weyland, F., Villarino, S.H., Barral, M.P., Nahuelhual, L. & Laterra, P. (2014) Concepts and methods for landscape multifunctionality and a unifying framework based on ecosystem services. *Landscape Ecology,* 29:345–358	Methodological approach proposed to guide research for the assessment of a socially-relevant, process-based landscape multifunctionality, and thus to inform landscape planning aimed at improving local well-being.	To inform landscape planning aimed at improving local well-being, through linking the concept of multifunctionality with the ES approach.	Depicts several stages and processes for assessing multifunctionality based on ES, several which involve stakeholder participation e.g. selection of relevant ES, valuation of relevant ES, scenario planning, and discussion & validation of scenarios.
Schröter et al 2014	A2.83	Schröter, M., Barton, D.N., Remme, R.P. & Hein, L. (2014) Accounting for capacity and flow of ecosystem services: a conceptual model and a case study for Telemark, Norway. *Ecological Indicators,* 36:539– 551	Integration of ES capacity and flow models in ecosystem accounting	To highlight the difference between the full potential of ecosystems to provide final services and the current use of it - and thus provide information for more sustainable land management.	Divided into three main components: (i) ecosystem assets (stock), (ii) ecosystem services, and (iii) society and economy (stocks & flows). These are linked via capacity and flows. The relevance of these for ecosystem accounting are also shown: (i) measurement of ecosystems, (ii) measurement of ES, and (iii) measure of other contributions to ES. An additional part of the framework - human inputs (ecosystem management) - highlights different model inputs.
Serna-Chavez et al 2014	A2.84	Serna-Chavez, H.M., Schulp , C.J.E., van Bodegom, P.M., Bouten, W., Verburg, P.H. & Davidson, M.D. (2014) A quantitative framework for assessing spatial flows of ecosystem services. *Ecological Indicators,* 39:24–33	Framework to analyse and quantify ecosystem service flows	To aid the analysis of the spatial connections between ES provisioning and benefiting areas, which is often lacking in ES assessments.	Consists of three overlapping circles. In the centre is P - representing ES provisioning areas. This sits within a larger circle - F, the flow area where ES can be potentially delivered from P. Two smaller ES 'benefiting' areas are also shown, which are spatial units in which ES are needed or readily used/ consumed.
