# Supplementary material for: Integrating Human and Ecosystem Health Through Ecosystem Services Frameworks
Source: Ecohealth. 2015 Sep 24;12:660–71. doi: 10.1007/s10393-015-1041-4 (PMC4700085; doi:10.1007/s10393-015-1041-4)
Supplement: Supplementary file 2 — Supplementary material 2 (PDF 7,275 kb) [file 10393_2015_1041_MOESM2_ESM.pdf]

Appendix 2. Ecosystem services frameworks (chronological order 1987-2014)

Figures A2.1-A2.84

All figures reproduced with permission

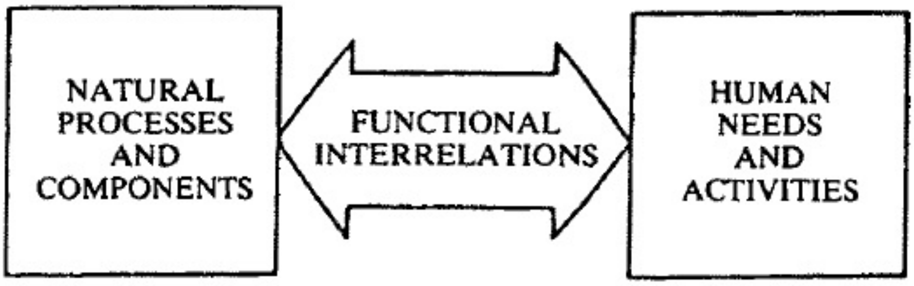

Figure A2.1. An early conceptual framework of ecosystem services. The functional interrelations in this figure represent the goods and services provided by the natural environment and, in the other direction, the impact of human activities on natural processes and components**.**

Source: de Groot, R.S. (1987) Environmental functions as a unifying concept for ecology and economics. *The Environmentalist*, 7:105-109
Figure on page 107. Reproduced with permission from Kluwer Academic Publishers.

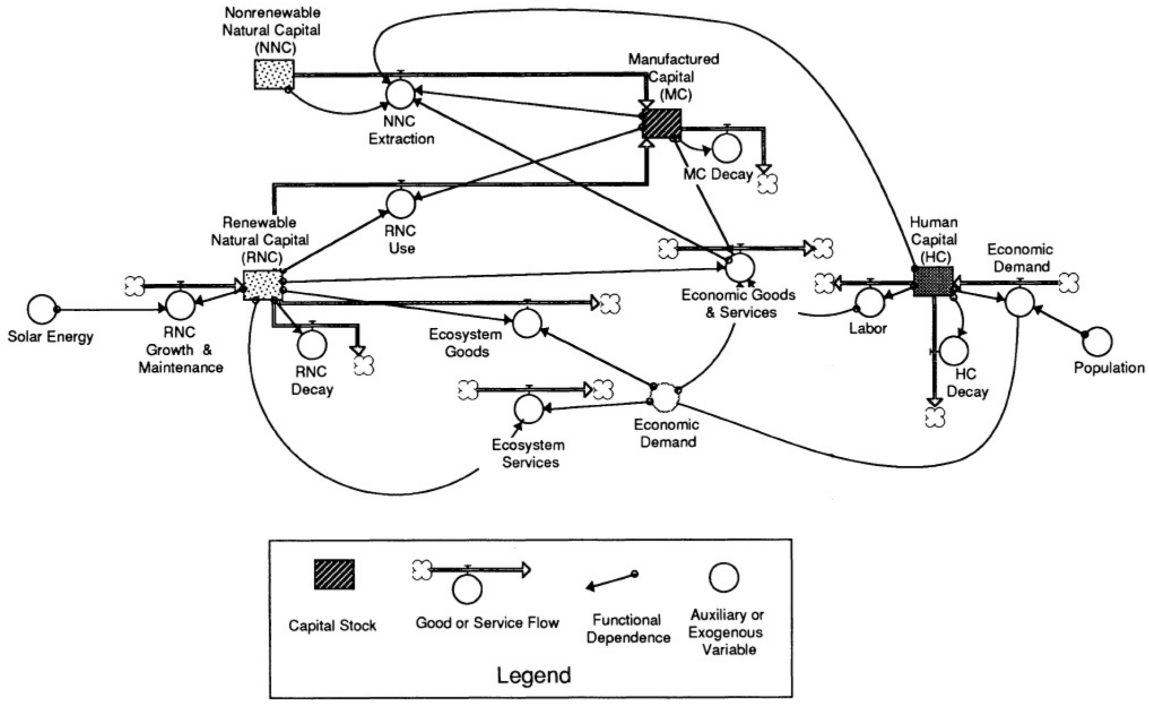

Figure A2.2. Types of natural and human-made capital stocks, good and service flows, and their interdependence.

Source: Costanza, R. & Daly, H.E. (1992) Natural capital and sustainable development. *Conservation Biology*, 6: 37-46
Figure 1, page 39. Reproduced with permission from John Wiley & Sons.

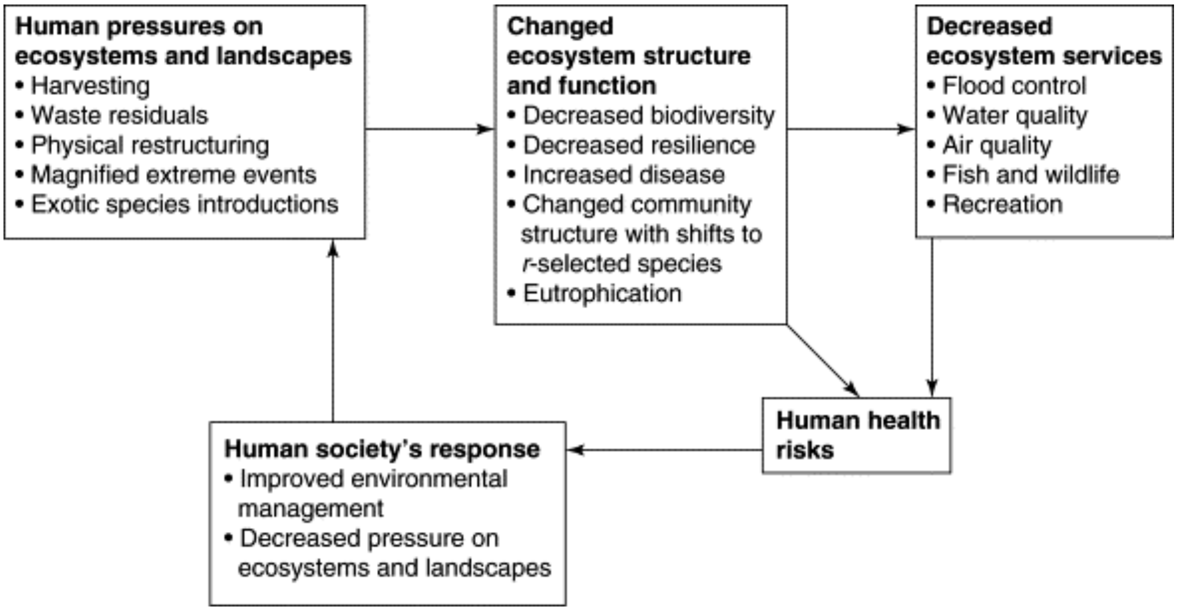

Figure A2.3. Linkages between pressures from human activity, ecosystem change and degradation of ecosystem and human health**.**

Source: Rapport, D.J., Costanza, R. & McMichael, A.J. (1998) Assessing ecosystem health. *Trends in Ecology and Evolution*, 13:397-402
Figure 1, page 399. Reproduced with permission from Elsevier.

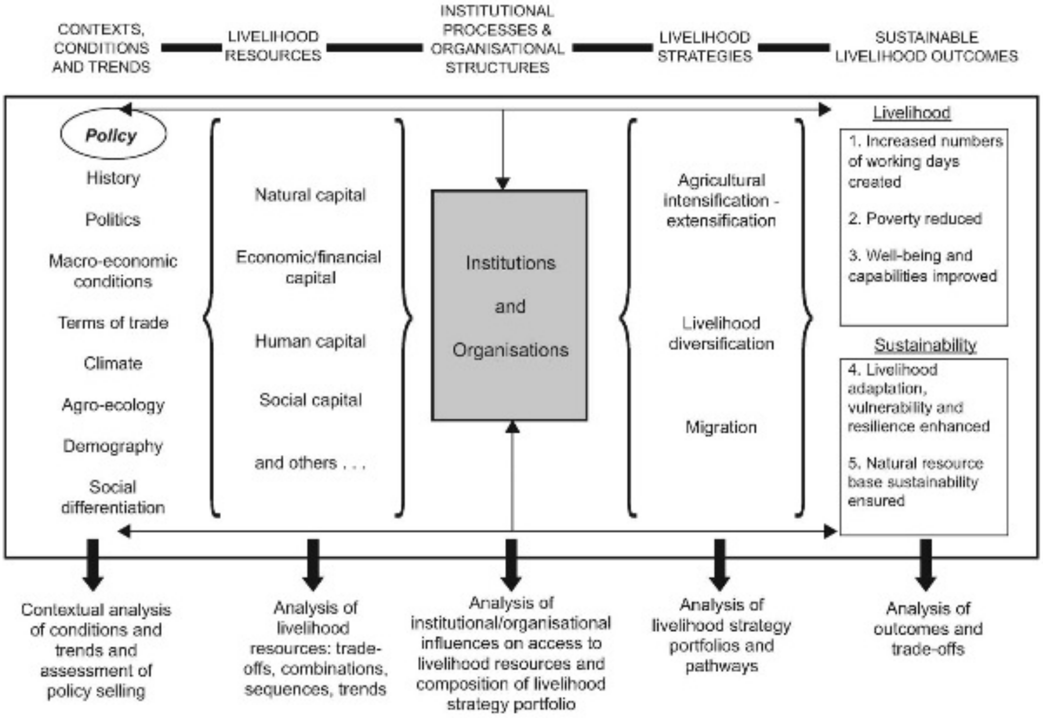

Figure A2.4. Sustainable livelihoods framework: a framework for analysis

Source: Scoones, I. (1998). Sustainable rural livelihoods: a framework for analysis. *IDS Working Paper,* 72: 1–22
Figure 1, page 4. Reproduced with permission from the Institute of Development Studies.

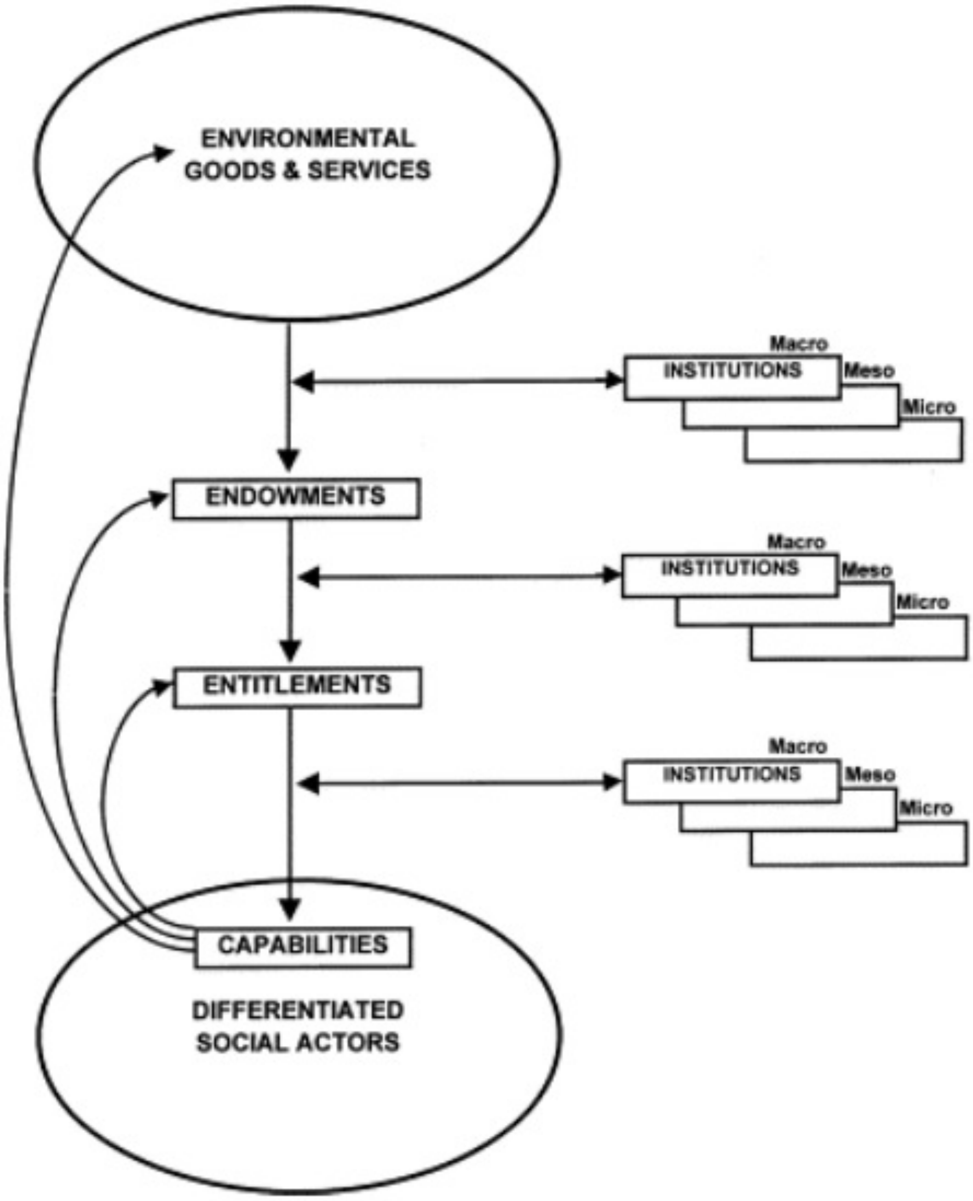

Figure A2.5 Environment entitlements framework.

Source: Leach, M., Mearns, R. & Scoones, I. (1999) Environmental entitlements: dynamics and institutions in community-based natural resource management. *World Development*, 2: 225-247
Figure 1, page 234. Reproduced with permission from Elsevier.

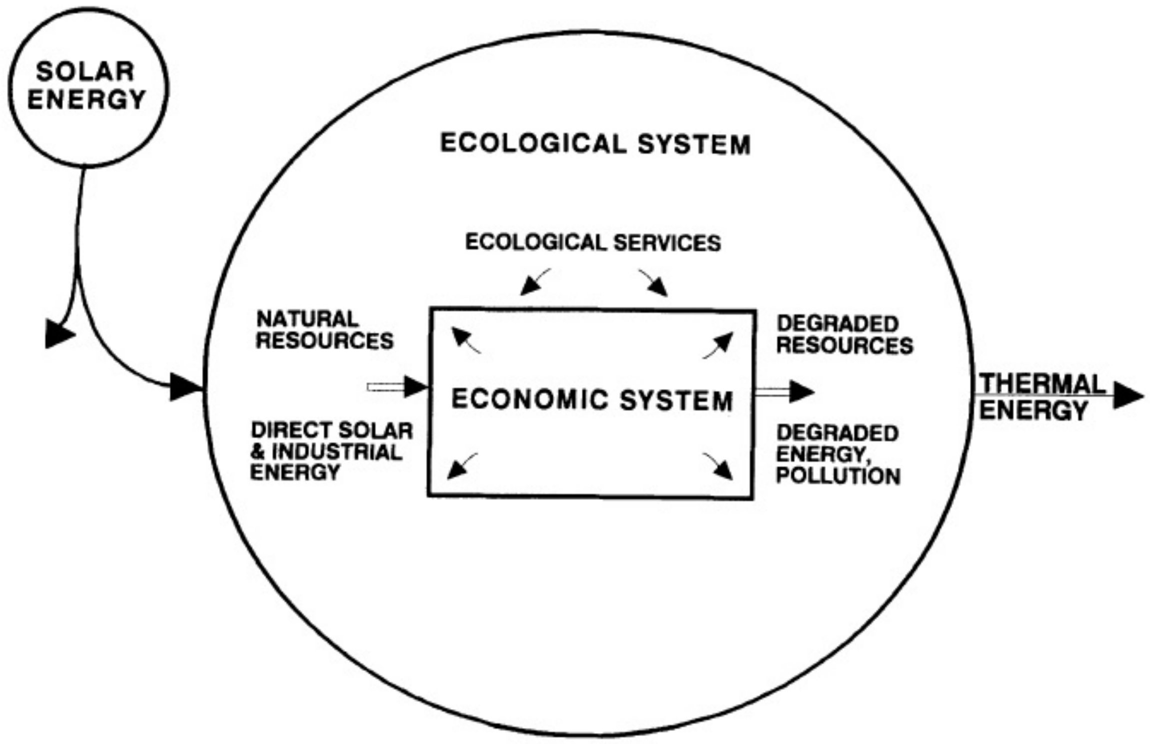

Figure A2.6. The economy as part of its life-supporting environment.

Source: Perrings, C., Folke, C. & Mäler, K.G. (2002) The ecology and economics of biodiversity loss: the research agenda. *Ambio,* 21: 201-211
Figure 1, page 201. Reproduced with permission from the Royal Swedish Academy of Sciences.

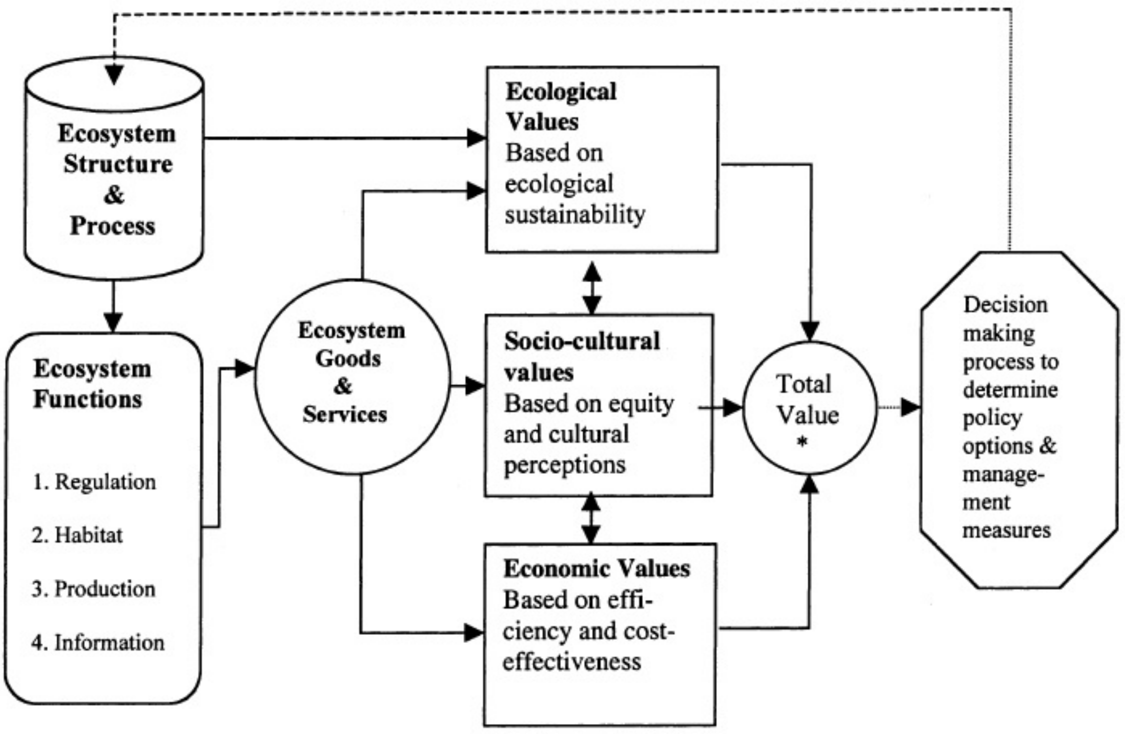

Figure A2.7. Framework for integrated assessment and valuation of ecosystem functions, goods and services

Source: de Groot, R.S., Wilson, M.A. & Boumans, R.M.J. (2002) A typology for the classification, description and valuation of ecosystem functions, goods and services. *Ecological Economics*, 41: 393–408
Figure 1, page 394. Reproduced with permission from Elsevier.

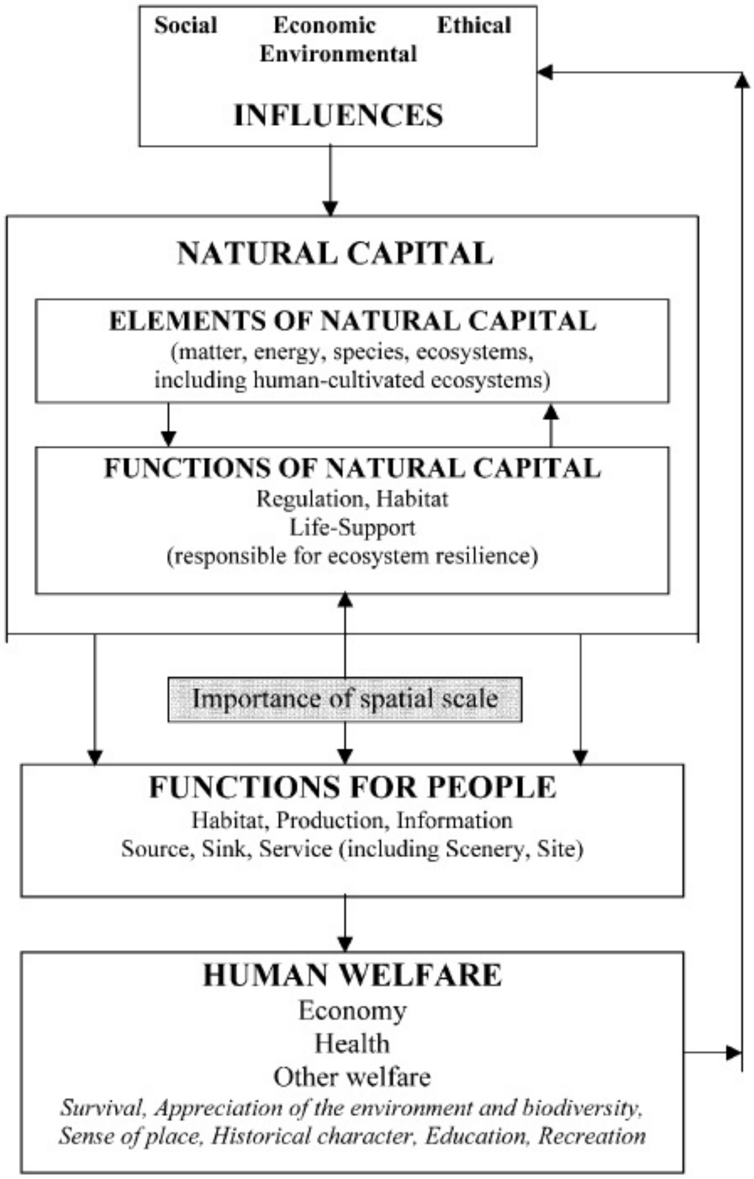

Figure A2.8. Environmental functions and attributes: human influences and welfare

Source: Ekins, P. Simon, S., Deutsch, L., Folke, C. & de Groot, R. (2003) A framework for the practical application of the concepts of critical natural capital and strong sustainability. *Ecological Economics*, 44: 165-185
Figure 1, page 172. Reproduced with permission from Elsevier.

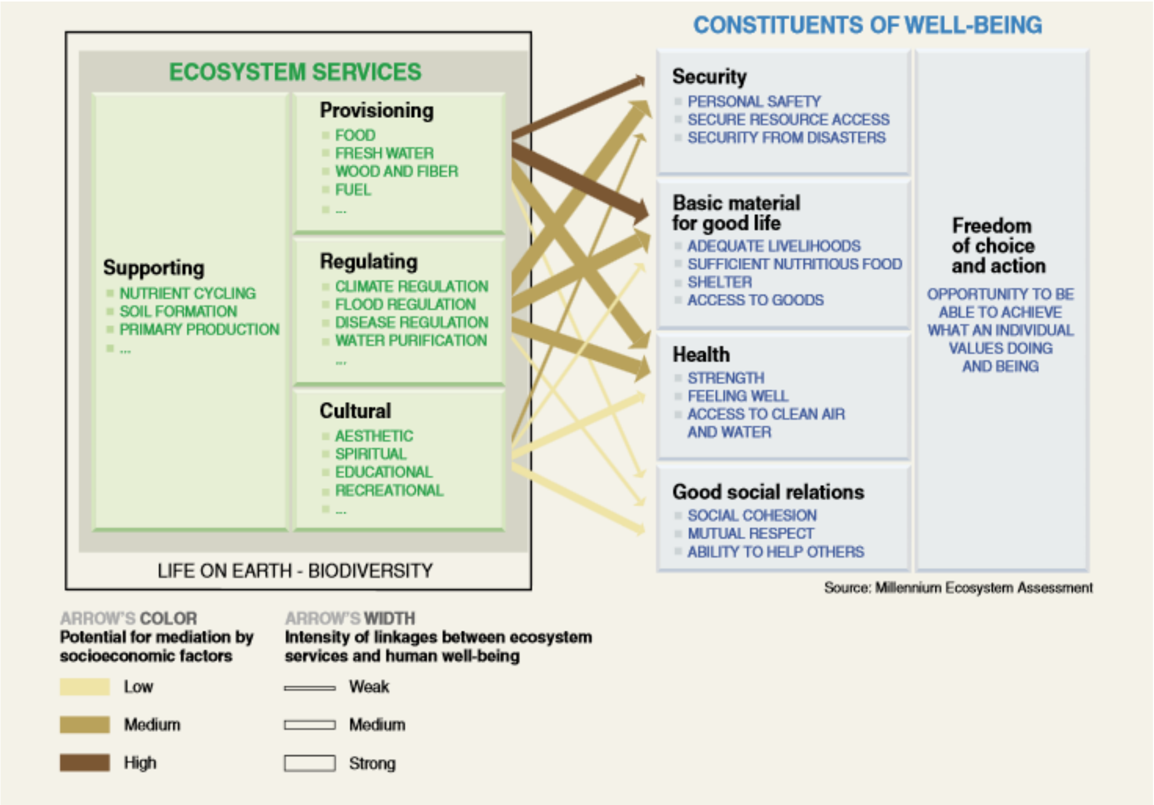

Figure A2.9. Linkages between ecosystem services and human well-being

Source: Millennium Ecosystem Assessment (2005) *Ecosystems and human well-being: current state and trends, Volume 1*. Island Press: Washington DC. Available at: http://www.unep.org/maweb/en/Condition.aspx [Accessed 22 July 2014]
Figure A, page vi. Reproduced with permission from World Resources Institute.

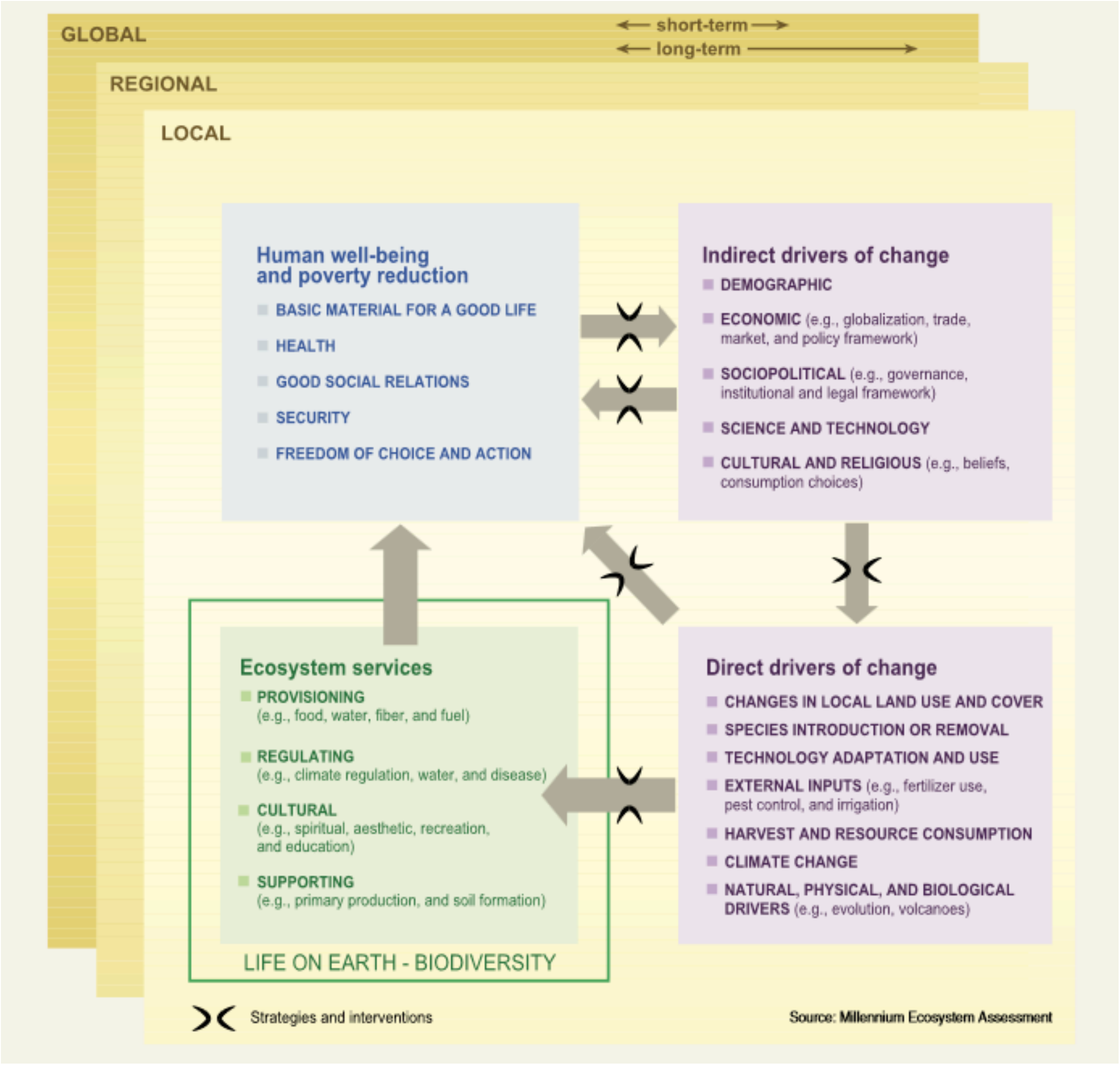

Figure A2.10. The Millennium Ecosystem Assessment Conceptual Framework: interactions between biodiversity, ecosystem services, human well-being, and drivers of change.

Source: Millennium Ecosystem Assessment (2005) *Ecosystems and human well-being: current state and trends, Volume 1*. Island Press: Washington DC. Available at: http://www.unep.org/maweb/en/Condition.aspx [Accessed 22 July 2014]
Figure B, page vii. Reproduced with permission from World Resources Institute.

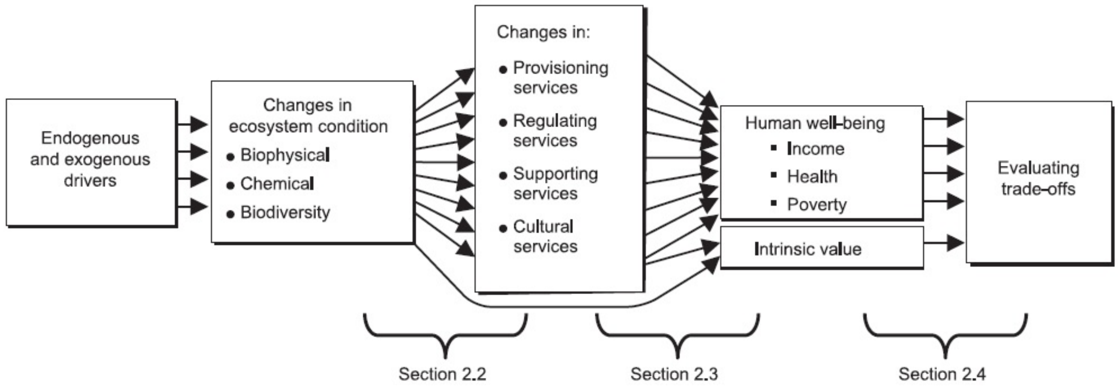

Figure A2.11. Different stages of assessment for linking ecosystem condition with human well-being. Section 2.2 refers to assessing ecosystem condition and trends, 2.3 refers to assessing the value of ecosystem services for human well-being, and 2.4 refers evaluation of trade-offs in ES**.**

Source: Millennium Ecosystem Assessment (2005) *Ecosystems and human well-being: current state and trends, Volume 1*. Island Press: Washington DC. Available at: http://www.unep.org/maweb/en/Condition.aspx [Accessed 22 July 2014]
Figure 2.1, page 40. Reproduced with permission from World Resources Institute.

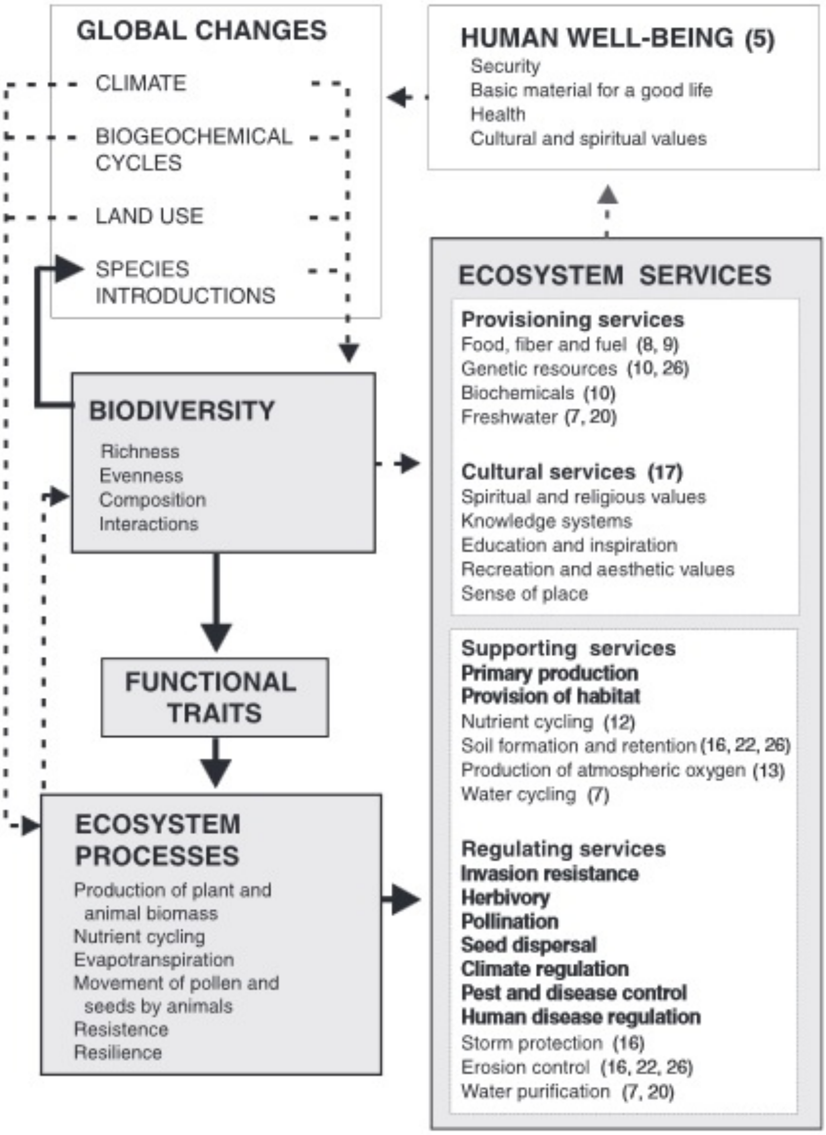

Figure A2.12 Biodiversity as response variable affected by global change drivers and as factor modifying ecosystem processes and services and human well-being.

Source: Millennium Ecosystem Assessment (2005) *Ecosystems and human well-being: current state and trends, Volume 1*. Island Press: Washington DC. Available at: http://www.unep.org/maweb/en/Condition.aspx [Accessed 22 July 2014]
Figure 11.1 page 300. Reproduced with permission from World Resources Institute.

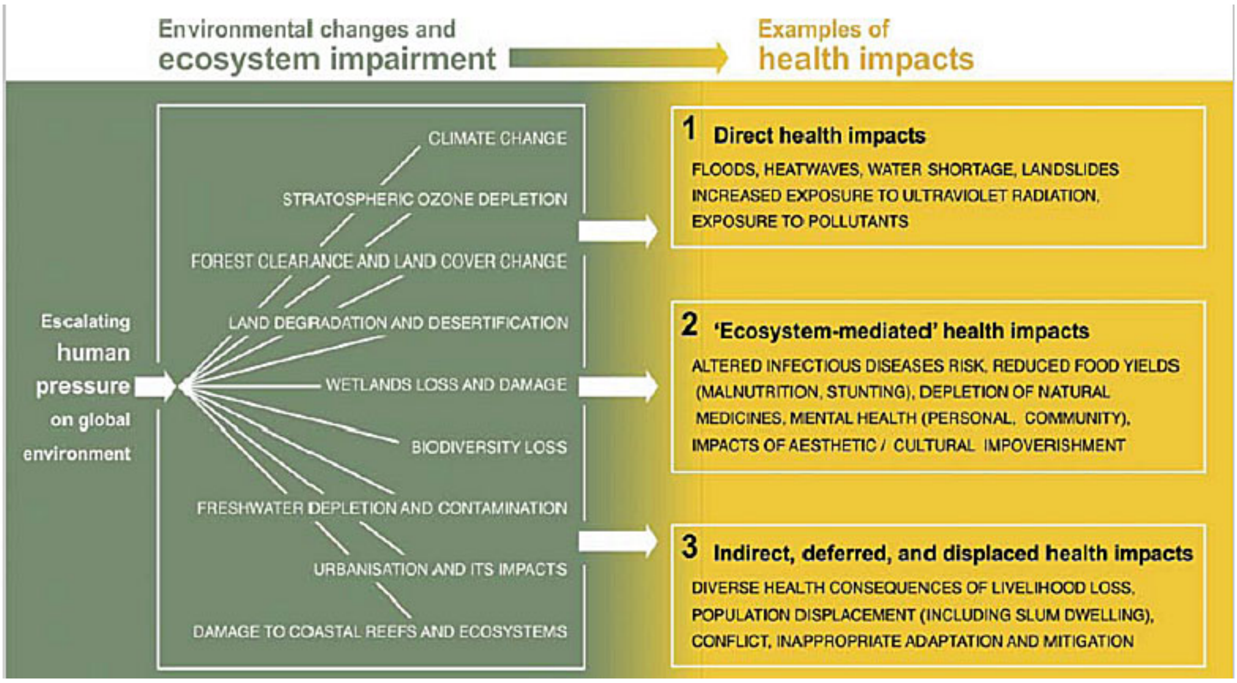

Figure A2.13. Harmful effects of ecosystem change on human health

Source: Millennium Ecosystem Assessment (2005) *Ecosystems and Human Well-being: Health Synthesis.* Island Press: Washington DC. Available at: http://www.millenniumassessment.org/documents/document.357.aspx.pdf [Accessed 11 March 2014]
Figure SDM1, page 1. Reproduced with permission from World Health Organization.

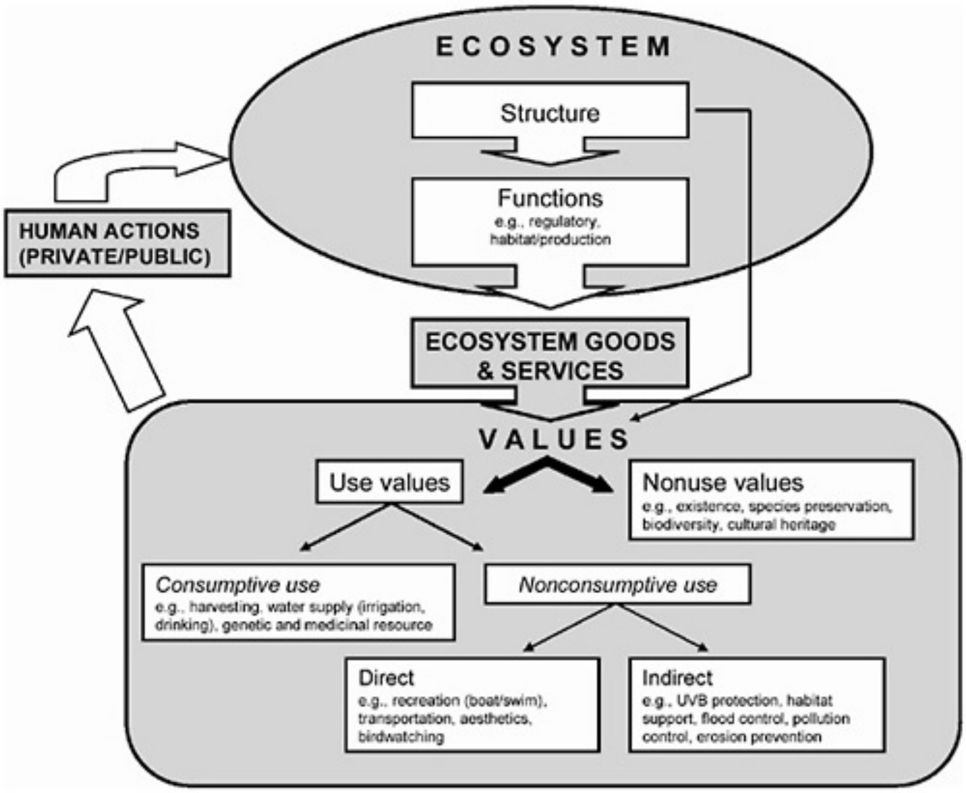

Figure A2.14 Connections between ecosystem structure and function, services, policies, and values.

Source: National Research Council (2005) *Valuing Ecosystem Services: Toward Better Environmental Decision Making.* National Academies Press: Washington, D.C., USA Figure 7.1, page 241 (Chapter 7, Ecosystem Valuation: Synthesis and Future Directions). Reproduced with permission from the National Academy of Sciences

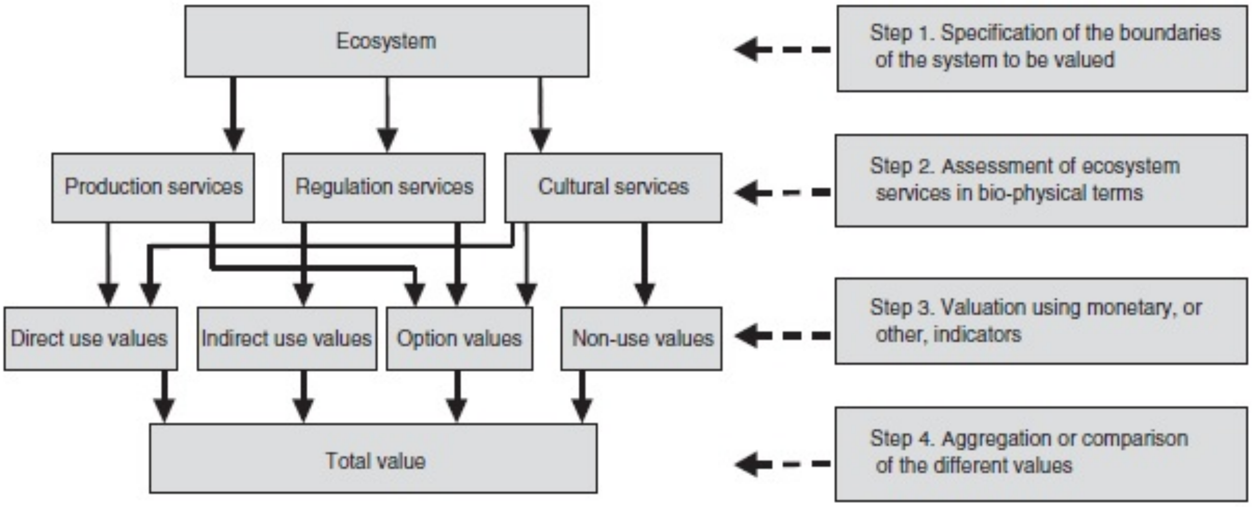

Figure A2.15 The ecosystem valuation framework. The solid arrows represent the most important links between the elements of the framework. The dashed arrows indicate the four principal steps in the valuation of ecosystem services.

Source: Hein, L., van Koppen, K., de Groot, R.S. & van Ierland, E.C. (2006) Spatial scales, stakeholders and the valuation of ecosystem services. *Ecological Economics,* 57: 209– 228 Figure 1, page 211. Reproduced with permission from Elsevier.

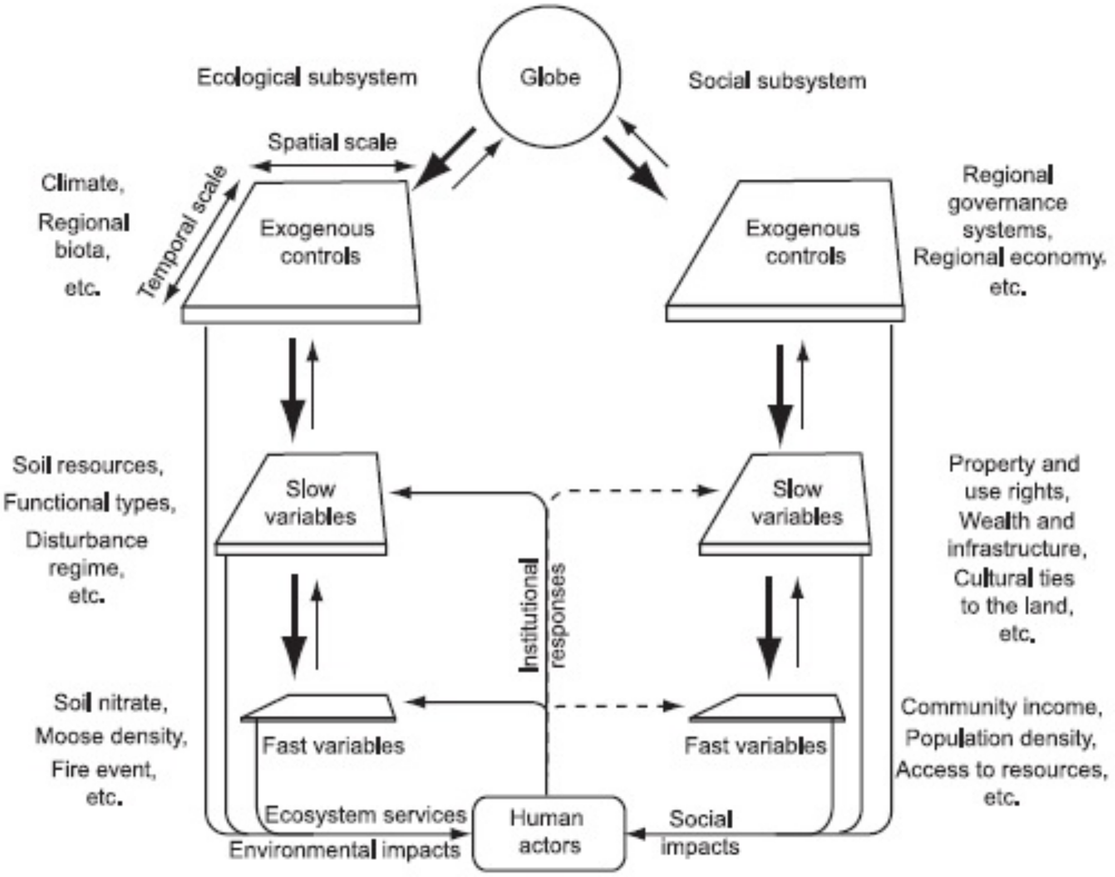

Figure A2.16. Diagram of a social– ecological system comprising an ecological subsystem and a social subsystem, each with a spectrum of controls that operate across a range of temporal and spatial scales

Source: Chapin, F.S., Lovecraft, A.L., Zavaleta, E.S., Nelson, J., Robards, M.D., Kofinas, G.P. et al. (2006) Policy strategies to address sustainability of Alaskan boreal forests in response to a directionally changing climate. *Proceedings of the National Academy of Sciences of the United States of America,* 103:16637-16643
Figure 1, page 16638. Reproduced with permission from the National Academy of Sciences.

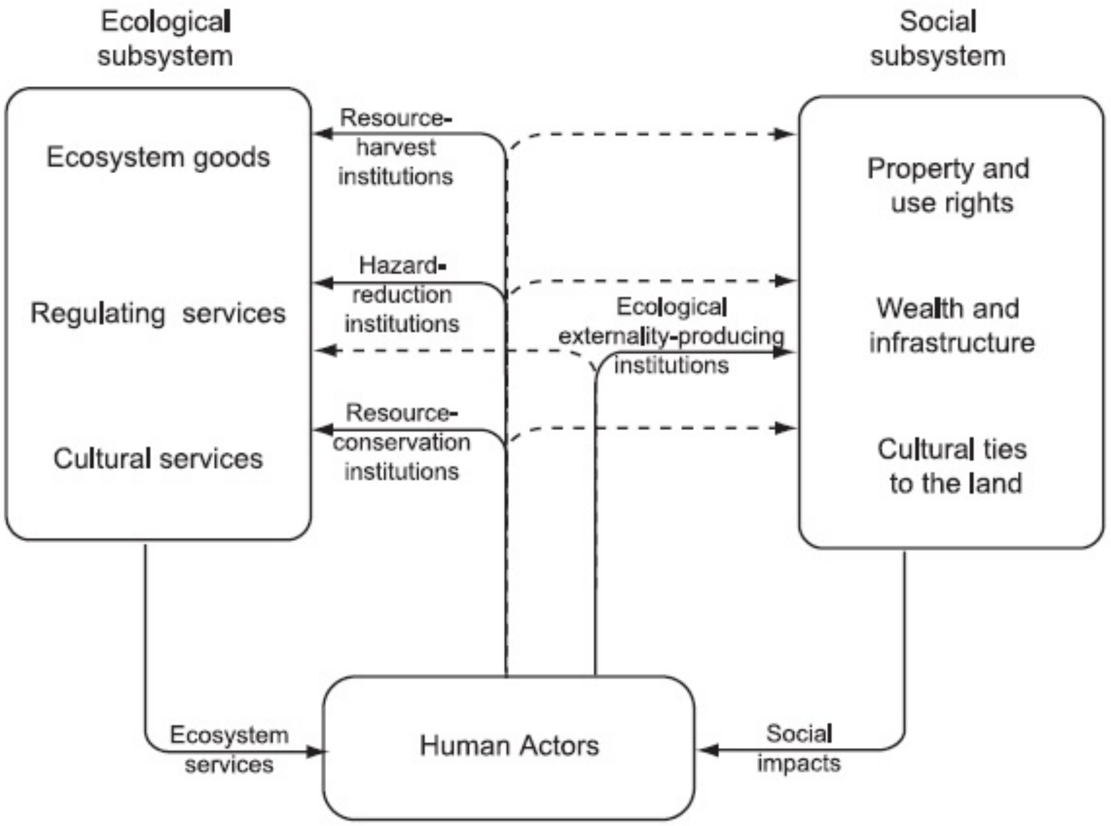

Figure A2.17 Ecological institutions that influence ecosystem services (solid lines represent direct effects and dashed lines represent indirect effects)

Source: Chapin, F.S., Lovecraft, A.L., Zavaleta, E.S., Nelson, J., Robards, M.D., Kofinas, G.P. et al. (2006) Policy strategies to address sustainability of Alaskan boreal forests in response to a directionally changing climate. *Proceedings of the National Academy of Sciences of the United States of America,* 103:16637-16643
Figure 2, page 16639. Reproduced with permission from the National Academy of Sciences.

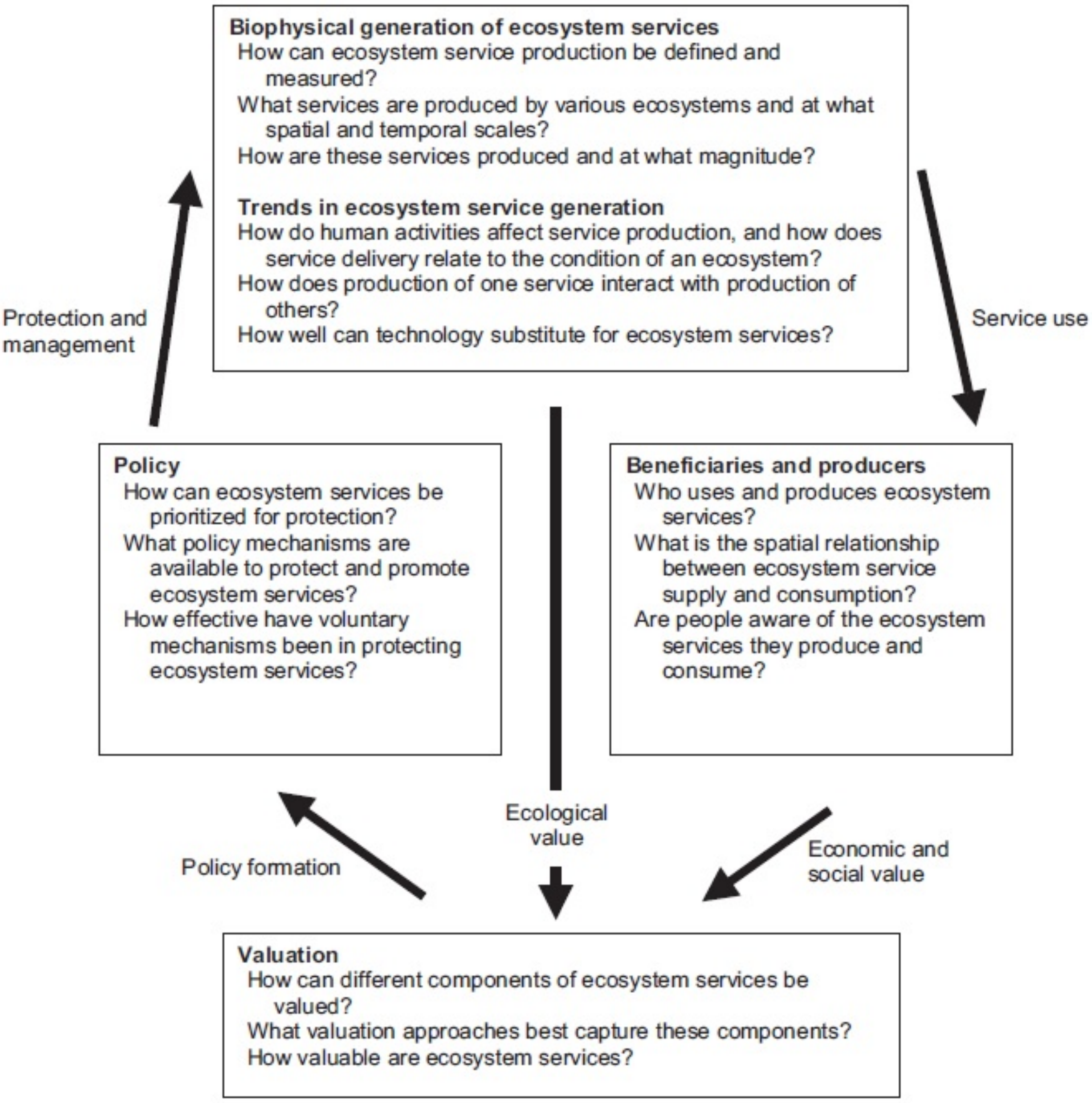

Figure A2.18 Policy-relevant questions for understanding, assessing, and managing ecosystem services

Source: Brauman, K.A. Daily, G.C., Duarte, T.K. & Mooney, H.A. (2007). The nature and value of ecosystem services: an overview highlighting hydrologic services. *Annual Review of Environment and Resources,* 32:67–98
Figure 2, page 71. Reproduced with permission from Annual Reviews

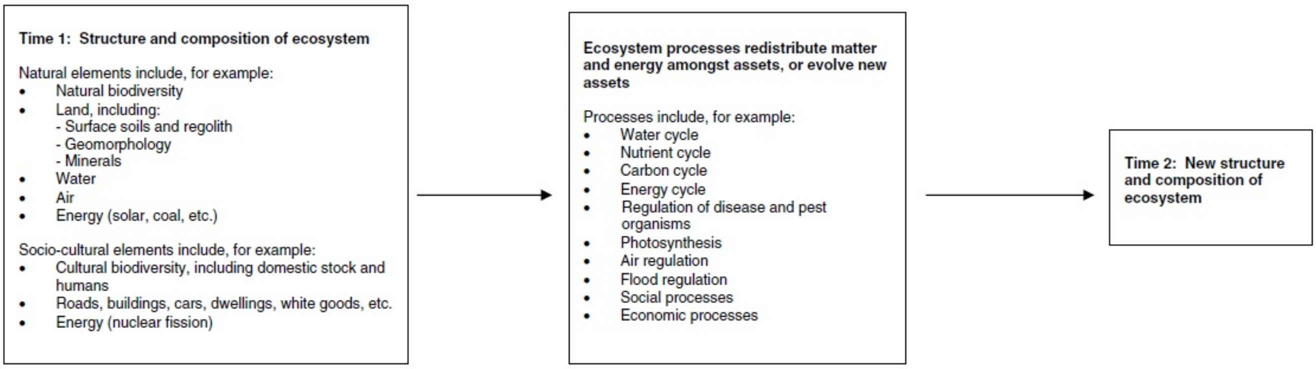

Figure A2.19. Description of the relationships among ecosystem elements and processes. (For simplicity it is assumed that natural assets are the sum of biotic and abiotic ecosystem elements, rather than a sub-set only)

Source: Wallace, K.J. (2007) Classification of ecosystem services: Problems and solutions. *Biological Conservation*, 139: 235-246
Figure 1, page 237. Reproduced with permission from Elsevier

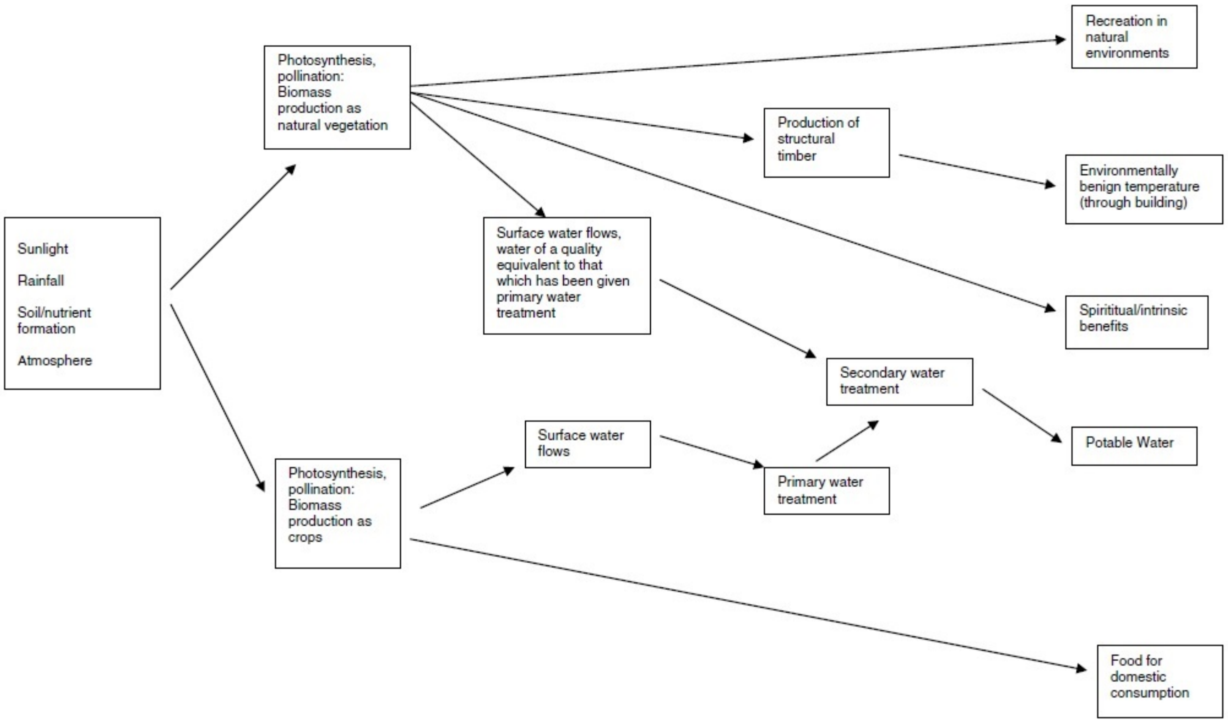

Figure A2.20 Simplified scheme of the ecosystem pathways for delivering five ecosystem services (right side)

Source: Wallace, K.J. (2007) Classification of ecosystem services: Problems and solutions. *Biological Conservation*, 139: 235-246
Figure 2, page 238. Reproduced with permission from Elsevier

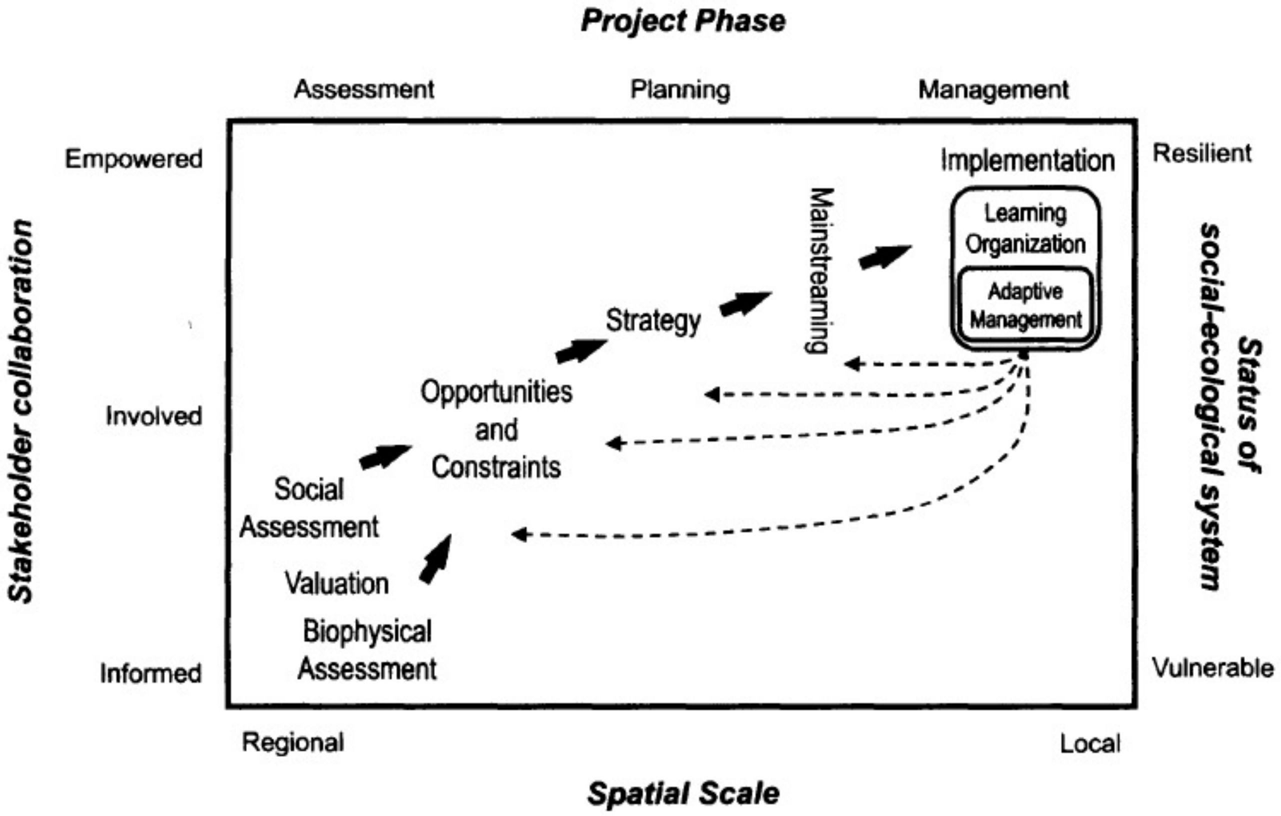

Figure A2.21 An operational model for implementing the safeguarding of ecosystem services.

Source: Cowling, R.M., Egoh, B., Knight, A.T., O'Farrell, P.J., Reyers, B., Rouget, M. et al (2008) An operational model for mainstreaming ecosystem services for implementation. *Proceedings of the National Academy of Sciences of the United States of America*, 105: 9483-9488
Figure 1, page 9484. Reproduced with permission from the National Academy of Sciences

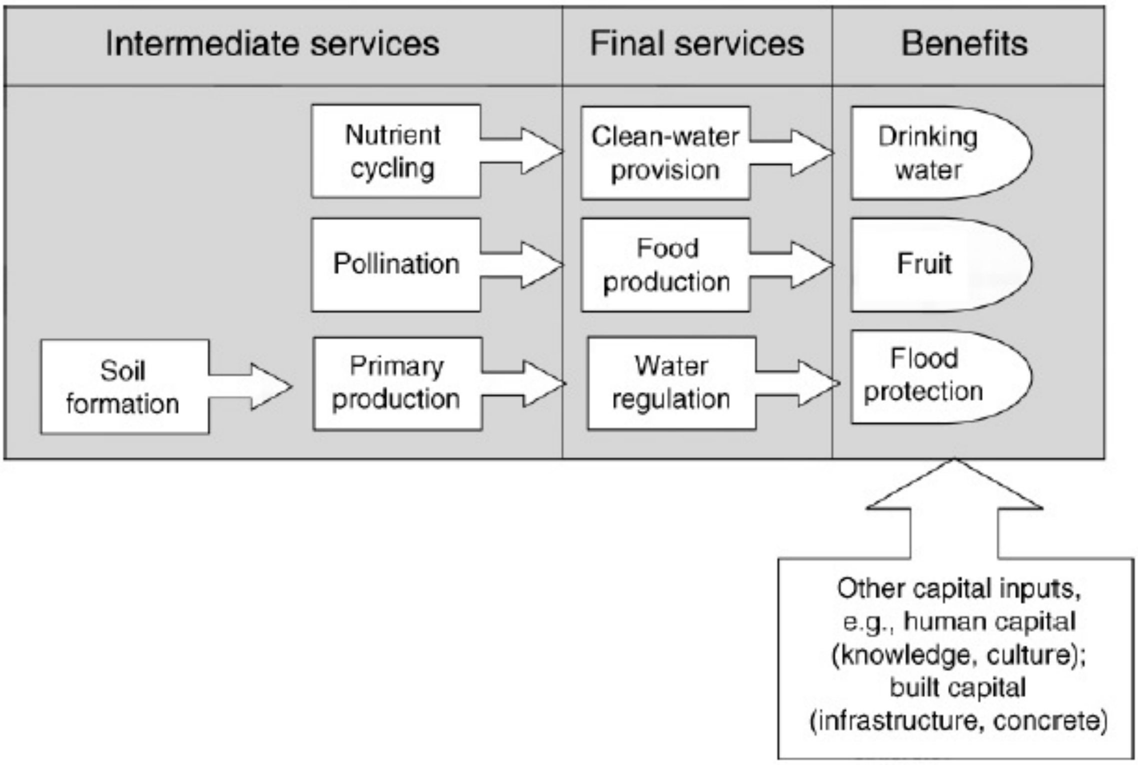

Figure A2.22. Stylized relationships among representative intermediate services, final services, and benefits (the complexity and interactions are simplified for heuristic value).

Source: Fisher, B., Turner, K., Zylstra, M., Brouwer,R., de Groot, R., Farber, S. et al. (2008) Ecosystem services and economic theory: integration for policy-relevant research. *Ecological Applications,* 18(8): 2050–2067
Figure 1, page 2052. Reproduced with permission from Ecological Society of America

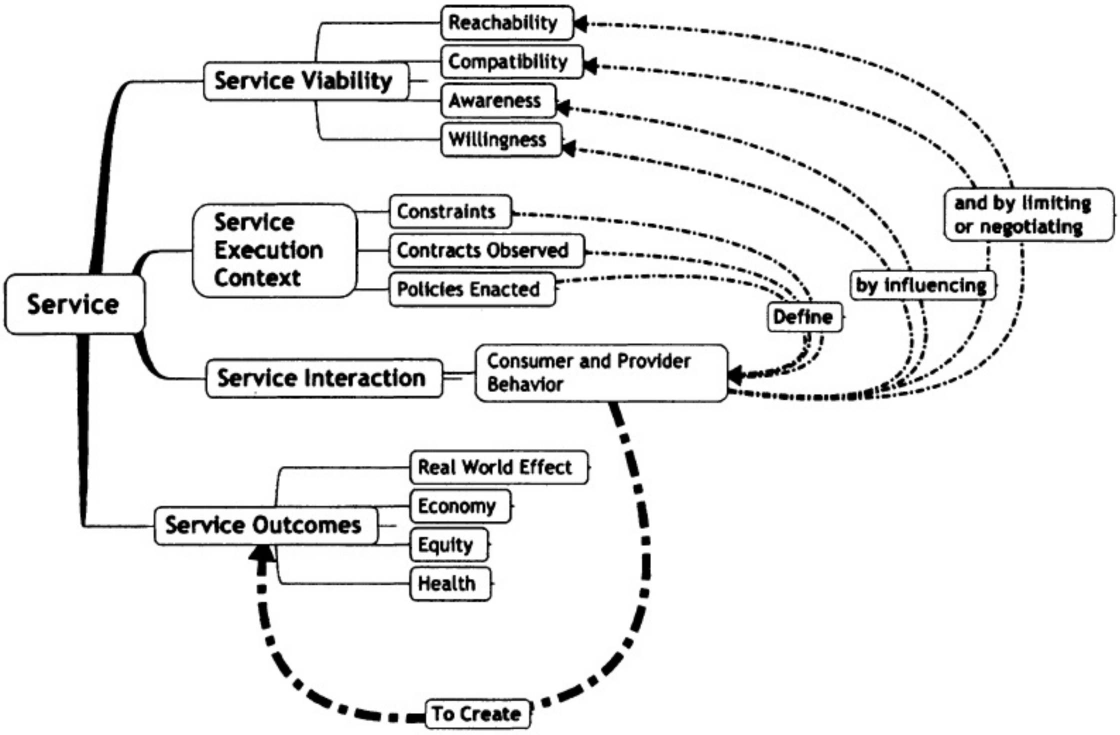

Figure A2.23 Factors that guide agency and path dependence in ecosystem service use.

Source: Loring, P.A., Chapin III, F.S. & Gerlach, S.C. (2008) The Services-oriented architecture: Ecosystem services as a framework for diagnosing change in social ecological systems. *Ecosystems,* 11: 478-489
Figure 2, page 481. Reproduced with permission from Springer-Verlag.

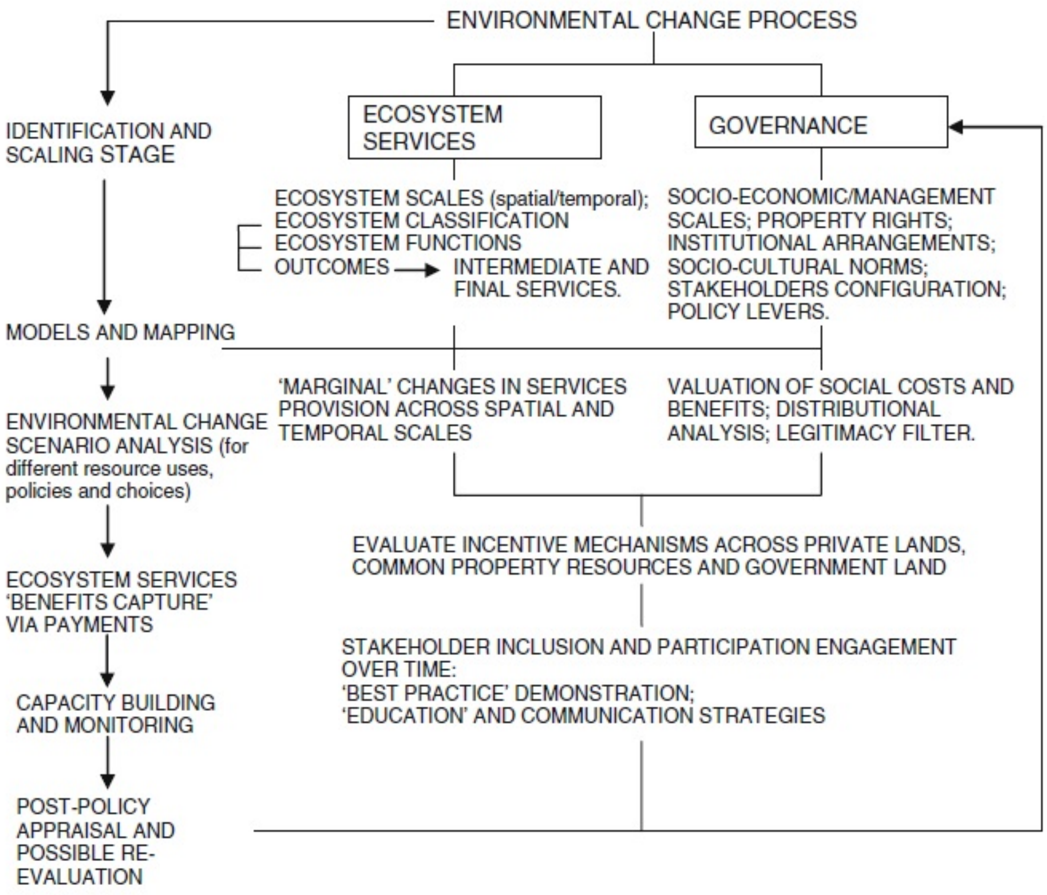

Figure A2.24 Ecosystem Services Framework- ecosystem services-based decision support process.

Source: Turner, R.K & Daily, G.C. (2008) The ecosystem services framework and natural capital conservation. *Environmental and Resource Economics,* 39:25–35
Figure 1, page 27. Reproduced with permission from Ecological Society of America

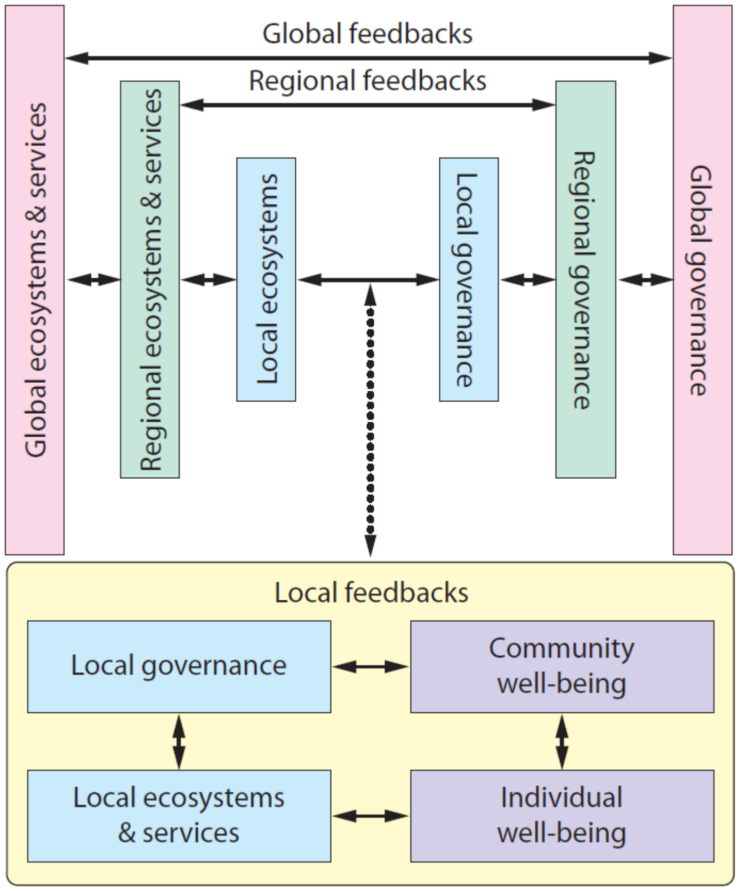

Figure A2.25 Multi-scale linkages of governance and ecosystem services. Governance and ecosystem services are interlinked at multiple scales, in ways that may or may not be effective for building or maintaining ecosystem services and human well-being

Source: Carpenter, R.S., Mooney, H.A., Agard, J., Capistrano, D., DeFries, R.S., Diaz, S. et al. (2009) Science for managing ecosystem services: Beyond the Millennium Ecosystem Assessment. *Proceedings of the National Academy of Sciences of the United States of America*, 106: 1305–1312.
Figure 4, page 1310. Reproduced with permission from the National Academy of Sciences

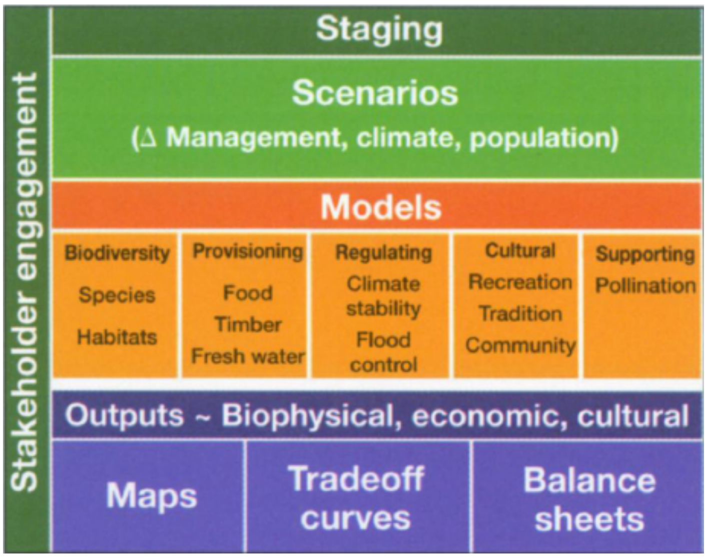

Figure A2.26 An iterative process for integrating ecosystem services into decisions.

Source: Daily, G.C., Polasky, S., Goldstein, J., Kareiva, P.M., Mooney, H.A., Pejchar, L. et al. (2009) The role of ecosystem services in conservation and resource management. *Frontiers in Ecology and the Environment*, 7:21-28
Figure 1, page 22. Reproduced with permission from the Ecological Society of America.

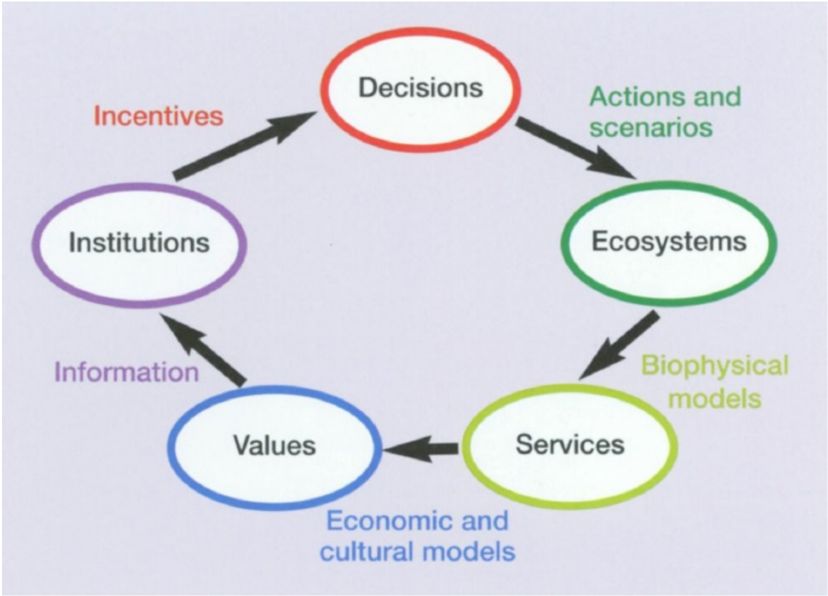

Figure A2.27 A framework showing how ecosystem services can be integrated into decision making. Any two ovals can be linked in any direction; the simplest version is presented here.

Source: Daily, G.C., Polasky, S., Goldstein, J., Kareiva, P.M., Mooney, H.A., Pejchar, L. et al. (2009) The role of ecosystem services in conservation and resource management. *Frontiers in Ecology and the Environment*, 7:21-28
Figure 2, page 23. Reproduced with permission from the Ecological Society of America.

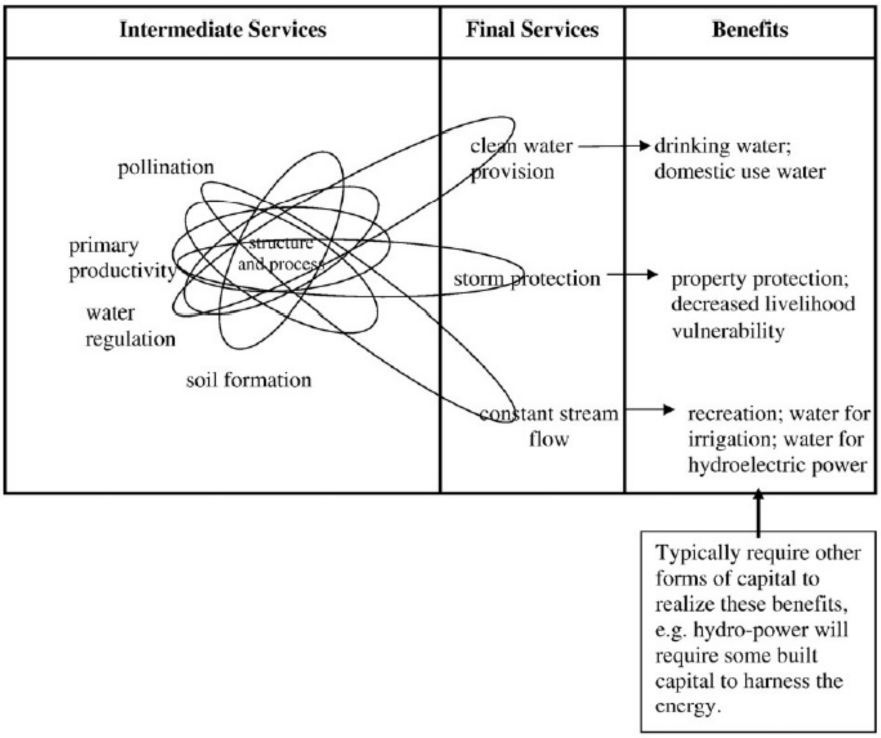

Figure A2.28 Conceptual relationship between intermediate and final services, also showing how joint products (benefits) can stem from individual services.

Source: Fisher, B., Turner, R.K. & Morling, P. (2009) Defining and classifying ecosystem services for decision making. *Ecological Economics,* 68:643-653
Figure 3, page 646. Reproduced with permission from Elsevier

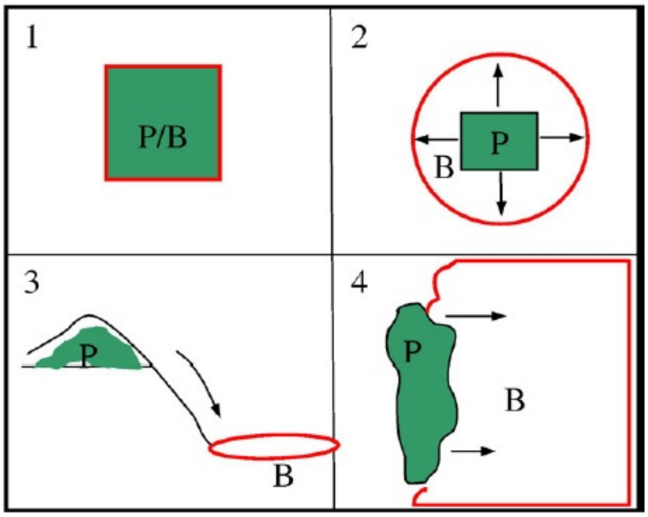

Figure A2.29. Possible spatial relationships between service production areas (P) and service benefit areas (B) (see original for further explanation).

Source: Fisher, B., Turner, R.K. & Morling, P. (2009) Defining and classifying ecosystem services for decision making. *Ecological Economics,* 68:643-653
Figure 5, page 650. Reproduced with permission from Elsevier

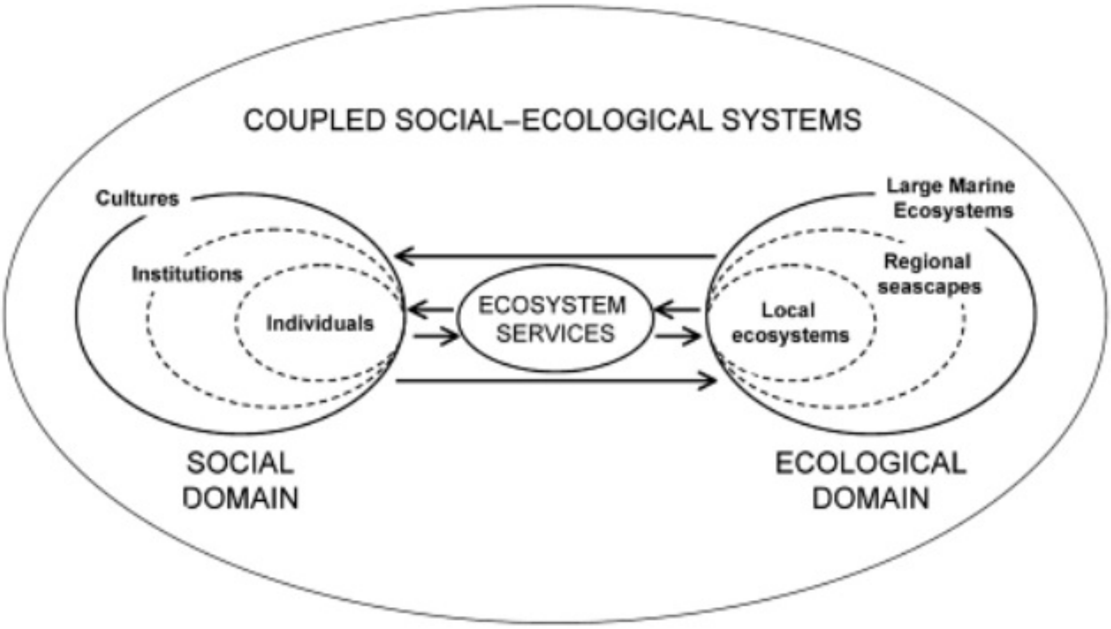

Figure A2.30 Dynamic human and ecological systems referred to as 'coupled socio-ecological systems'

Source: McLeod, K. & Leslie, H. (eds) (2009) *Ecosystem-Based Management for the Oceans*. Island Press: Washington, DC
Figure 1.1, page 5 (Chapter 1: Why Ecosystem-Based Management? K. McLeod & H. Leslie). Reproduced with permission from Island Press.

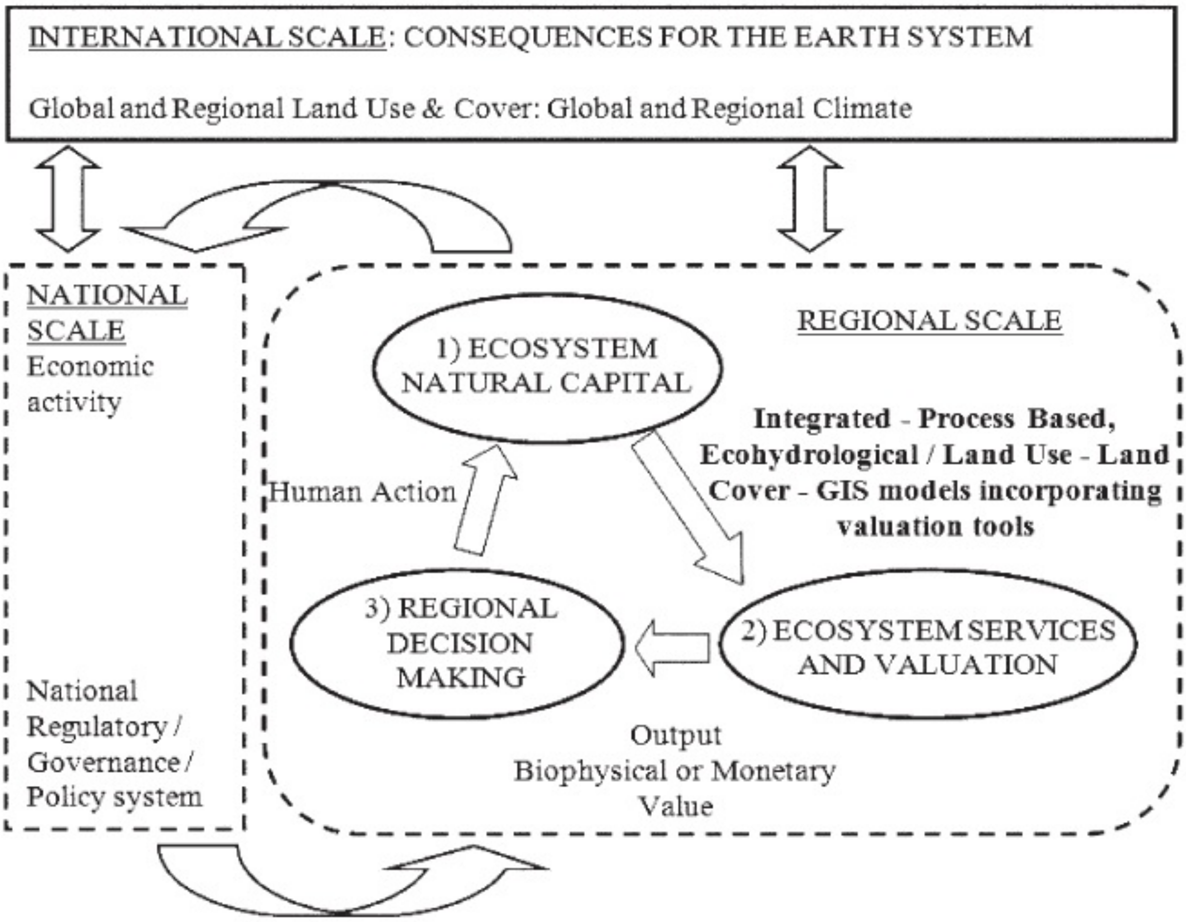

Figure A2.31 A schematic diagram indicating an integrated process/economic modelling framework.

Source: Robinson, D.A., Libron, I. & Vereecken, H. (2009) On the definition of the natural capital of soils: a framework for description, evaluation, and monitoring. *Soil Science Society of America Journal,* 73: 1904–1911
Figure 2, page 1908. Reproduced with permission from Alliance of Crop, Soil, and Environmental Science Societies.

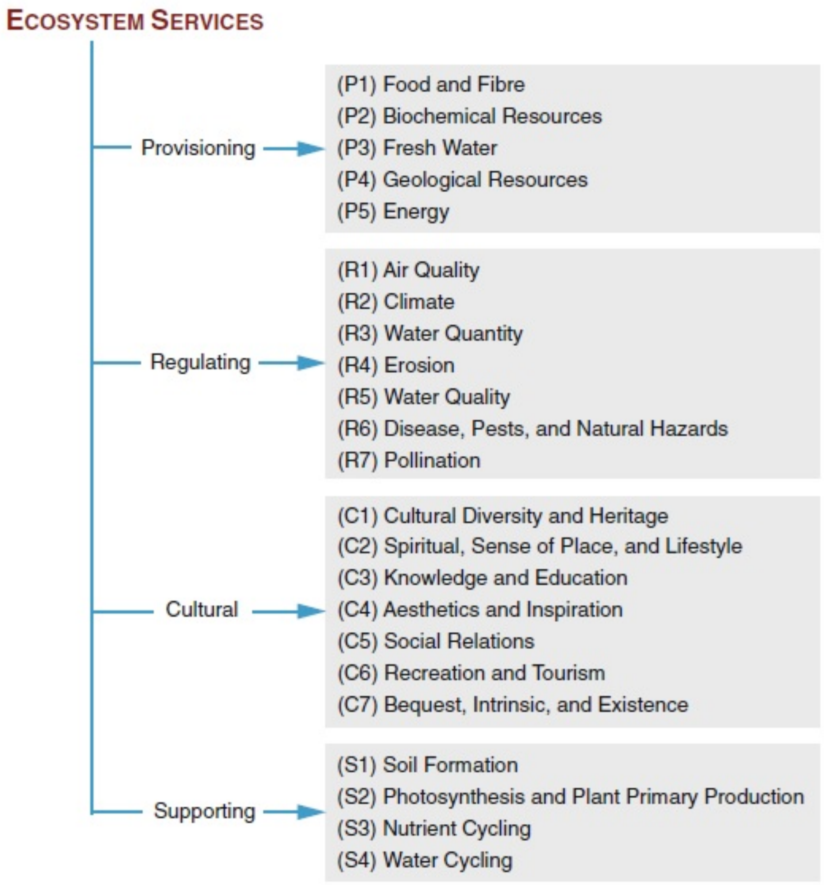

Figure A2.32. Ecosystem service-based goals hierarchy with individual services (right) grouped into four ecosystem services types.

Source: Bryan, B.A., Grandgirard, A. & Ward, J.R. (2010) Quantifying and exploring strategic regional priorities for managing natural capital and ecosystem services given multiple stakeholder perspectives. *Ecosystems*, 13:539–555
Figure 3, page 543. Reproduced with permission from Springer-Verlag.

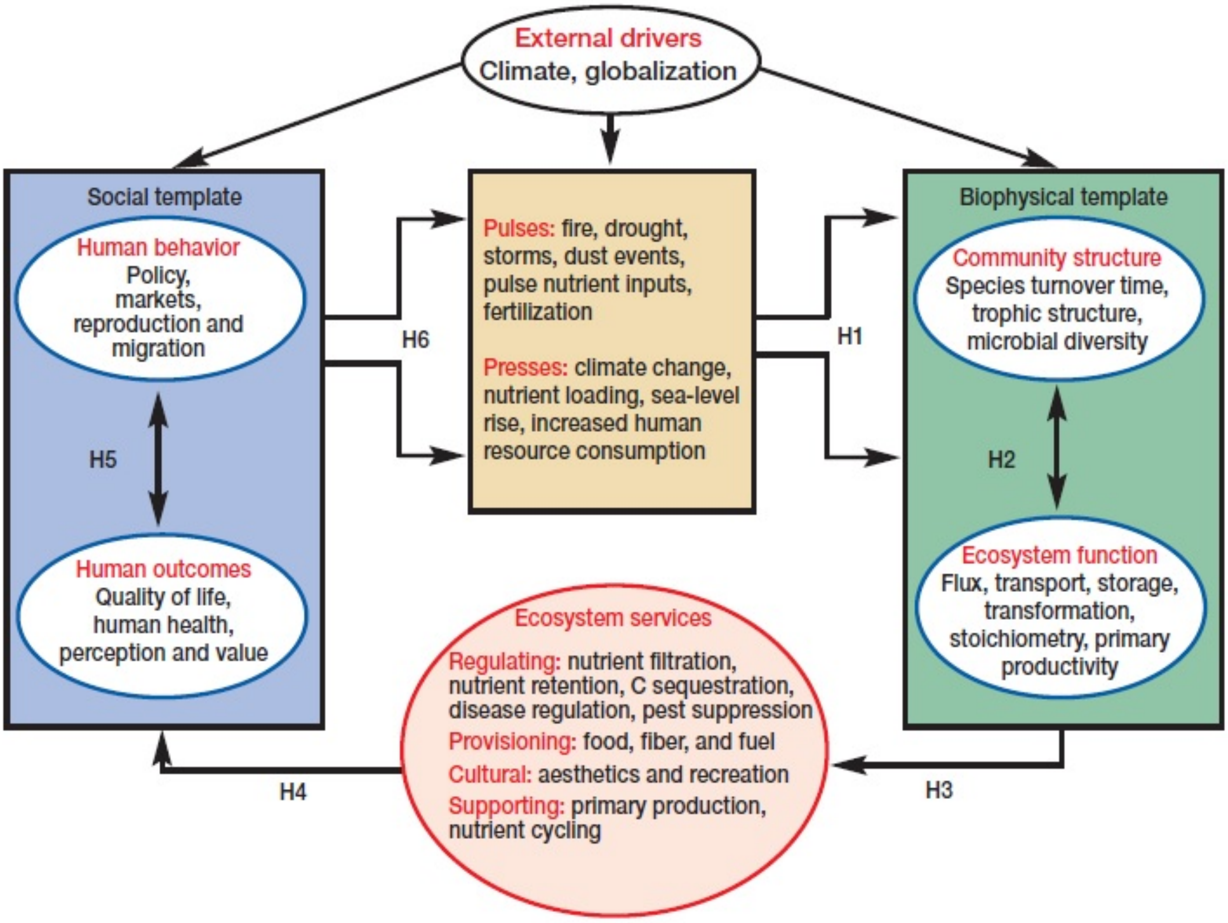

Figure A2.33 The Press-Pulse Dynamics framework provides the basis for long-term, integrated, social–ecological research. The right-hand side represents the domain of traditional ecological research; the left-hand side represents human dimensions of environmental change; the two are linked by ecosystem services and by pulse and press events influenced or caused by human behaviour (bottom and top, respectively).

Source: Collins, S.L., Carpenter, S.R., Swinton, S.M., Orenstein, D.E., Childers, D.L., Gragson, T.L. et al (2010) An integrated conceptual framework for long-term social–ecological research. *Frontiers in Ecology and the Environment,* 9: 351–357
Figure 1, page 353 Reproduced with permission from the Ecological Society of America

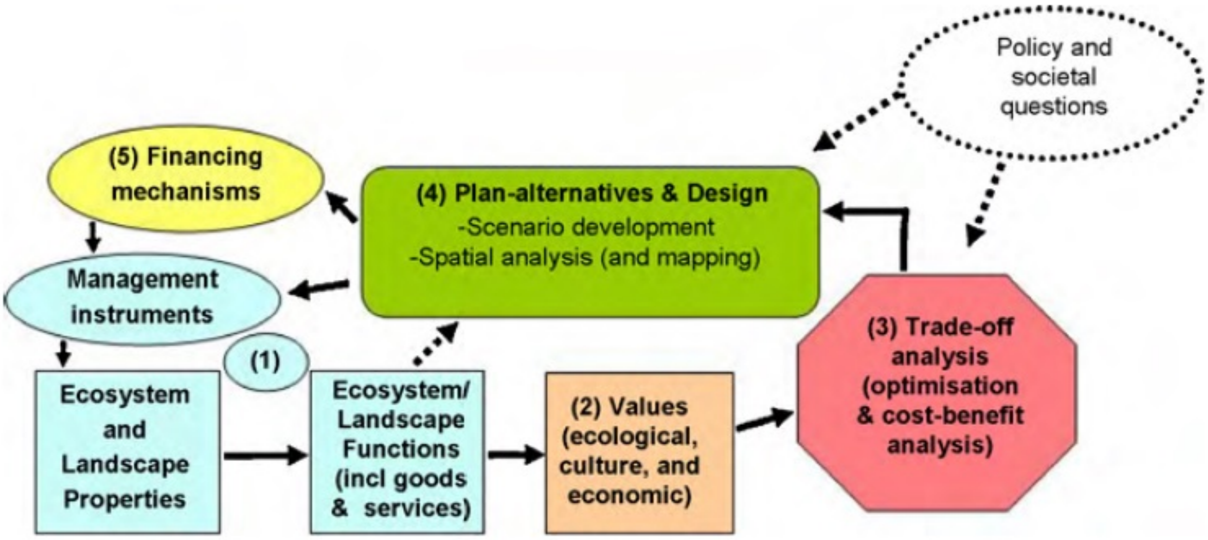

Figure A2.34 Framework for integrated assessment of ecosystem and landscape services.

Source: de Groot, R.S., Alkemade, R., Braat, L., Hein, L. & Willemen, L. (2010a). Challenges in integrating the concept of ecosystem services and values in landscape planning, management and decision making. *Ecological Complexity*, 7: 260–272
Figure 1, page 262. Reproduced with permission from Elsevier

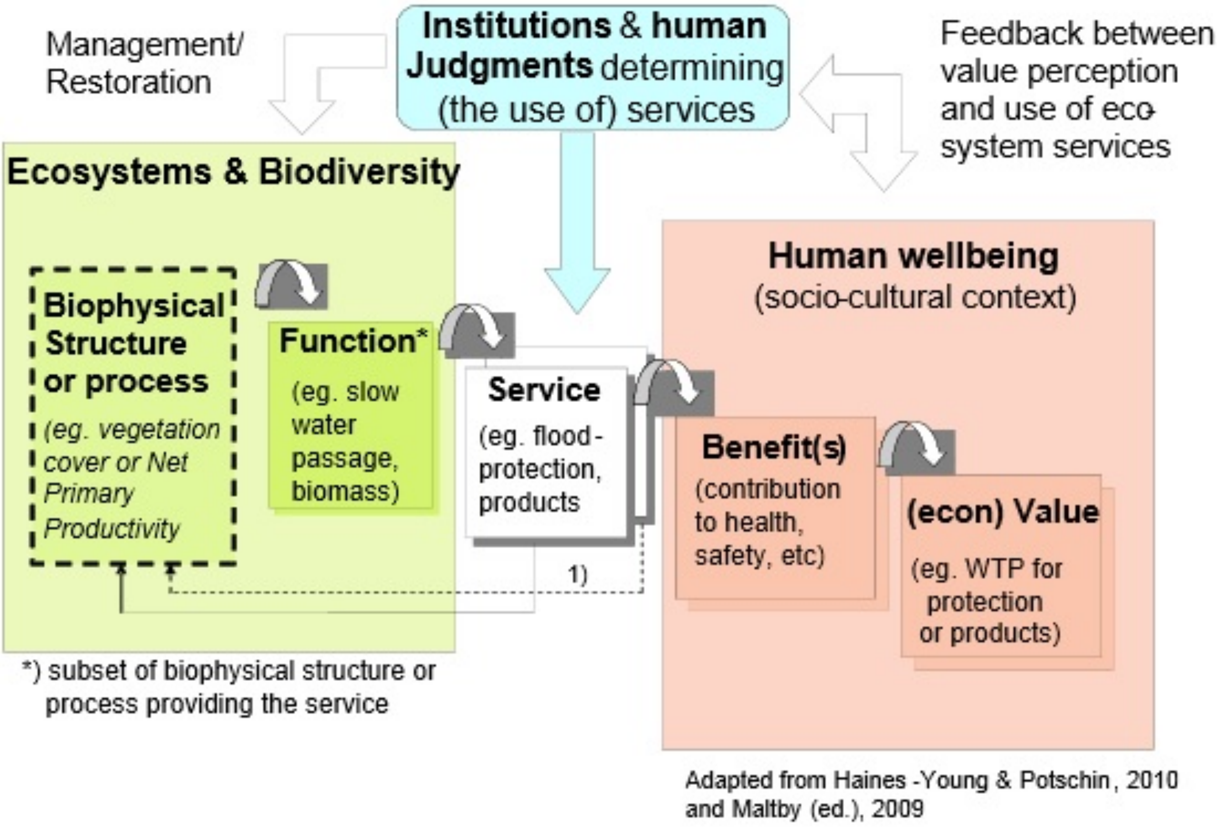

Figure A2.35 The pathway from ecosystem structure and processes to human well-being**.**

Source: de Groot, R., Fisher, B., Christie, M., Aronson, J., Braat, L., Gowdy J, et al. (2010) *Integrating the ecological and economic dimensions in biodiversity and ecosystem service valuation*. In: Kumar, P. (ed.) The Economics of Ecosystems and Biodiversity: Ecological and Economic Foundations, London: Earthscan, 9–40
Figure 4, page 11. Reproduced with permission from Taylor & Francis

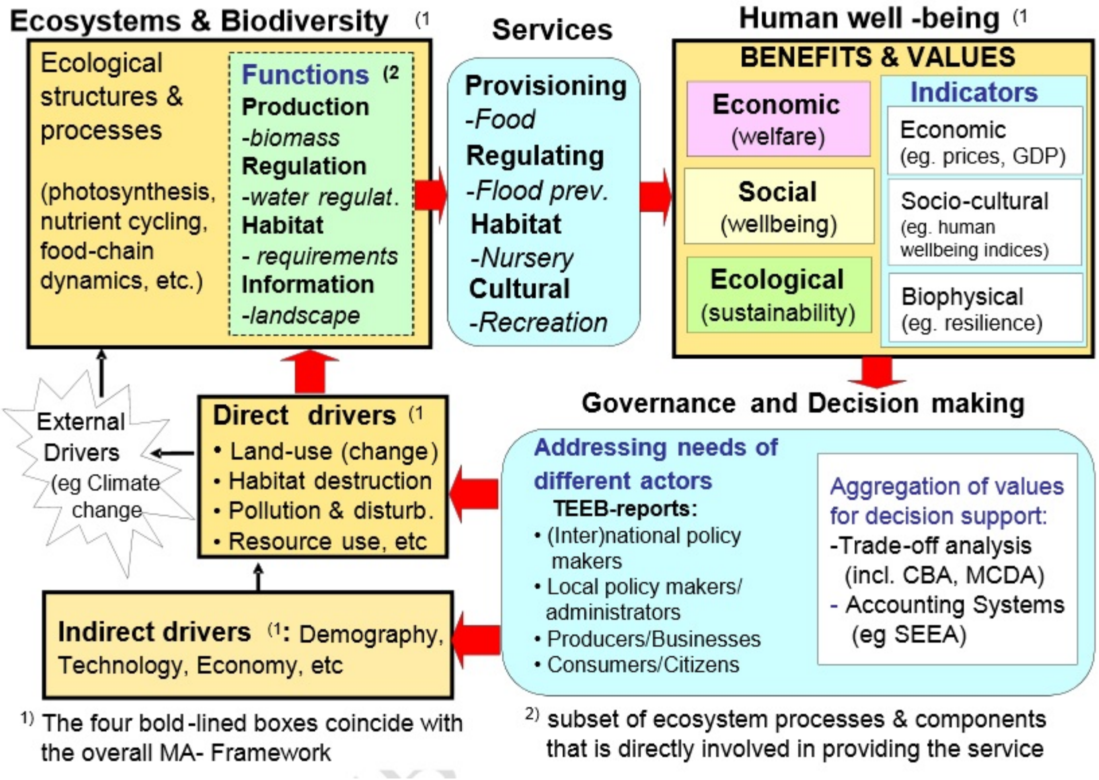

Figure A2.36 Conceptual framework for linking ecosystems and human well-being

Source: de Groot, R., Fisher, B., Christie, M., Aronson, J., Braat, L., Gowdy J, et al. (2010) *Integrating the ecological and economic dimensions in biodiversity and ecosystem service valuation*. In: Kumar, P. (ed.) The Economics of Ecosystems and Biodiversity: Ecological and Economic Foundations, London: Earthscan, 9–40
Figure 5, page 15. Reproduced with permission from Taylor & Francis

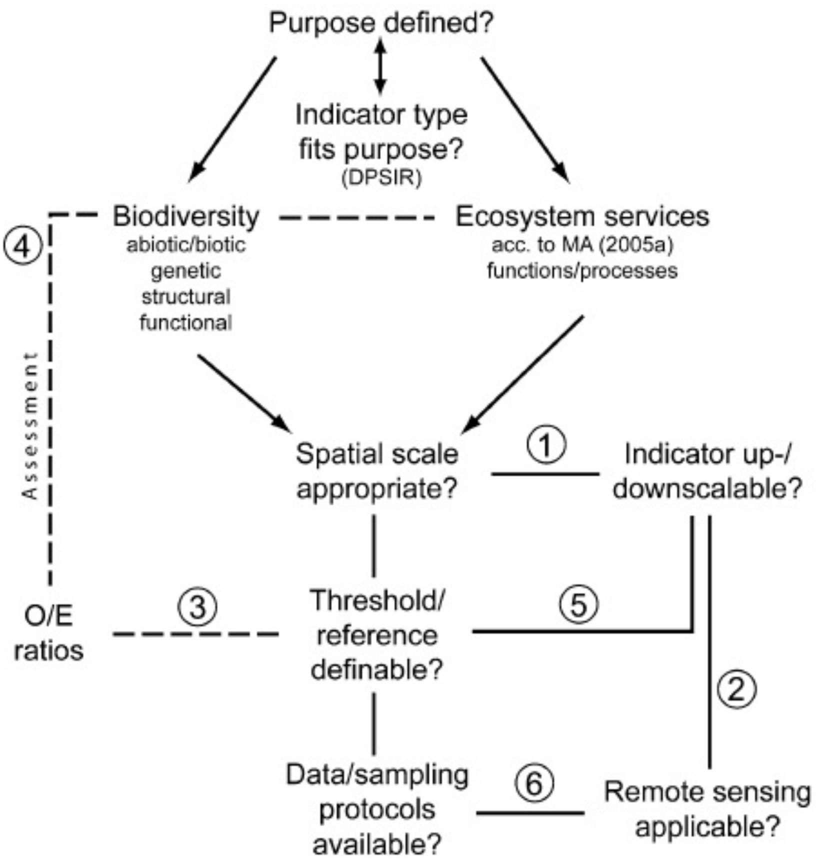

Figure A2.37 Schema showing the interdependence of the indicator suitability criteria.

Source: Feld, C.K., Sousa, J.P., Martins da Silva, P. & Dawson, T.P. (2010) Indicators for biodiversity and ecosystem services: towards an improved framework for ecosystems assessment. *Biodiversity & Conservation,* 19:2895–2919
Figure 1, page 2901. Reproduced with permission from Springer Netherlands.

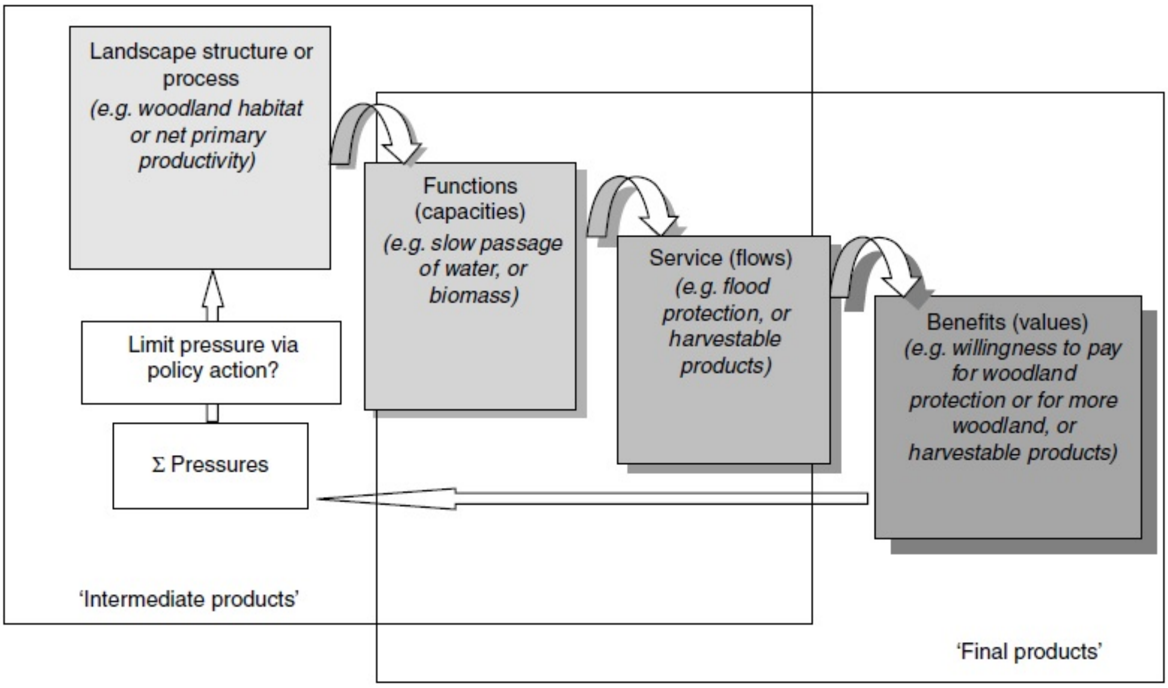

Figure A2.38 The relationship between biodiversity, ecosystem function and human well-being.

Source: Haines-Young, R., & Potschin, M. (2010) *The links between biodiversity, ecosystem services and human well-being.* In: Raffaelli, D. and Frid, C. (eds) Ecosystem Ecology: A New Synthesis. BES Ecological Reviews Series, CUP. Cambridge: Cambridge University Press, 110–139
Figure 6.2, page 116. Reproduced with permission from Cambridge University Press

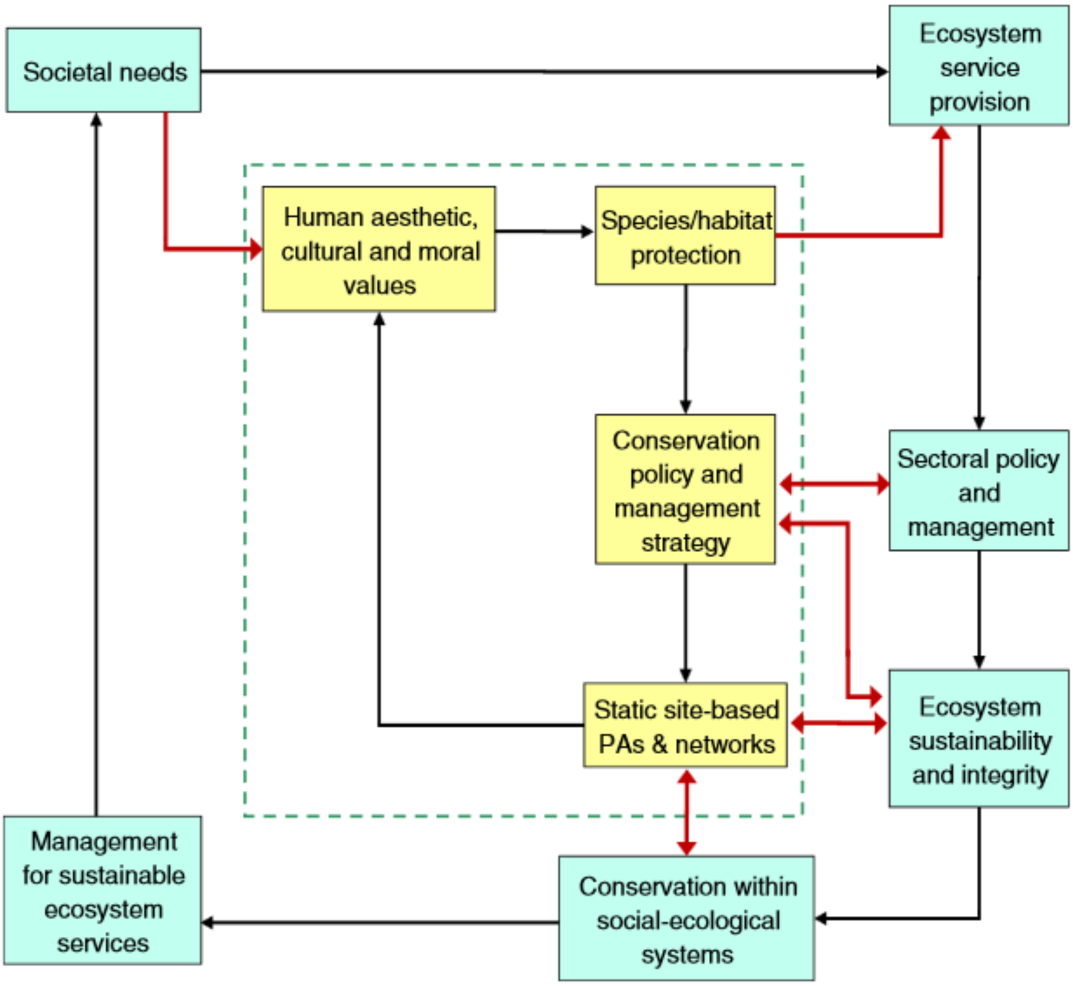

Figure A2.39 A framework for conservation in Europe integrating Ecosystem Services (ES) (Traditional conservation strategy is represented in the inner loop (within dashed line, PAs = protected areas) while wider societal needs and ES provision are depicted in the outer loop. The main links between the two loops are indicated by arrows).

Source: Haslett, J.R., Berry, P.M., Bela, G., Jongman, R.H.G., Pataki, G., Samways, M.J. & Zobel, M. (2010) Changing conservation strategies in Europe: a framework integrating ecosystem services and dynamics. *Biodiversity & Conservation*, 19:2963–2977
Figure 1, page 2969. Reproduced with permission from Springer Netherlands

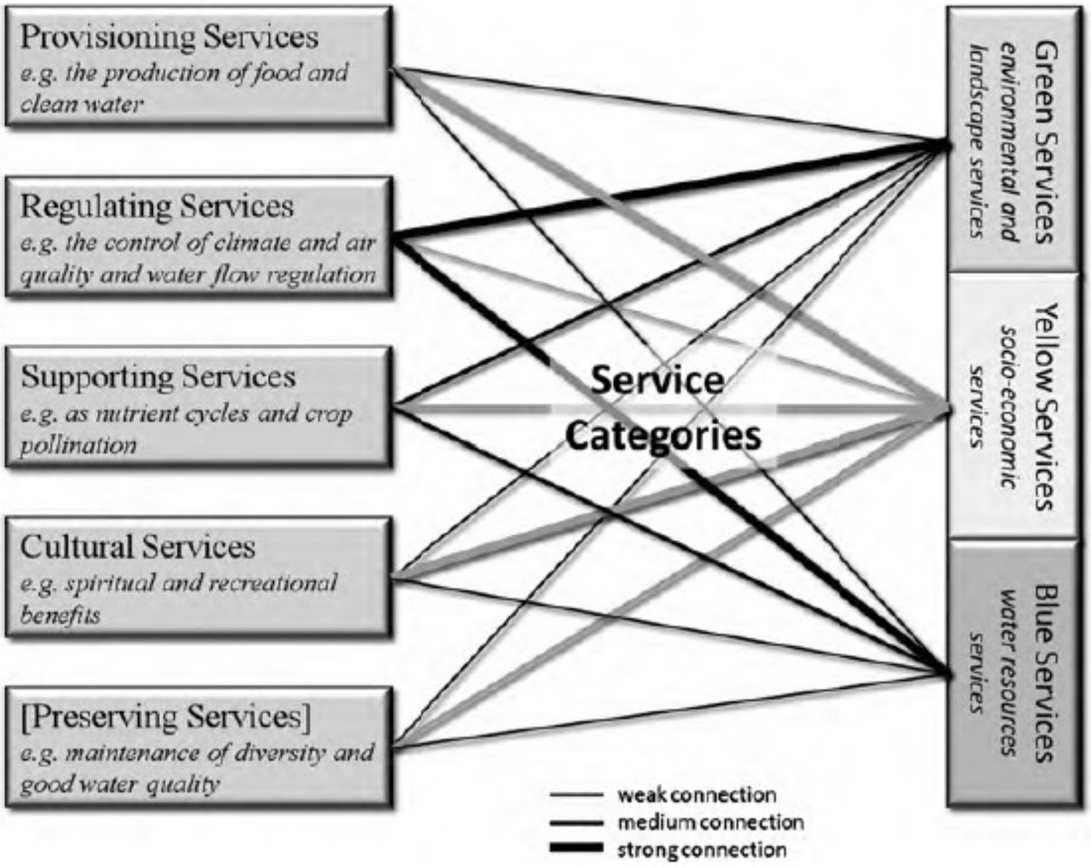

Figure A2.40 Ecosystem goods and services unitized into three service categories: green, blue, and yellow services

Source: Klug, H. & Jennewien, P. (2010) Spatial modelling of agrarian subsidy payments as an input for evaluating changes of ecosystem services. *Ecological Complexity*, 7:368–377 Figure 3, page 374. Reproduced with permission from Elsevier

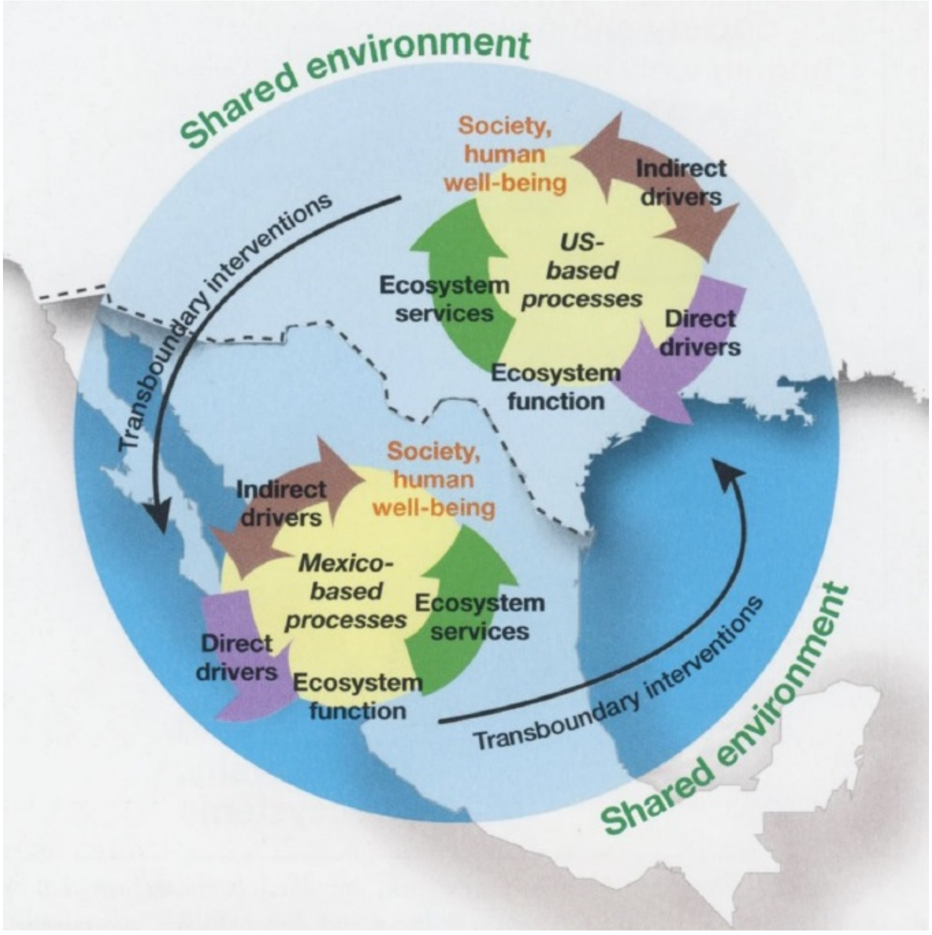

Figure A2.41 The MA (2003) framework adapted to explain transboundary ecosystem services shared by the US and Mexico.

Source: López-Hoffman, L., Varady, R.G., Flessa, K.W. & Balvanera, P. (2010) Ecosystem services across borders: a framework for transboundary conservation policy. *Frontiers in Ecology and the Environment*, 8:84-91
Figure 2, page 86. Reproduced with permission from the Ecological Society of America

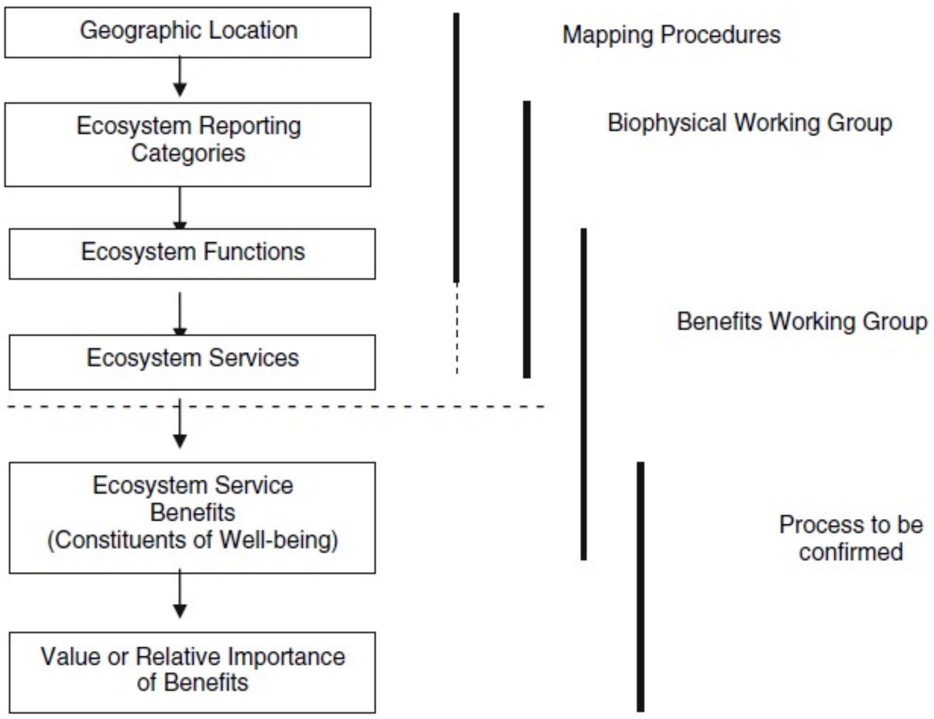

Figure A2.42 The process and key components of The South East Queensland (SEQ) Ecosystem Services Framework.

Source: Maynard, S., James, D. & Davidson, A. (2010) The development of an ecosystem services framework for South East Queensland. *Environmental Management,* 45:881–895 Figure 3, page 892. Reproduced with permission from Springer-Verlag

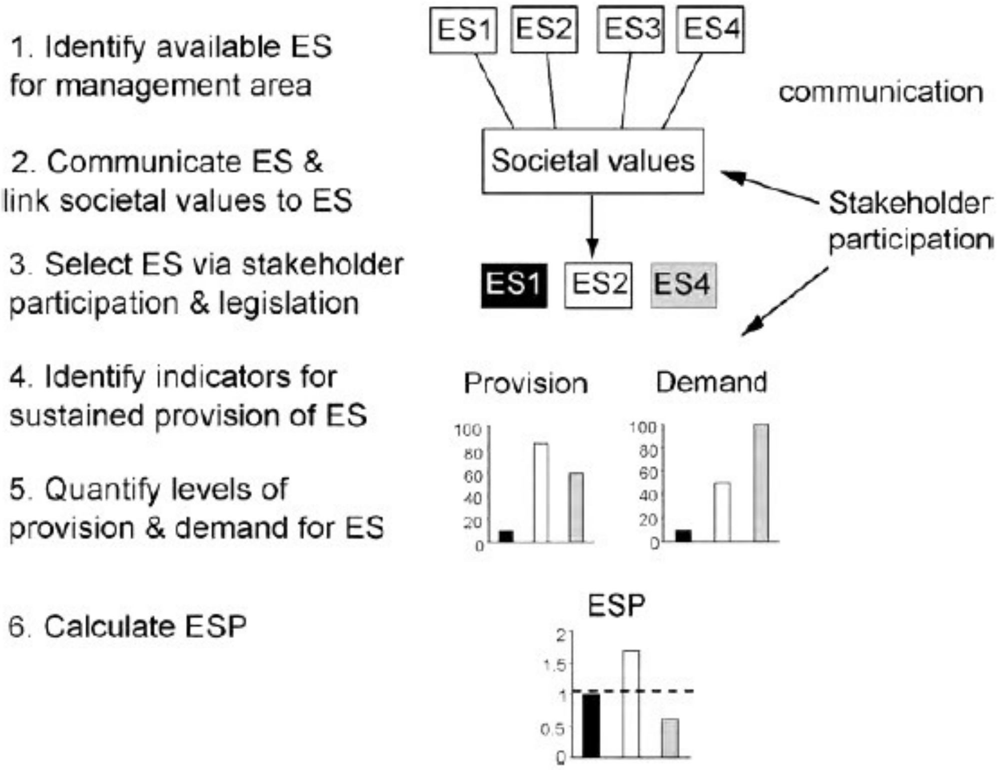

Figure A2.43 Major steps in the development of an Ecosystem Services Profile (ESP)

Source: Paetzold, A., Warren, P.H. & Maltby, L.L. (2010) A framework for assessing ecological quality based on ecosystem. *Ecological Complexity*, 7:273–281
Figure 3, page 278. Reproduced with permission from Elsevier

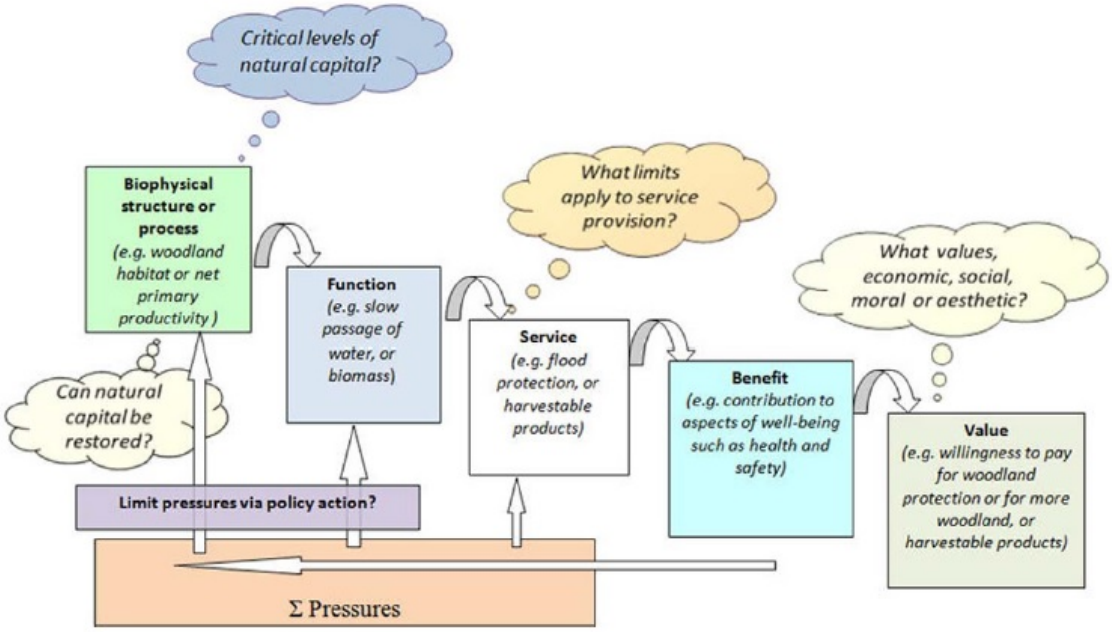

Figure A2.44. The ecosystem service cascade model. Adapted from Haines-Young and Potschin (2010).

Source: Potschin, M.B. & Haines-Young, R. H. (2010) Ecosystem services: Exploring a geographical perspective. *Progress in Physical Geography,* 35: 575–594
Figure 2, page 578. Reproduced with permission from SAGE

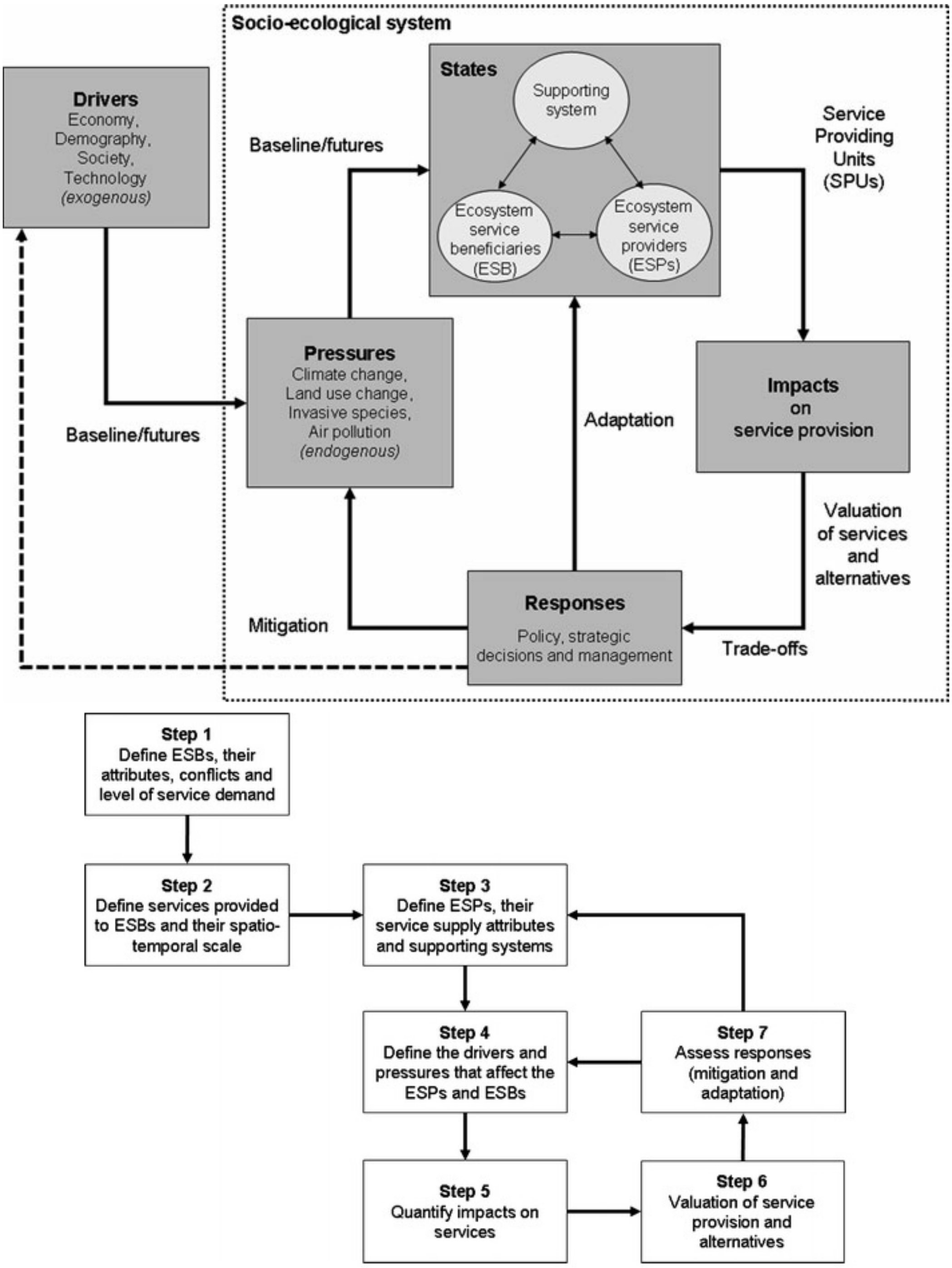

Figure A2.45 (a) A Framework for Ecosystem Service Provision (FESP) based on a modified Driver-Pressure-State-Impact-Response (DPSIR) framework. (b) Stepwise implementation strategy for the conceptual framework. ESP=Ecosystem Service Beneficiary, ESP= Ecosystem Service Provider

Source: Rounsevell, M.D.A., Dawson, T.P. & Harrison, P.A. (2010) A conceptual framework to assess the effects of environmental change on ecosystem services. *Biodiversity & Conservation*, 19: 2823–2842
Figures 1 & 3, pages 2827 & 2831. Reproduced with permission from Springer Netherlands

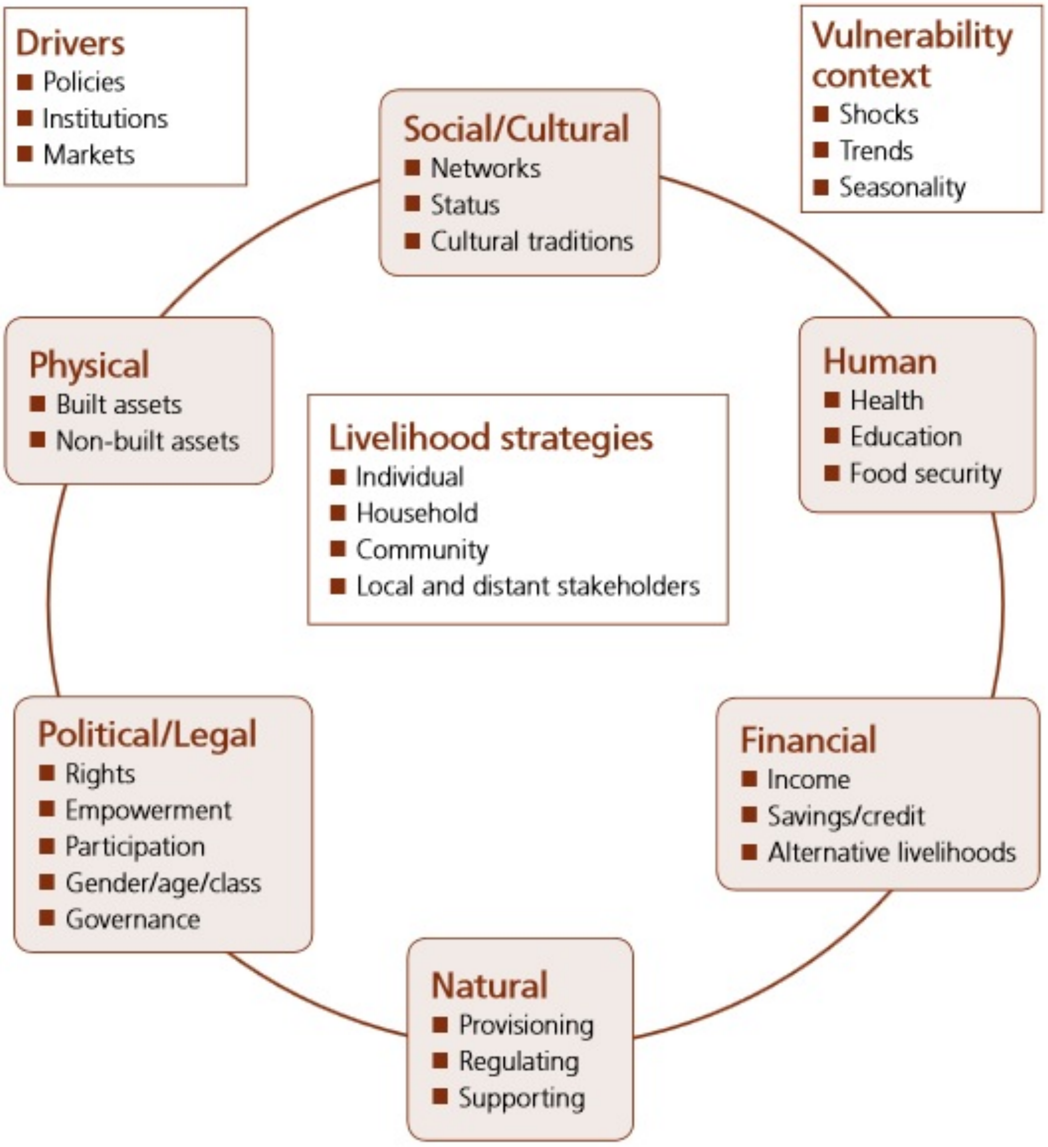

Figure A2.46 Modified Sustainable Livelihoods Framework

Source: Schreckenberg, K., Camargo, I., Withnall, K., Corrigan, C., Franks, P., Roe, D., et al (2010) *Social Assessment of Conservation Initiatives: A Review of Rapid Methodologies*. International Institute for Environment and Development (IIED): London. Figure 6, page 28. Reproduced with permission from IIED

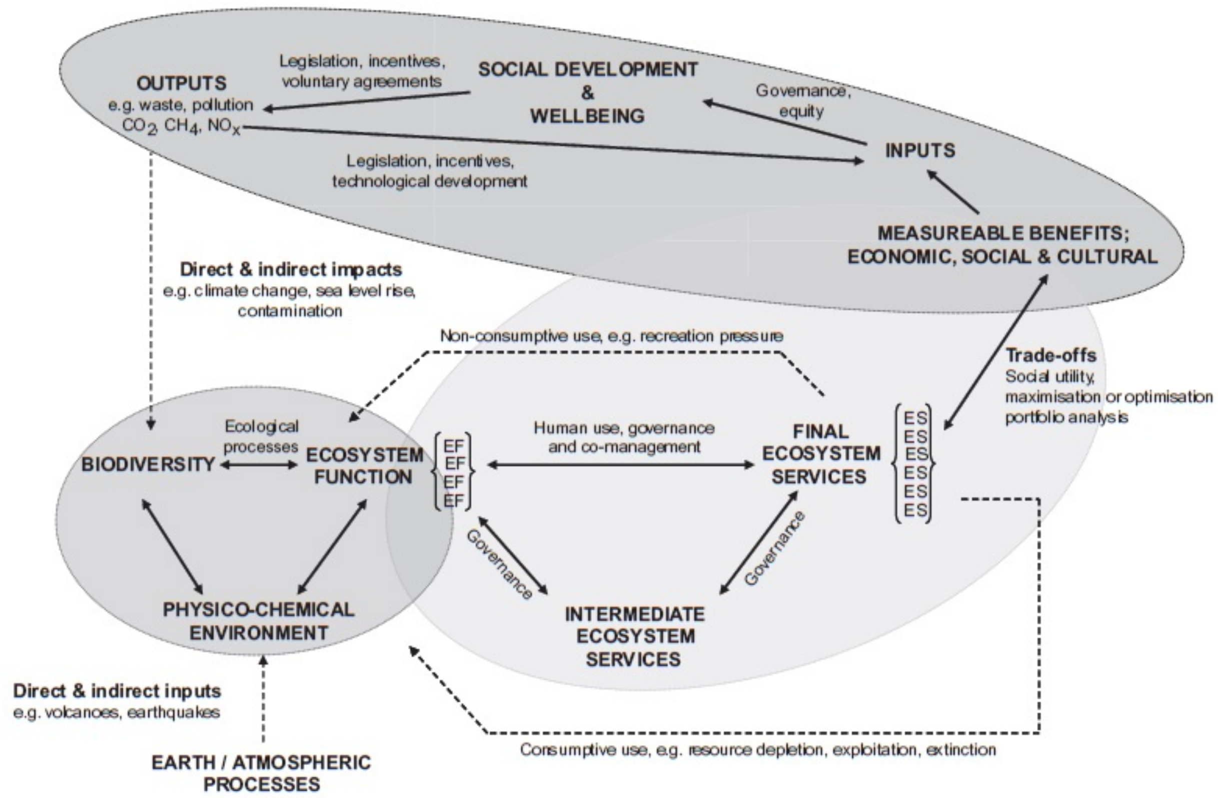

Figure A2.47 Efficiency framework for an ecosystem services approach to sustainability.

Source: White, P.C.L., Godbold, J.A., Solan, M., Wiegand, J. & Holt, A.R. (2010) *Ecosystem services and policy: a review of coastal wetland ecosystem services and an efficiency-based framework for implementing the ecosystem approach*. In: Hester, R.E. & Harrison, R.M. (Eds) Ecosystem Services. Issues in Environmental Science and Technology, 30:29-51 http://dx.doi.org/10.1039/9781849731058-00029
Figure 1, page 42. Reproduced with permission from the Royal Society of Chemistry

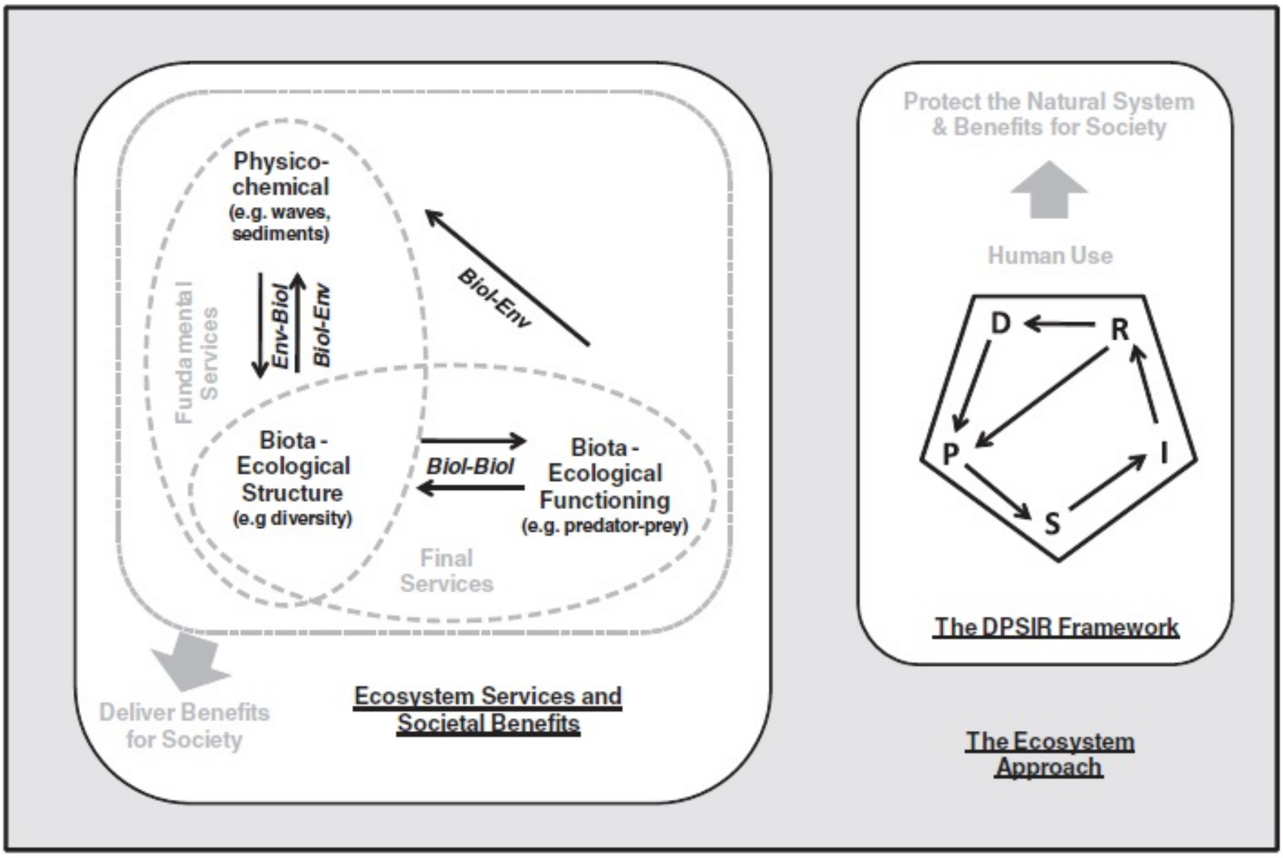

Figure A2.48 The DPSIR framework and the ecosystem services and societal benefits set within an overall framework of The Ecosystem Approach.

Source: Atkins, J.P., Burdon, D., Elliott, M. & Gregory, A.J. (2011) Management of the marine environment: Integrating ecosystem services and societal benefits with the DPSIR framework in a systems approach. *Marine Pollution Bulletin*, 62:215–226.
Figure 3, page 220. Reproduced with permission from Elsevier

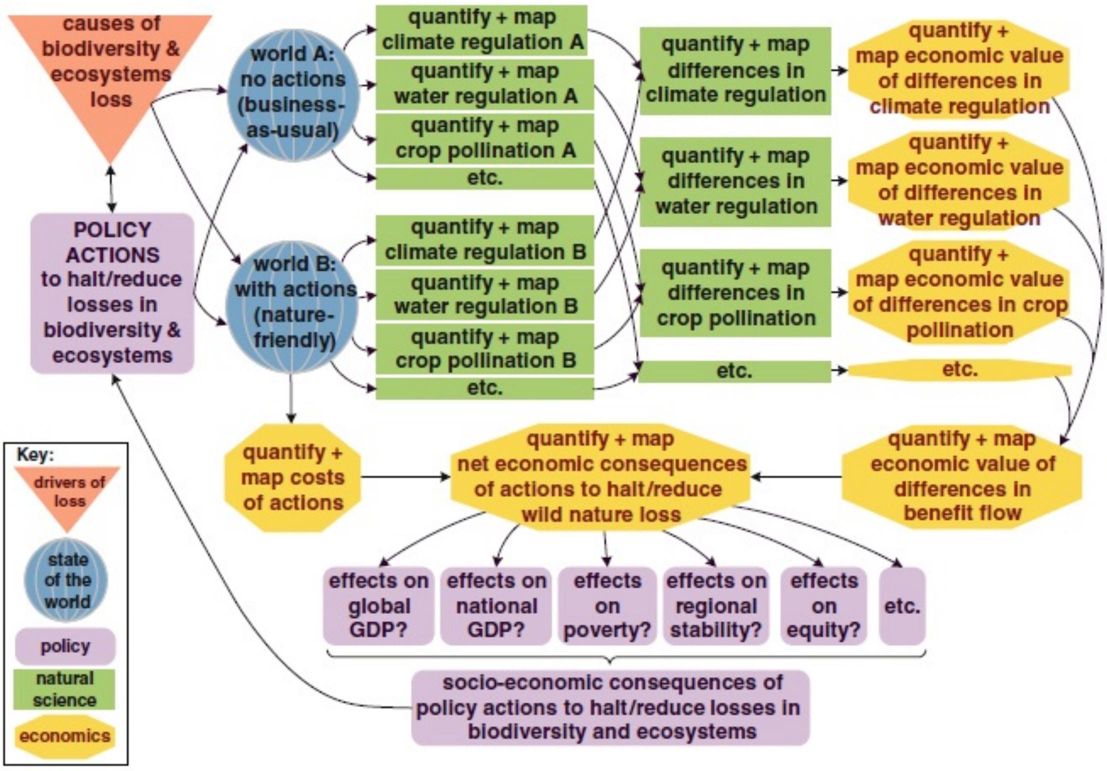

Figure A2.49 Framework for assessing the economic consequences of losing biodiversity and ecosystems.

Source: Balmford, A., Fisher, B., Green, R.E., Naidoo, R., Strassburg, B., Turner, R.K. & Rodrigues, A. S. L. (2011) Bringing ecosystem services into the real world: an operational framework for assessing the economic consequences of losing wild nature. *Environmental & Resource Economics,* 48:161–175
Figure 2, page 164. Reproduced with permission from Springer Netherlands

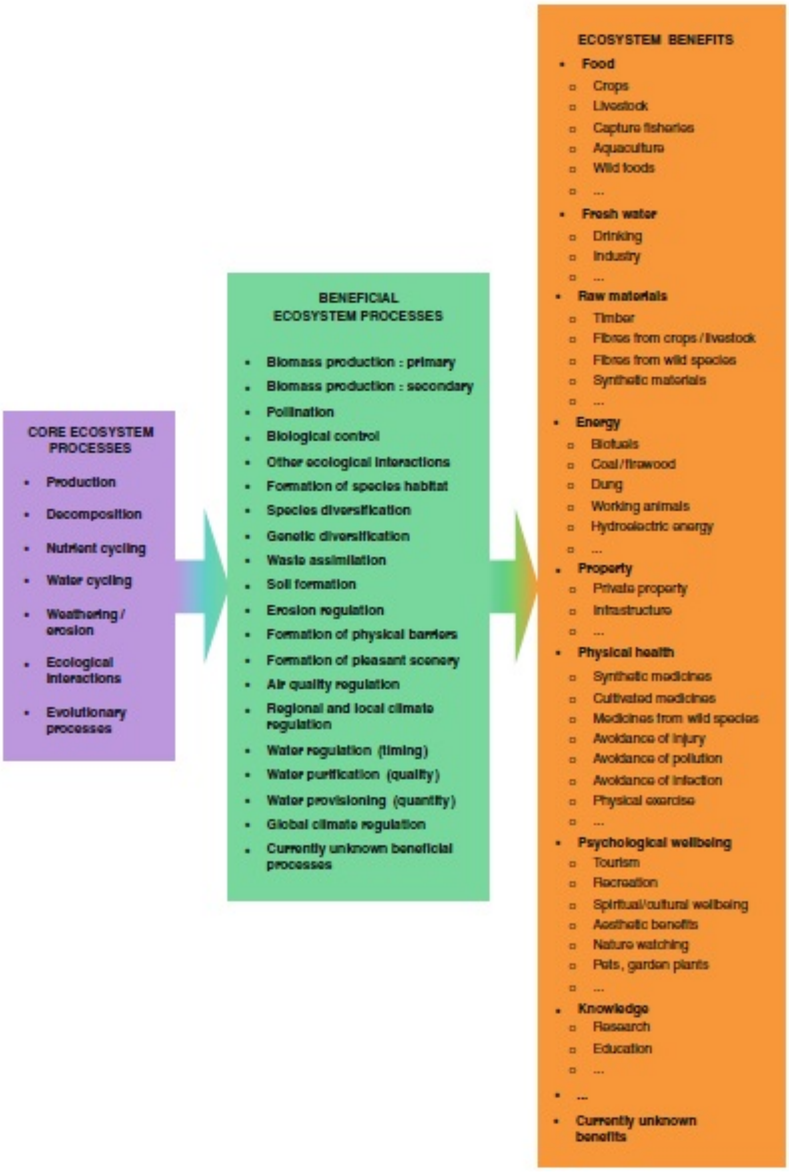

Figure A2.50 Illustration of the relationship between core ecosystem processes, beneficial ecosystem processes, and ecosystem benefits.

Source: Balmford, A., Fisher, B., Green, R.E., Naidoo, R., Strassburg, B., Turner, R.K. & Rodrigues, A. S. L. (2011) Bringing ecosystem services into the real world: an operational framework for assessing the economic consequences of losing wild nature. *Environmental & Resource Economics,* 48:161–175
Figure 3, page 165. Reproduced with permission from Springer Netherlands

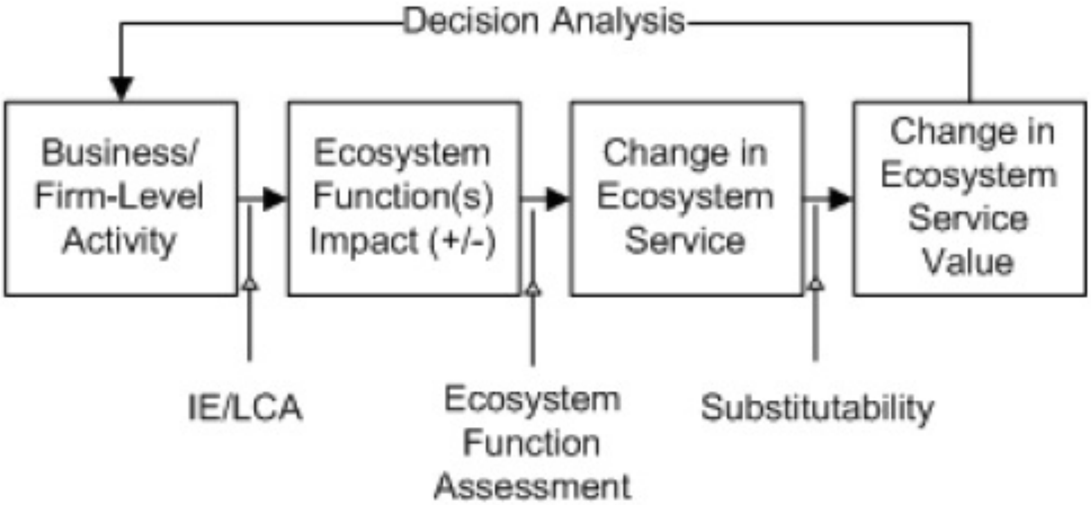

Figure A2.51 Firm-level ecosystem service valuation framework (LCA =Life Cycle Assessment).

Source: Comello, S.D. & Lepech, M.D. (2011) *A framework for multiphysics modeling of natural environments for valuation of privately owned ecosystem services.* IEEE International Symposium on Sustainable Systems and Technology (ISSST), 16-18 May 2011, Chicago, IL Figure 1. Reproduced with permission from IEEE

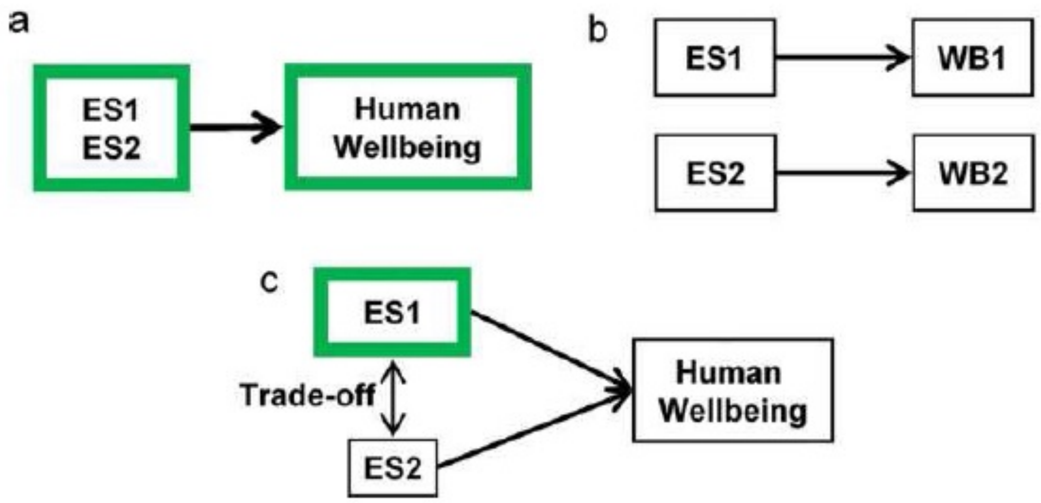

Figure A2.52 Conceptualizations of ES and human well-being (WB) that do not disaggregate human well-being (i.e. the beneficiaries). Hypothetical increases in ES and well-being are indicated by bold boxes. (a) Simplistic aggregated view of well-being and ES, which leads to assumptions that increases in ES will lead to increases in well-being. (b) Elements of human well-being are disaggregated, recognizing that different ES may contribute to different elements of well-being. (c) ES are disaggregated to explore trade-offs between them.

Source: Daw, T., Brown, K., Rosendo, S. & Pomeroy, R. (2011) Applying the ecosystem services concept to poverty alleviation: the need to disaggregate human well-being. *Environmental Conservation*, 38: 370–379.
Figure 1, page 371. Reproduced with permission from the Foundation of Environmental Conservation

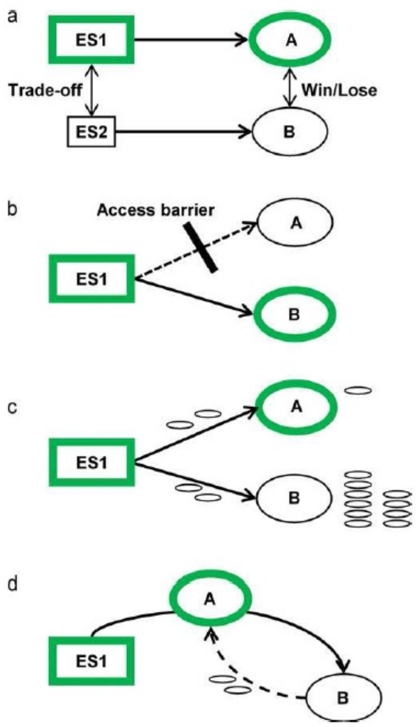

Figure A2.53 Aspects of ES and human well-being relevant to poverty alleviation that are highlighted by disaggregating human beneficiaries of ES

Source: Daw, T., Brown, K., Rosendo, S. & Pomeroy, R. (2011) Applying the ecosystem services concept to poverty alleviation: the need to disaggregate human well-being. *Environmental Conservation*, 38: 370–379.
Figure 2, page 372. Reproduced with permission from the Foundation of Environmental Conservation

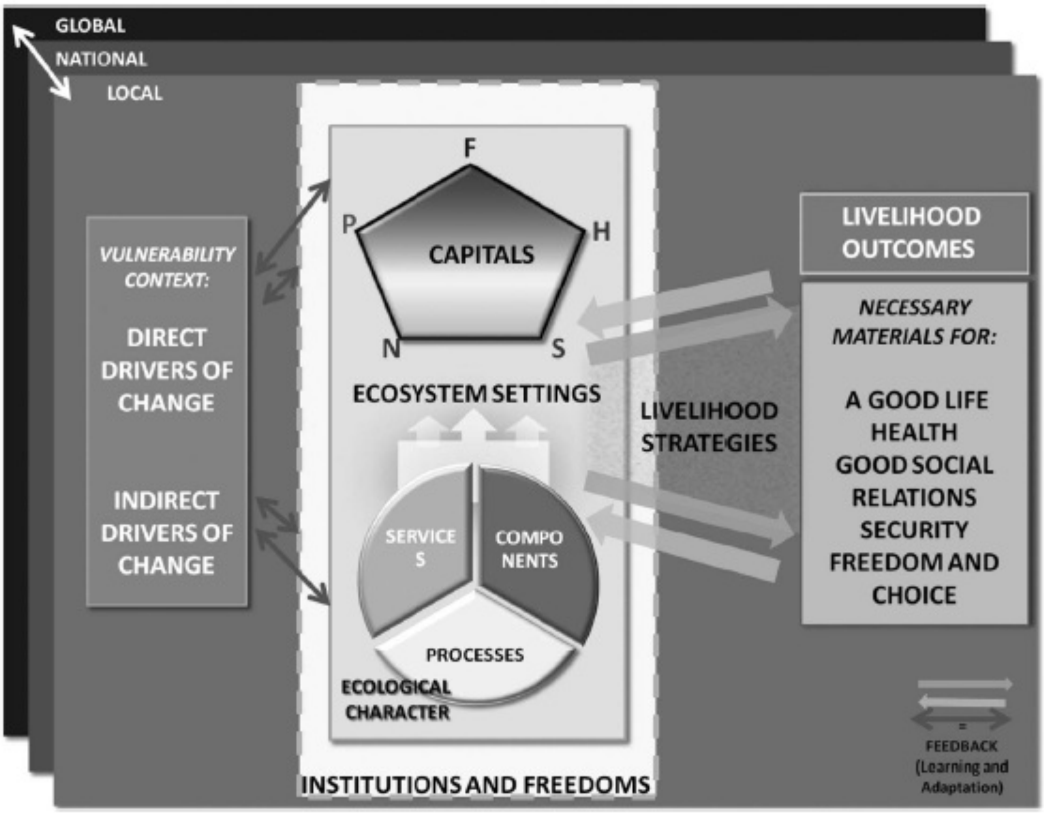

Figure A2.54 Framework for assessing wetlands–livelihood interlinkages

Source: Kumar, R., Horwitz, P., Milton, G.R., Sellamuttu, S.S., Buckton, S.T., Davidson, N.C. et al (2011) Assessing wetland ecosystem services and poverty interlinkages: a general framework and case study. *Hydrological Sciences Journal,* 56:1602-1621
Figure 2, page 1609. Reproduced with permission from Taylor & Francis

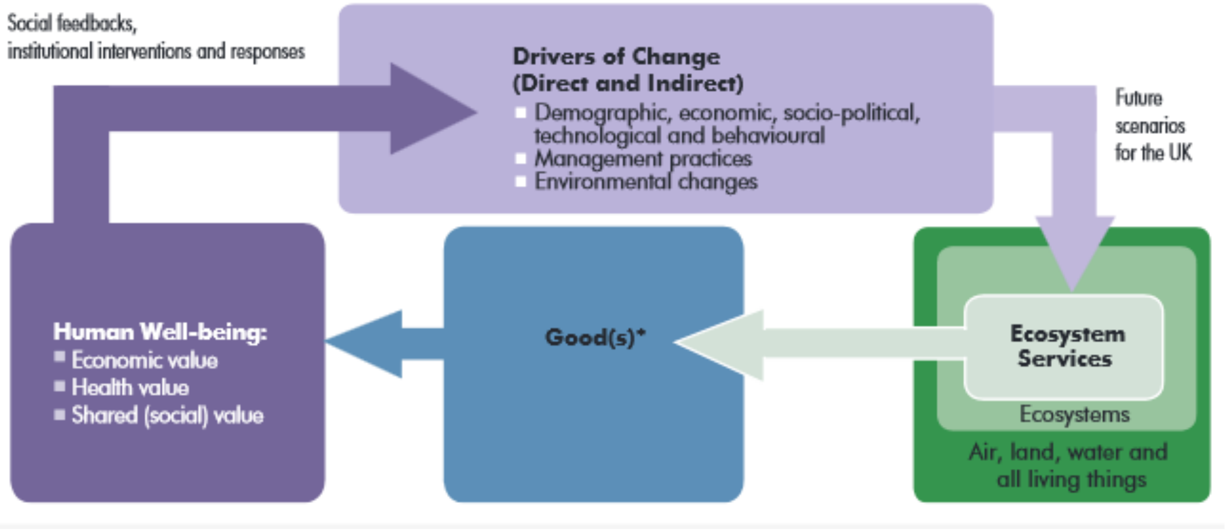

Figure A2.55 UK NEA Overall Conceptual Framework: showing the links between ecosystems, ecosystem services, good(s), valuation, human well-being, change processes and scenarios.

Source: UK National Ecosystem Assessment (UK NEA) (2011) *UK National Ecosystem Assessment Technical Report.* UNEP-WCMC: Cambridge
Figure 2.1, page 13 (Chapter 2, Conceptual Framework and Methodology, M. Mace and I. Bateman). Reproduced with permission from the UK NEA.

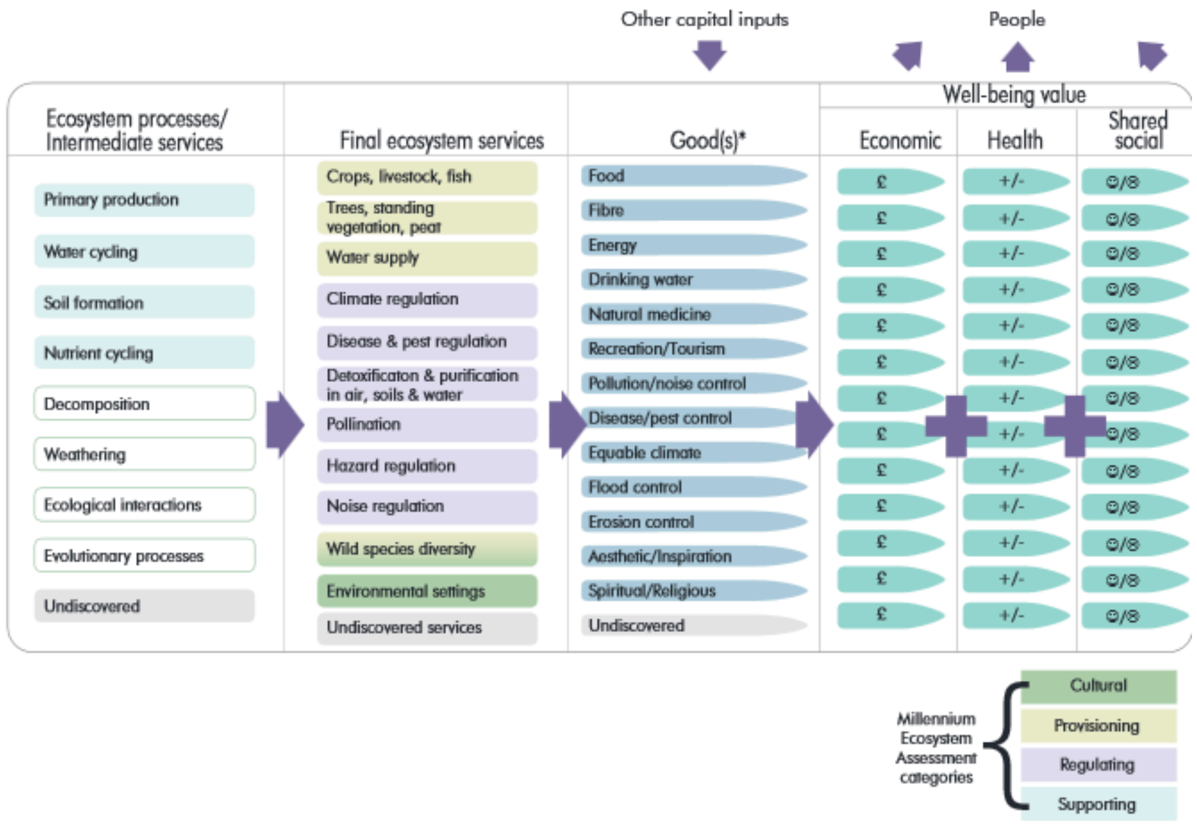

Figure A2.56 UK NEA Ecosystem services framework: The full set of ecosystem processes, services, goods/benefits and values used in the UK NEA**.**

Source: UK National Ecosystem Assessment (UK NEA) (2011) *UK National Ecosystem Assessment Technical Report.* UNEP-WCMC: Cambridge
Figure 2.3, page 18 (Chapter 2, Conceptual Framework and Methodology, M. Mace and I. Bateman et al). Reproduced with permission from the UK NEA.

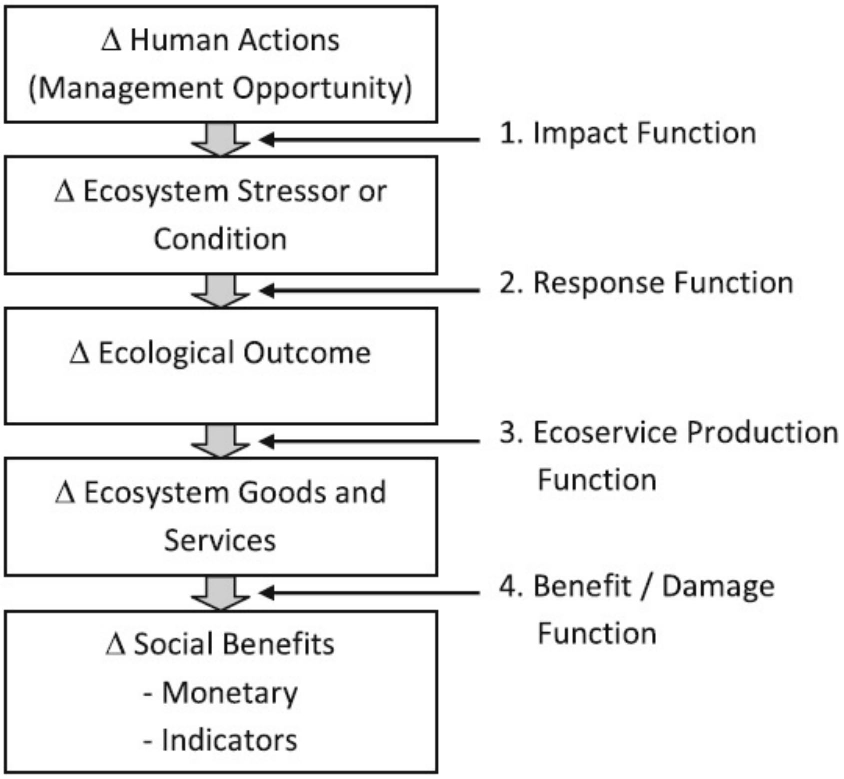

Figure A2.57 Framework to estimate economic benefits of a management change.

Source: Wainger, L. & Mazzotta, M. (2011) Realizing the potential of ecosystem services: a framework for relating ecological changes to economic benefits. *Environmental Managemen*t, 48: 710–733
Figure 1, page 713. Reproduced with permission from Springer-Verlag

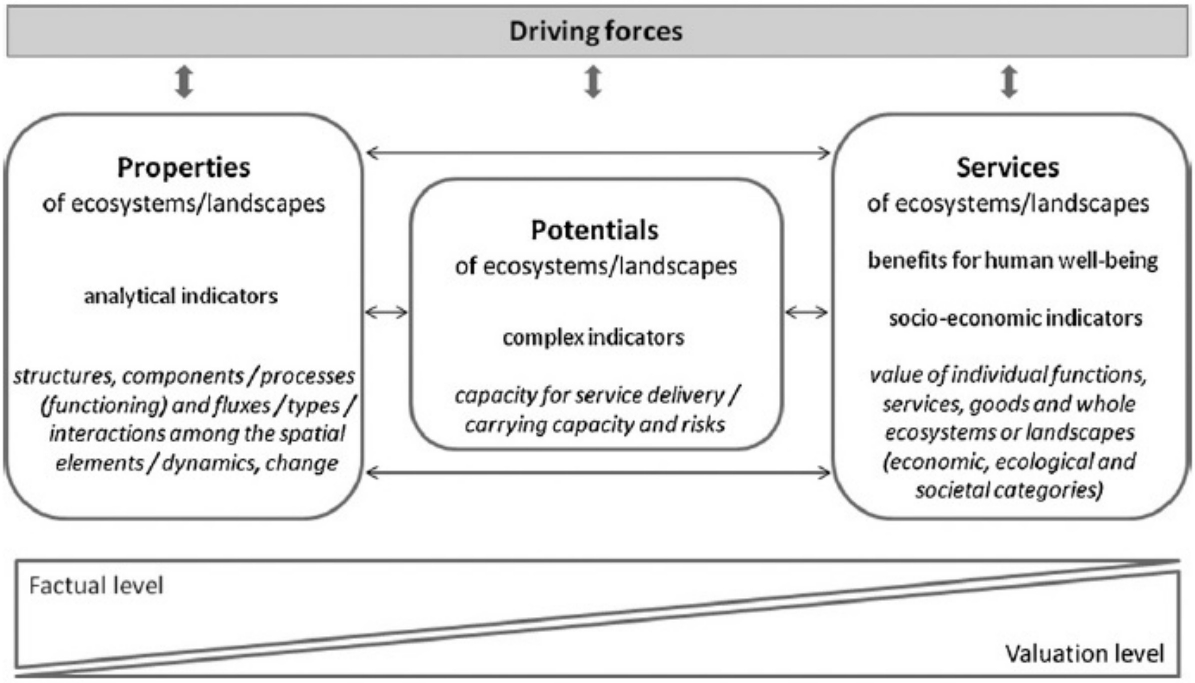

Figure A2.58 Ecosystem Properties, Potentials, and Services (EPPS) framework for the analysis of ecosystem/ landscape services.

Source: Bastian, O., Hasse, D. & Grunewald, K. (2012) Ecosystem properties, potentials and services – The EPPS conceptual framework and an urban application example. *Ecological Indicators*, 21:7–16
Figure 1, page 10. Reproduced with permission from Elsevier

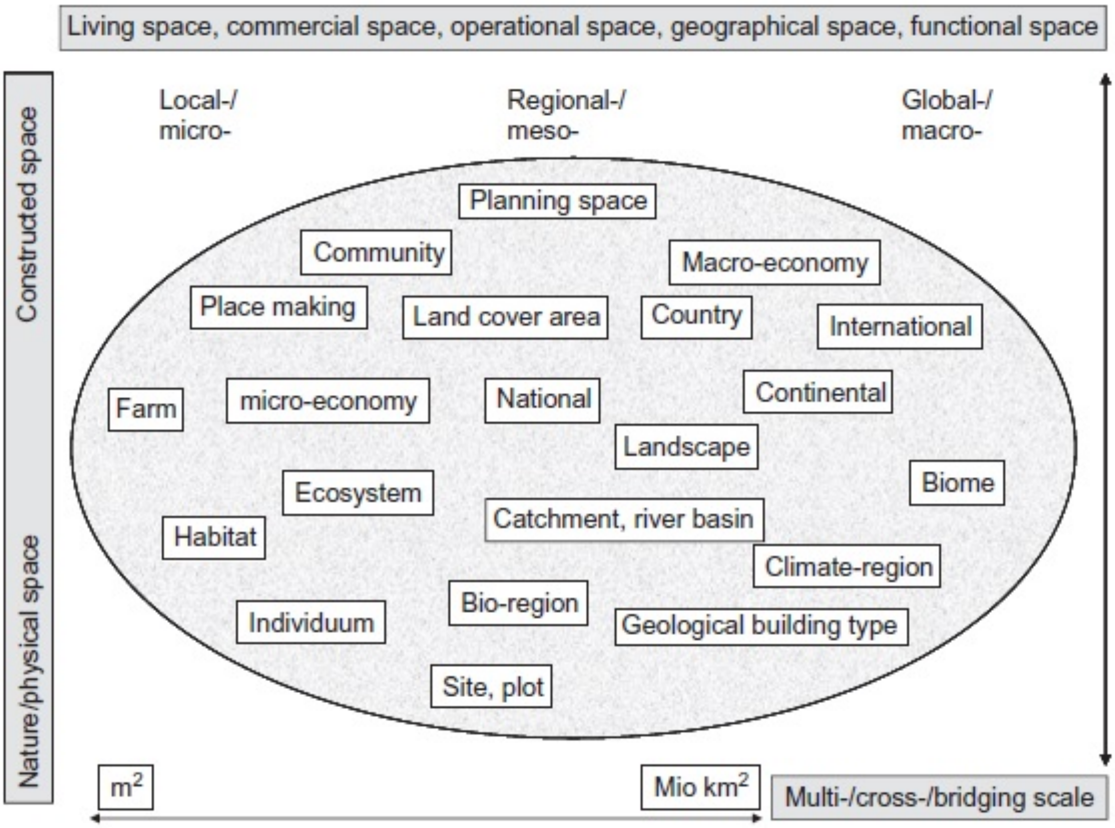

Figure A2.59 Selected spatially relevant phenomena reflecting different scales

Source: Bastian, O., Grunewald, K. & Syrbe, R.U (2012) Space and time aspects of ecosystem services, using the example of the EU Water Framework Directive. *International Journal of Biodiversity Science, Ecosystem Services & Management*, 8: 5–16
Figure 1, page 9. Reproduced with permission from Taylor & Francis

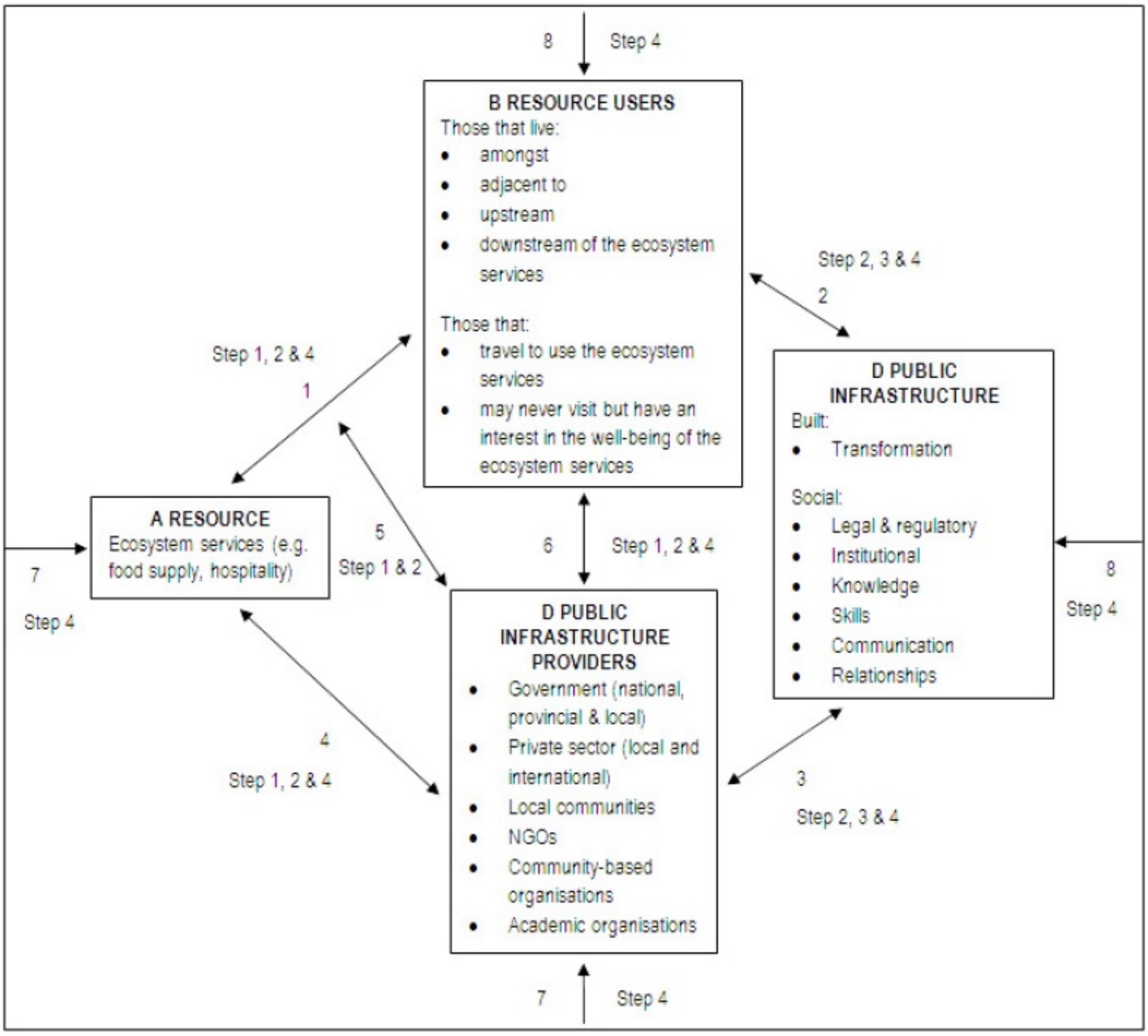

Figure A2.60. A framework for analysing the robustness of social-ecological systems from an institutional perspective, showing examples of how it relates to an estuarine system. Step 1 - ecosystem services supply and demand assessment; Step 2 - future estuary roles identification; Step 3 - enterprise opportunity identification; Step4 - enterprise risk assessment. Adapted from Anderies et al. (2004). See source for further information regarding no. 1-8.

Source: Bowd, R., Quinn, N., Kotze, D.C., Hay, D.G. & Mander M. (2012) The identification of potential resilient estuary-based enterprises to encourage economic empowerment in South Africa: a toolkit approach. *Ecology and Society,* 17:15 Figure 1. Reproduced with permission The Resilience Alliance and The Authors

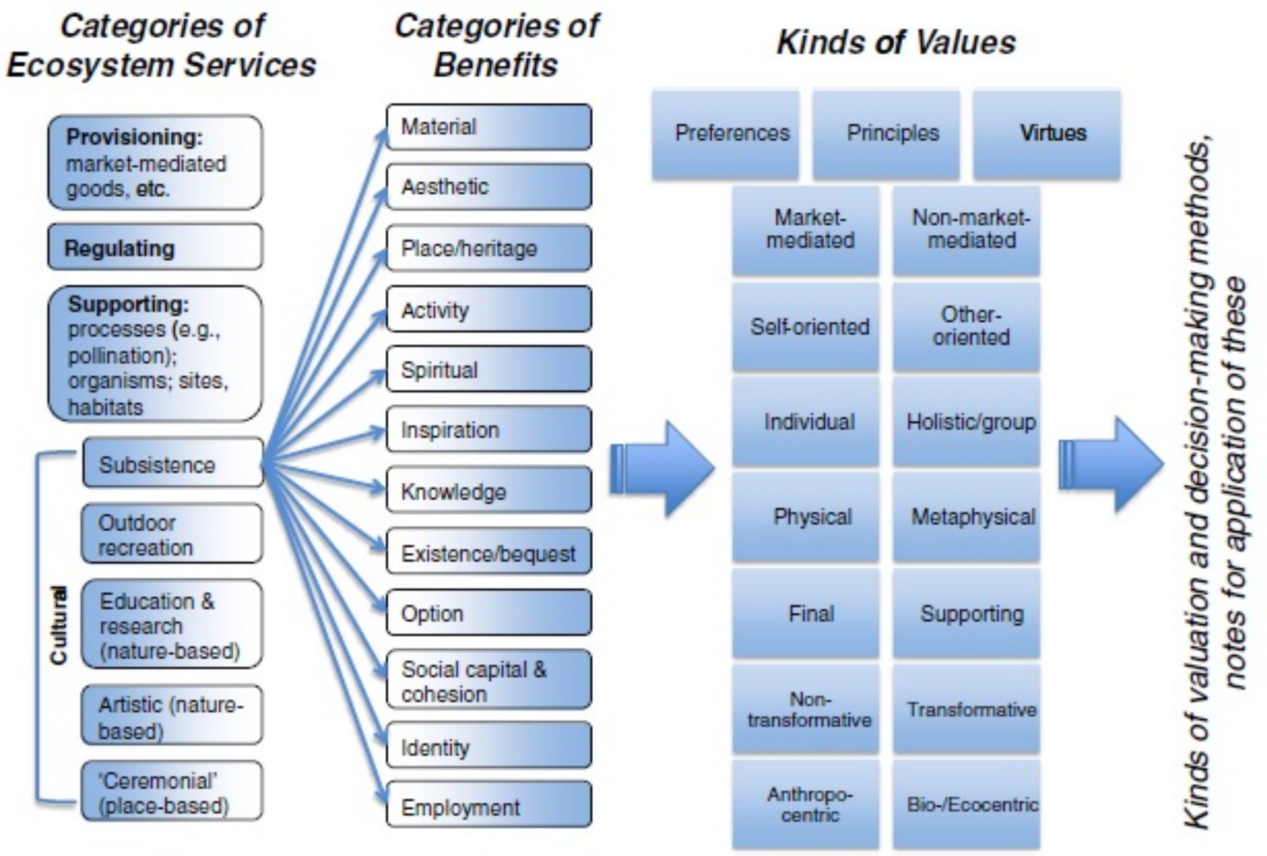

Figure A2.61 Suggested use of the typologies of ecosystem services and values. Modified from Chan et al. (2011).

Source: Chan, K.M.A., Satterfield,T. & Goldstein, J. (2012) Rethinking ecosystem services to better address and navigate cultural values. *Ecological Economics,* 74: 8–18
Figure 1, page 13. Reproduced with permission from Elsevier

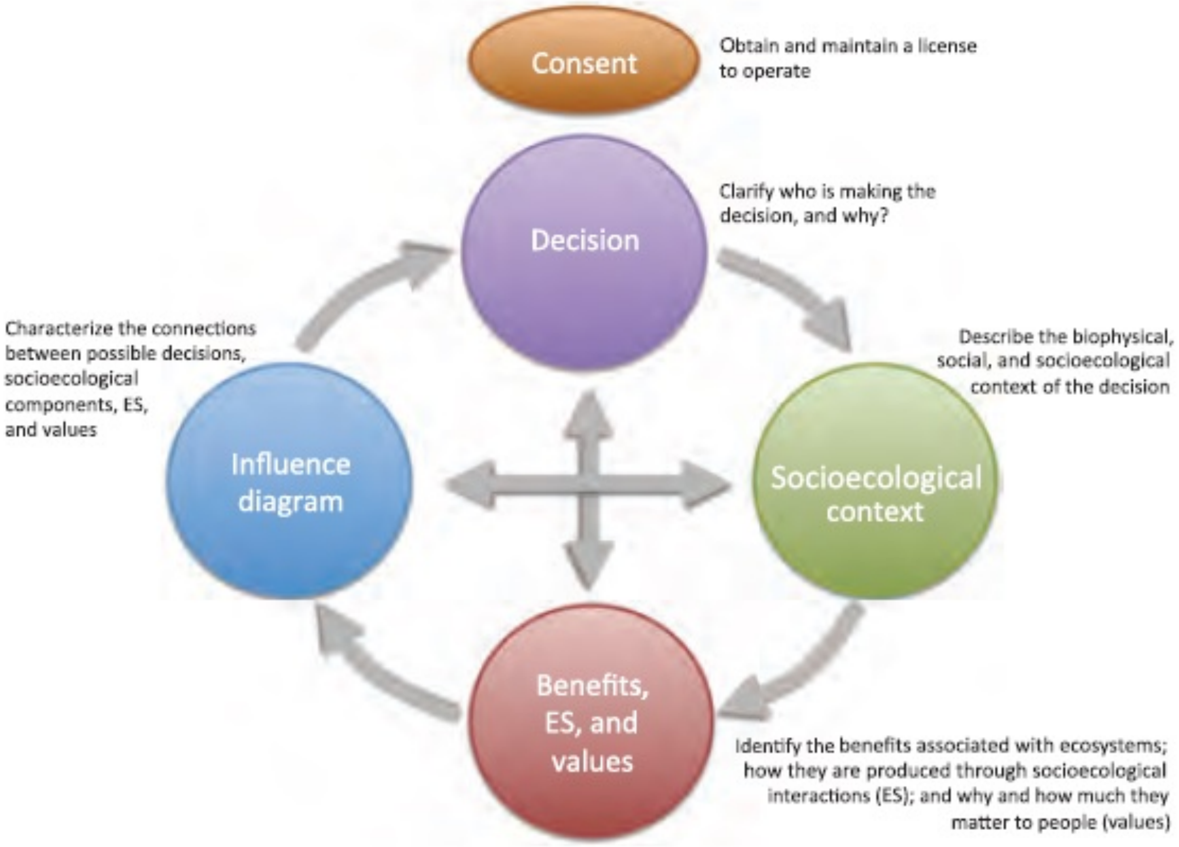

Figure A2.62 Framework for characterizing ES that might be affected by management or planning.

Source: Chan, K.M.A., Guerry, A.D., Balvanera, P., Klain, S., Satterfield, T., Basurto, X. et al (2012) Where are cultural and social in ecosystem services? A framework for constructive engagement. *BioScience,* 62:744–756.
Figure 1, page 748. Reproduced with permission from the American Institute of Biological Sciences

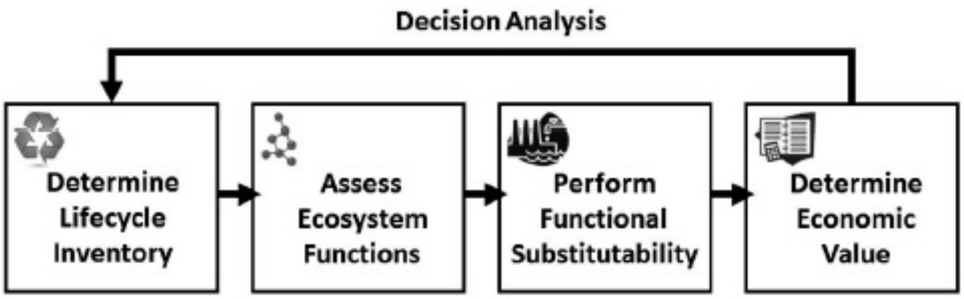

Figure A2.63 Firm-level ecosystem service valuation framework. Modifed from Comello & Lepech (2011).

Source: Comello, S. D., Lepech, M. D. & Schwegler, B. R. (2012) Project-level assessment of environmental effect : ecosystem services approach to sustainable management and development. *Journal of Management in Engineering,* 28:5-12
Figure 1, page 6. Reproduced with permission from the American Society of Civil Engineers

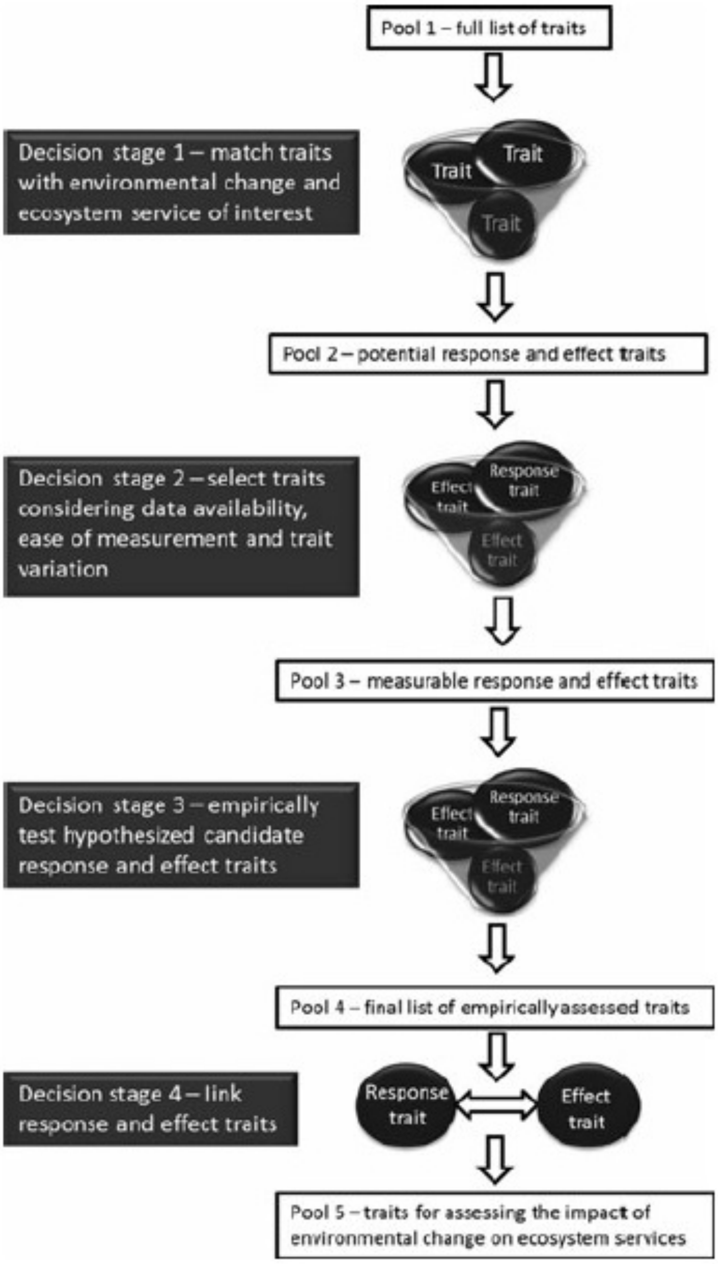

Figure A2.64 Trait-selection framework.

Source: Luck, G.W., Lavorel, S., McIntyre, S. & Lumb, K. (2012) Improving the application of vertebrate trait-based frameworks to the study of ecosystem services. *Journal of Animal Ecology*, 81:1065–1076
Figure 1, page 1070. Reproduced with permission from the British Ecological Society

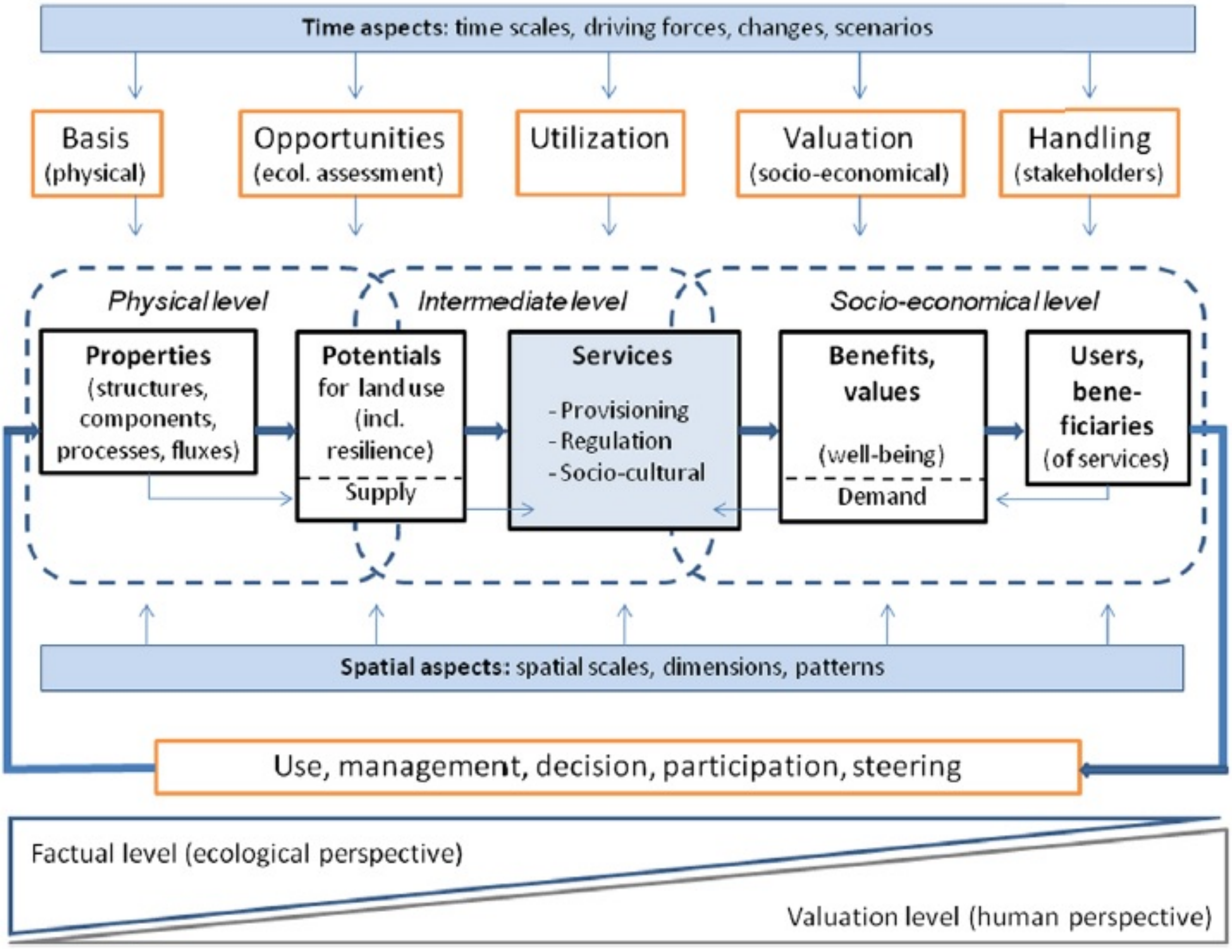

Figure A2.65 Conceptual framework for the analysis of ecosystem services—the extended EPPS framework.

Source: Bastian, O., Syrbe, R.U., Rosenberg, M., Rahe, D., & Grunewald, K. (2013) The five pillar EPPS framework for quantifying, mapping and managing ecosystem services. *Ecosystem Services,* 4:15–24
Figure 1, page 16. Reproduced with permission from Elsevier

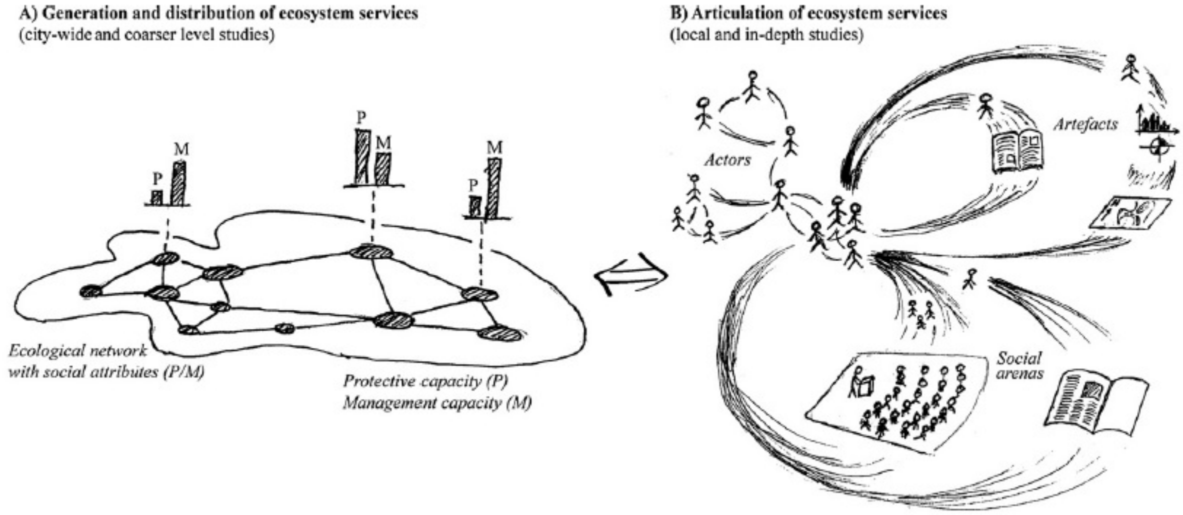

Figure A2.66 A framework for studying the social production of ecosystem services

Source: Ernston, H. (2013) The social production of ecosystem services: a framework for studying environmental justice and ecological complexity in urbanized landscapes. *Landscape and Urban Planning*, 109:7– 17
Figure 1, page 11. Reproduced with permission from Elsevier

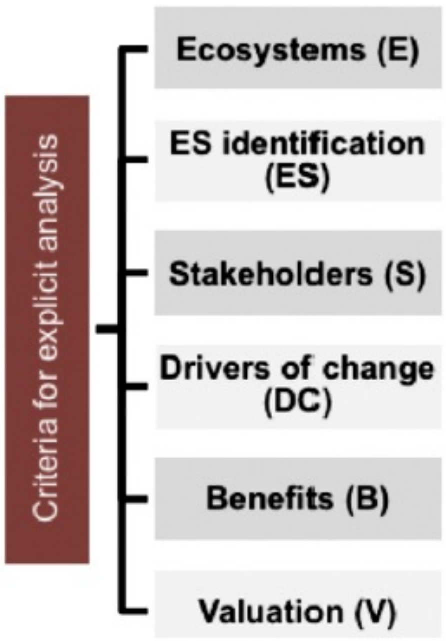

Figure A2.67 Criteria for explicit analysis for consideration of ecosystem services in Strategic Environmental Assessment**.**

Source: Honrado, J.P., Vieira, C., Soares, C., Monteiro, M.B., Marcos, B., Pereira, H.M. & Partidário, M.R. (2013) Can we infer about ecosystem services from EIA and SEA practice? A framework for analysis and examples from Portugal. *Environmental Impact Assessment Review*, 40:14–24
Figure 3, page 20. Reproduced with permission from Elsevier

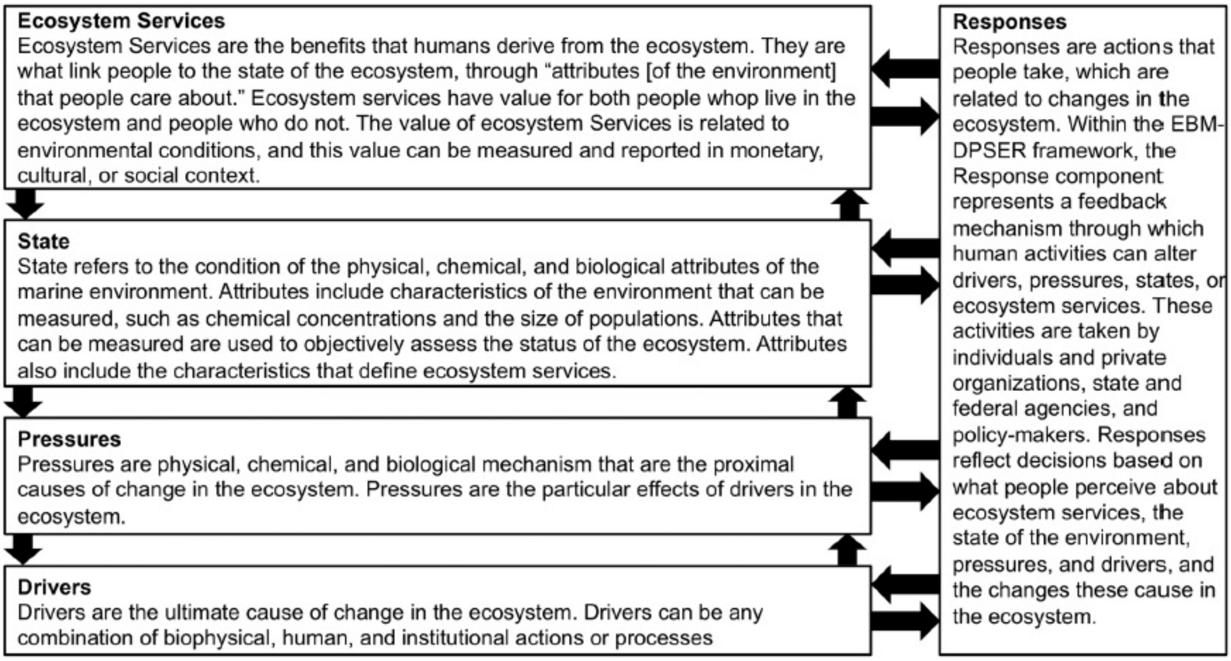

Figure A2.68 The EBM-DPSER model.

Source: Kelble, C.R., Loomis, D.K., Lovelace, S., Nuttle, W.K., Ortner, P.B, Fletcher, P. et al. (2013) The EBM-DPSER Conceptual Model: integrating ecosystem services into the DPSIR framework. *PLoS ONE*, 8: e70766
Figure 2, page 5. Open Access

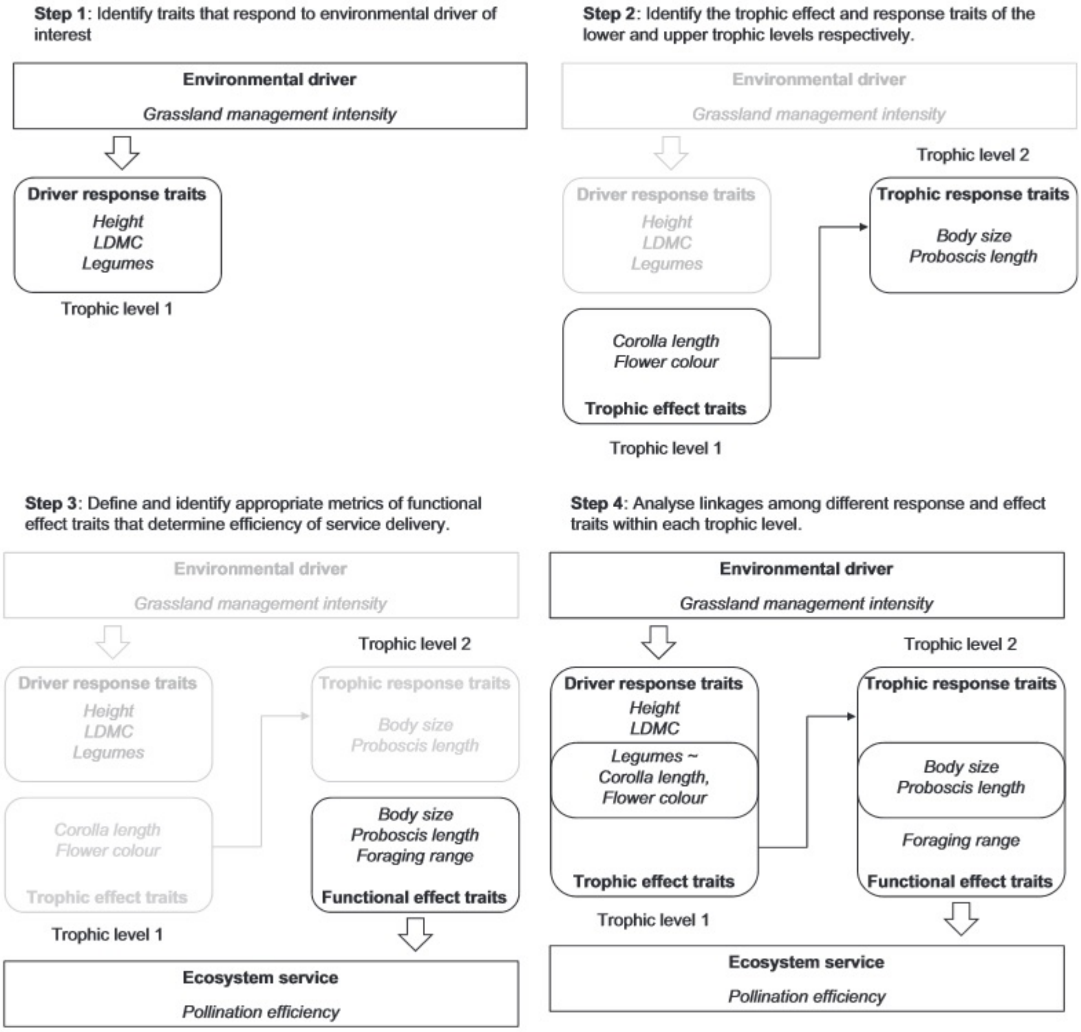

Figure A2.69 Method for articulating functional responses and effects within and across two trophic levels to predict changes in ecosystem functioning, and methodological steps for its application.

Source: Lavorel, S., Storkey, J., Bardgett, R.D., de Bello, F., Berg, M.P. Le Roux, X. (2013) A novel framework for linking functional diversity of plants with other trophic levels for the quantification of ecosystem services. *Journal of Vegetation Science,* 24:942–948
Figure 1, page 944. Reproduced with permission from the International Association for Vegetation Science

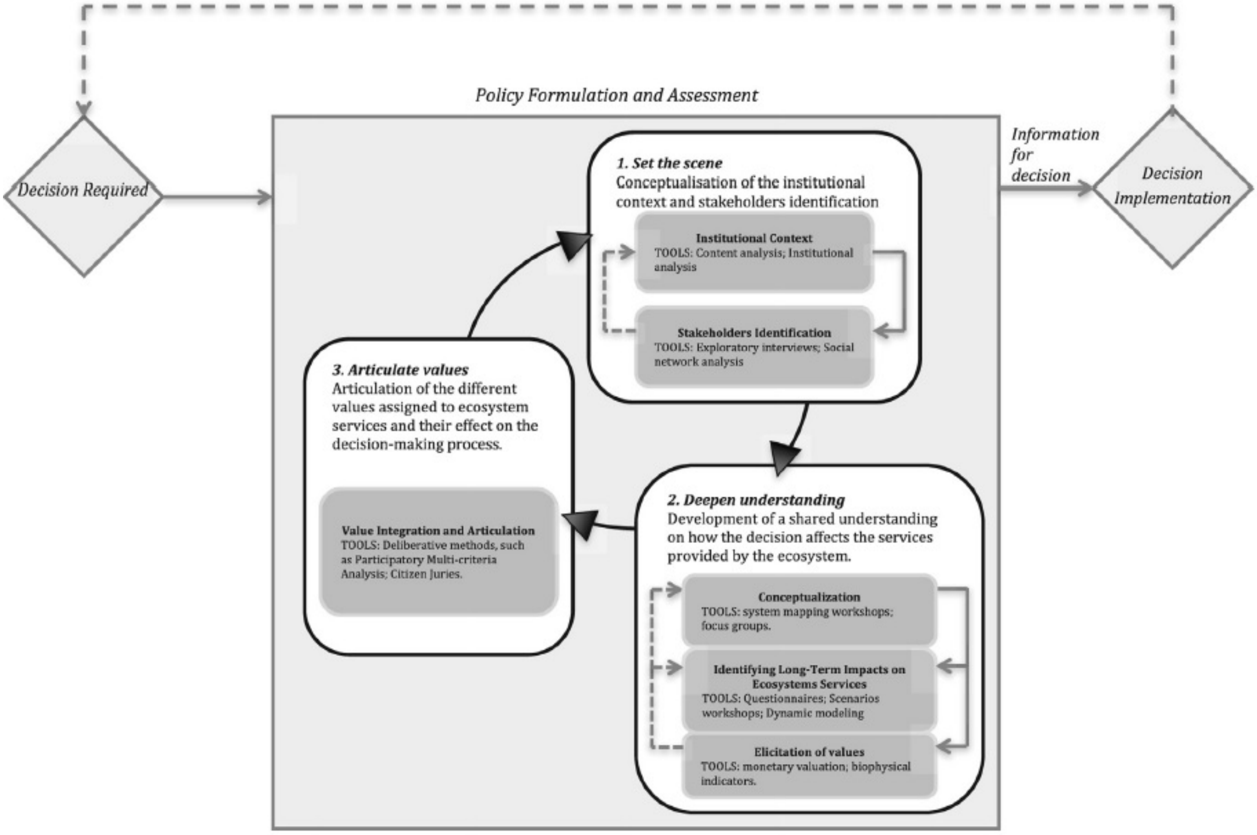

Figure A2.70 Integrated participatory framework for the valuation of marine and coastal ecosystem services

Source: Lopes, R. & Videira, N. (2013) Valuing marine and coastal ecosystem services: An integrated participatory framework. *Ocean & Coastal Management*, 84:153-163
Figure 1, page 157. Reproduced with permission from Elsevier

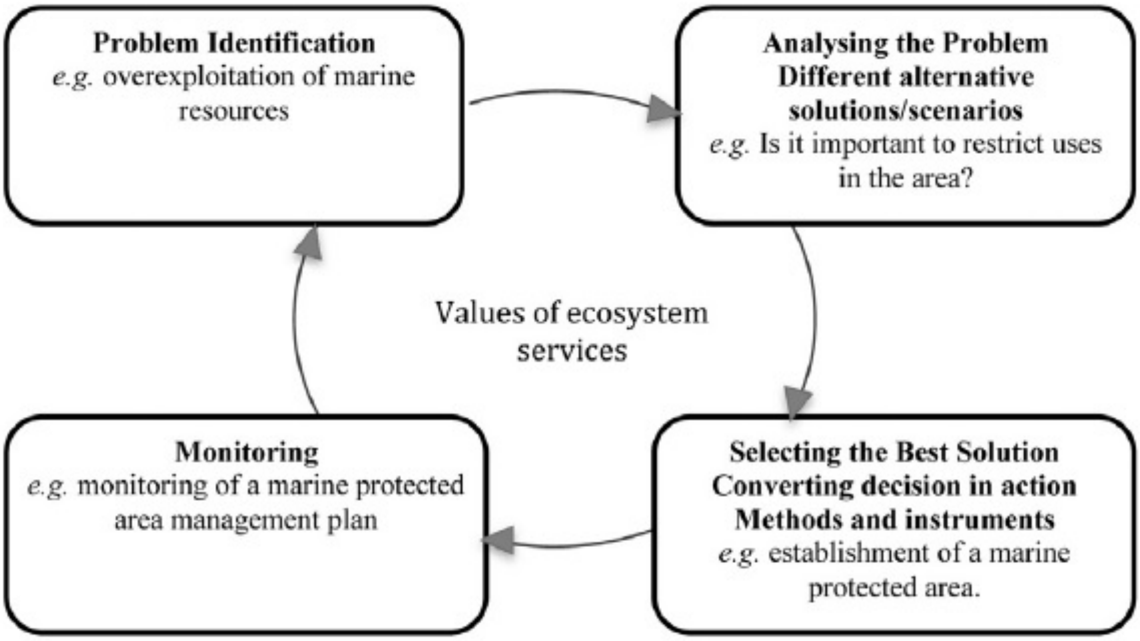

Figure A2.71 Decision-making process in marine and coastal environments. Adapted from Antunes & Santos (1999).

Source: Lopes, R. & Videira, N. (2013) Valuing marine and coastal ecosystem services: An integrated participatory framework. *Ocean & Coastal Management*, 84:153-163
Figure 2, page 159. Reproduced with permission from Elsevier

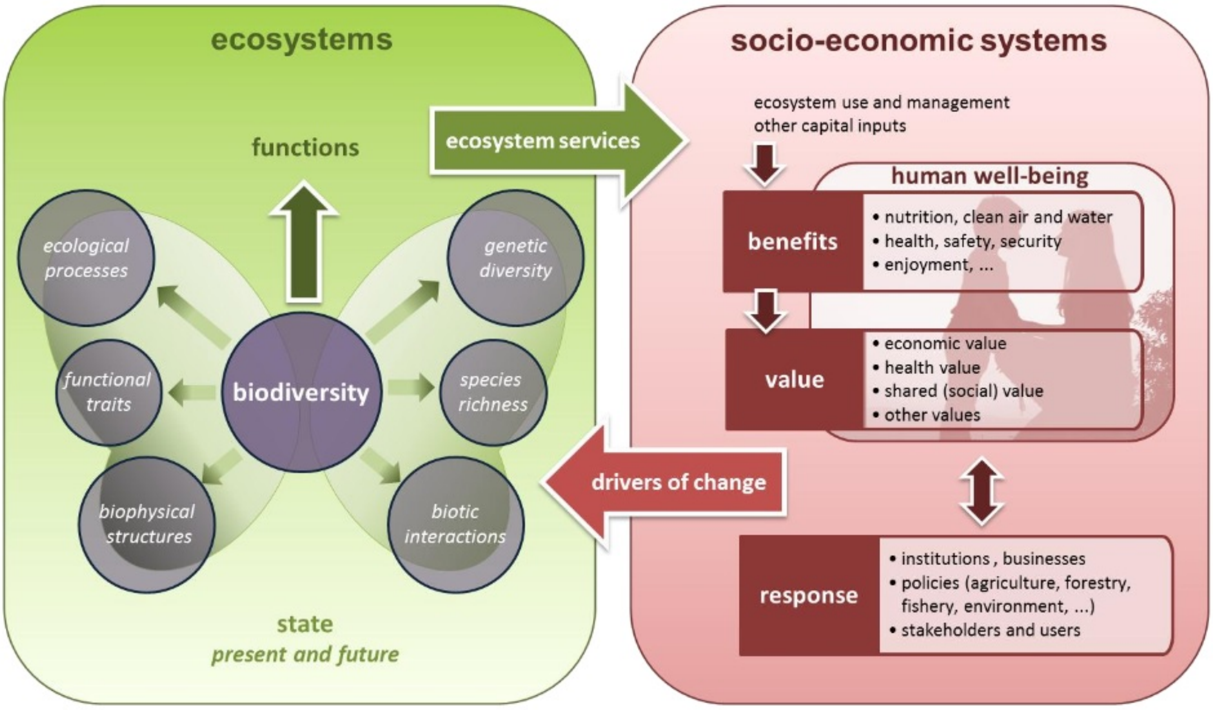

Figure A2.72. Conceptual framework for EU wide ecosystem assessments

Source: Maes, .J, Teller, A., Erhard, M., Liquete, C., Braat, L., Berry, P., et al (2013) *Mapping and assessment of ecosystems and their services. An analytical framework for ecosystem assessments under action 5 of the EU biodiversity strategy to 2020.* Publications office of the European Union, Luxembourg.
Figure 2, page 17. Reproduced with permission from European Union

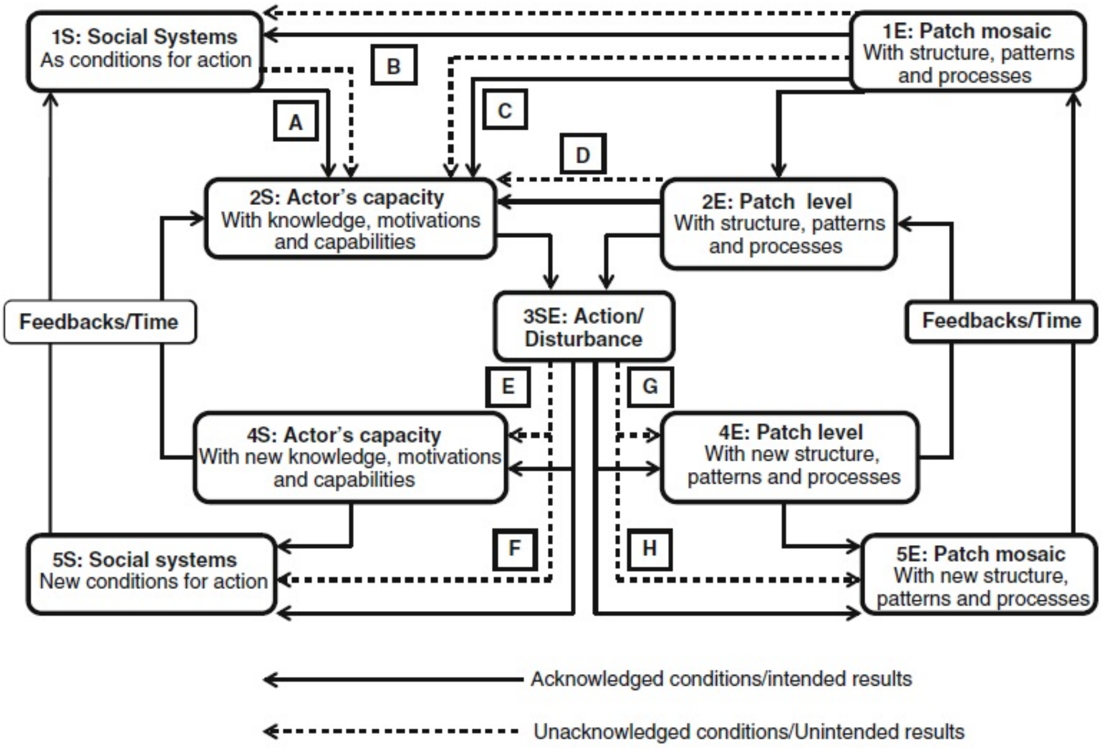

Figure A2.73 Structuration of social ecological complex adaptive systems.

Source: Morse, W.C., McLaughlin, W.J., Wulfhorst , J.D. & Harvey, C. (2013) Social ecological complex adaptive systems: a framework for research on payments for ecosystem services. *Urban Ecosystems,* 16:53–77
Figure 1, page 58. Reproduced with permission from Springer US

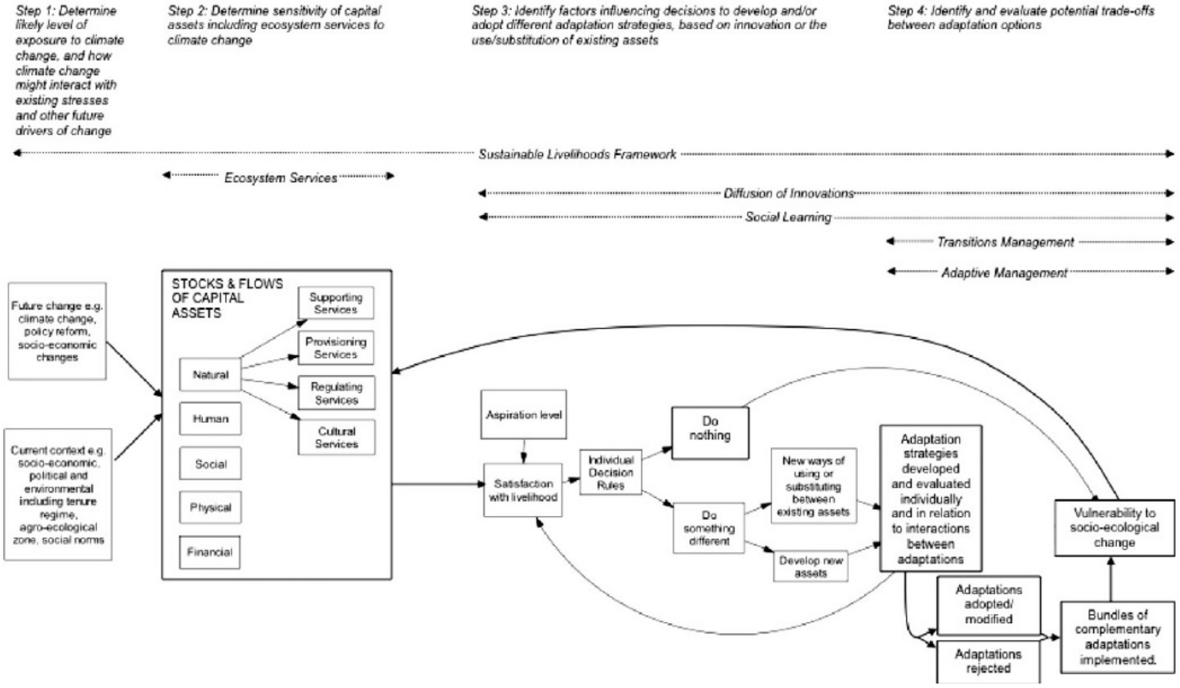

Figure A2.74 An integrated analytical framework for analysing livelihood vulnerability to climate change.

Source: Reed, M.S., Podesta, G., Fazey, I., Geeson, N., Hessel, R., Hubacek, K., et al (2013) Combining analytical frameworks to assess livelihood vulnerability to climate change and analyse adaptation options. *Ecological Economics*, 94:66–77
Figure 1, page 70. Open Access, published by Elsevier.

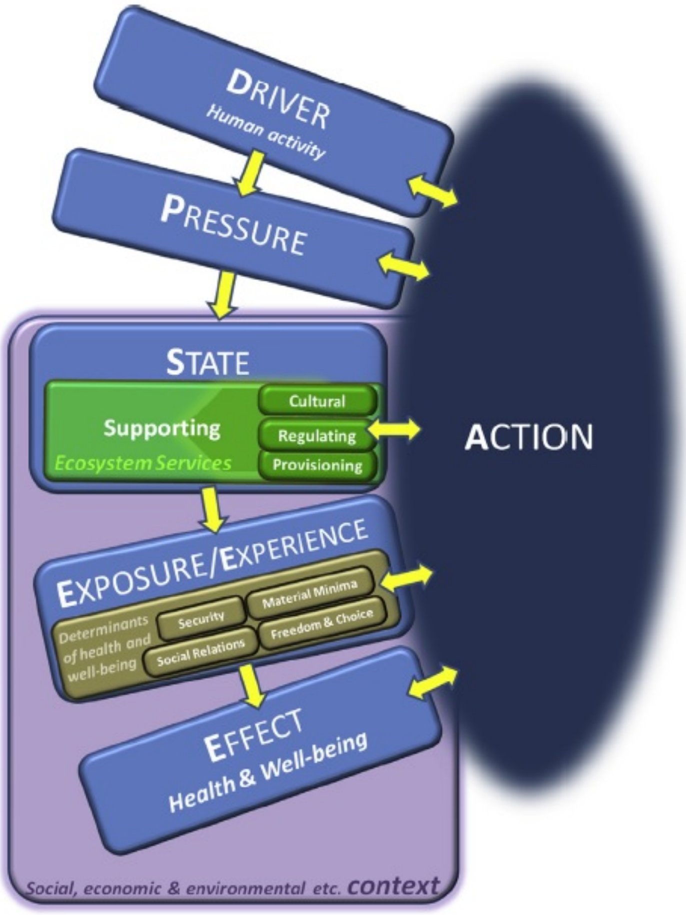

Figure A2.**75** Ecosystem-enriched DPSEEA (eDPSEEA)- a conceptual framework for an integrated assessment of human and ecosystem health and ecosystem service provision.

Source: Reis, S., Morris, G., Fleming, L.E., Beck, S., Taylor, T., White, M., Depledge, M.H., Steinle, S., Sabel, C.E., Hurley, F., et al. (2013) Integrating health and environmental impact analysis. *Public Health,* 1-7
Figure 1, page 5. Reproduced with permission from Elsevier

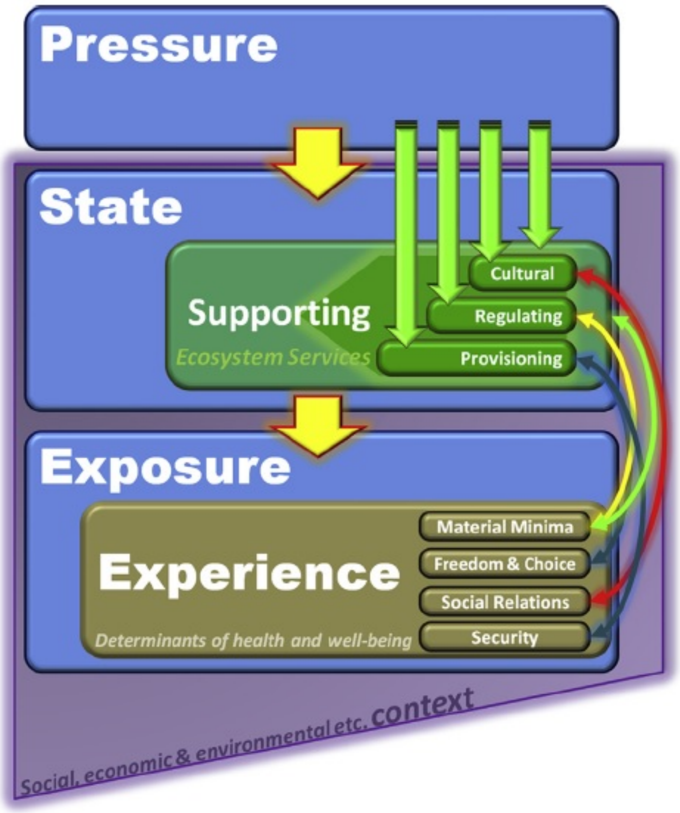

Figure A2.76 Illustrating the potential for feedback loops between Pressure, State and Exposure/ Experience which is manifest when considering relationships between ES and determinants of human health and well-being.

Source: Reis, S., Morris, G., Fleming, L.E., Beck, S., Taylor, T., White, M., Depledge, M.H., Steinle, S., Sabel, C.E., Hurley, F., et al. (2013) Integrating health and environmental impact analysis. *Public Health,* 1-7
Figure 2, page 5. Reproduced with permission from Elsevier

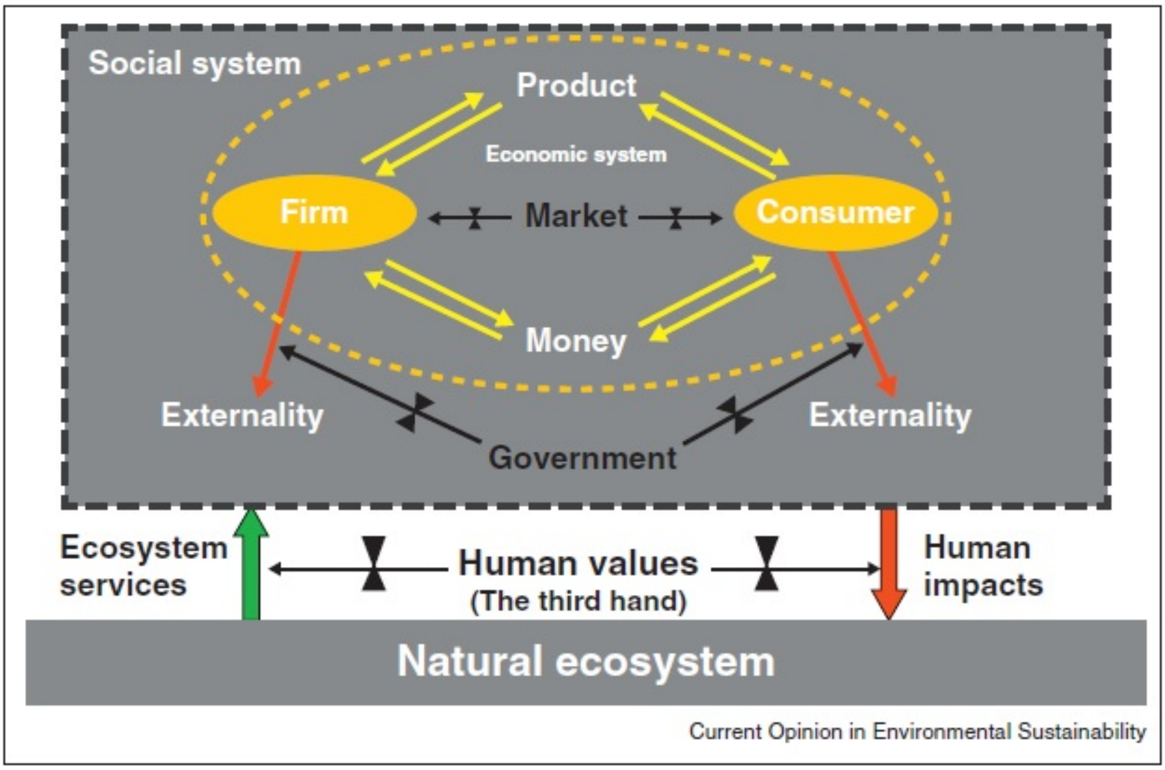

Figure A2.77 A framework of relationships between the natural ecosystem and the social and economic systems.

Source: Wang, S., Fu, B., Wei, Y. & Lyle, C. (2013) Ecosystem services management: an integrated approach. *Current Opinion in Environmental Sustainability,* 5:11-15
Figure 1, page 13. Reproduced with permission from Elsevier

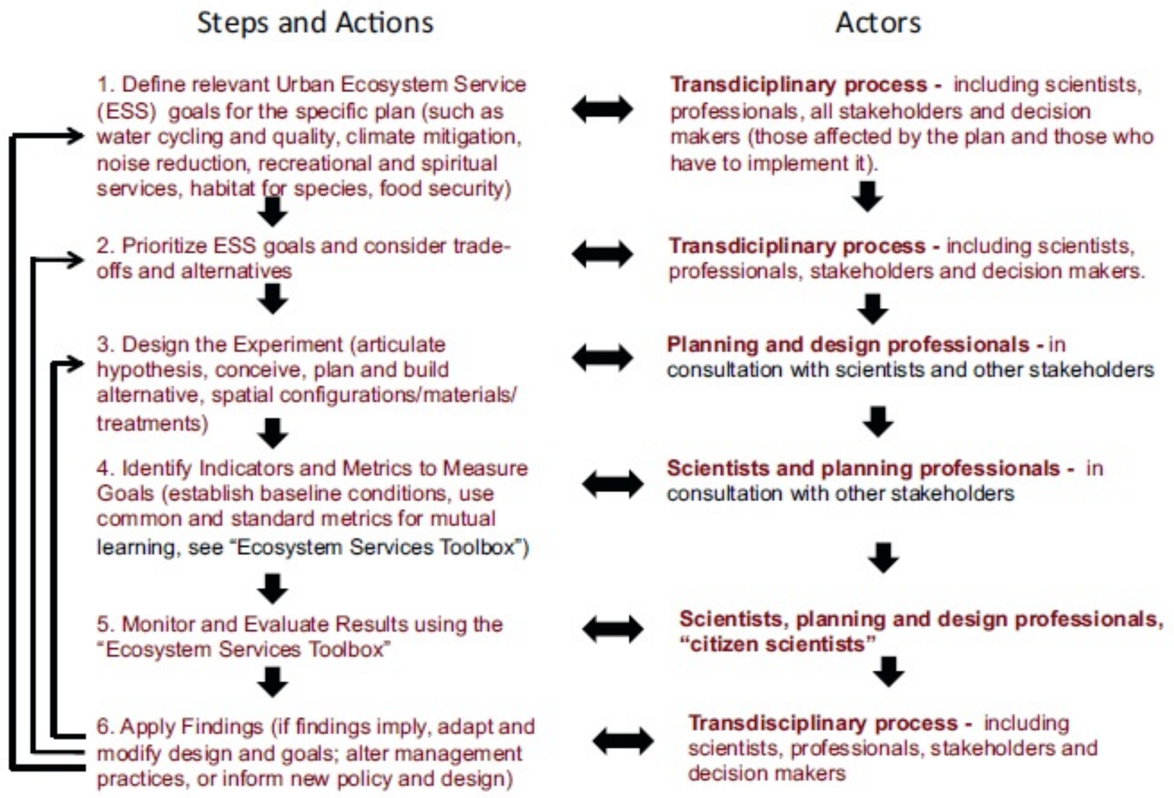

Figure A2.78 A transdisciplinary adaptive design and planning model.

Source: Ahern, J., Cillier, S. & Niemelä, J. (2014) The concept of ecosystem services in adaptive urban planning and design: A framework for supporting innovation. *Landscape and Urban Planning*, 125:254–259
Figure 1, page 256. Reproduced with permission from Elsevier

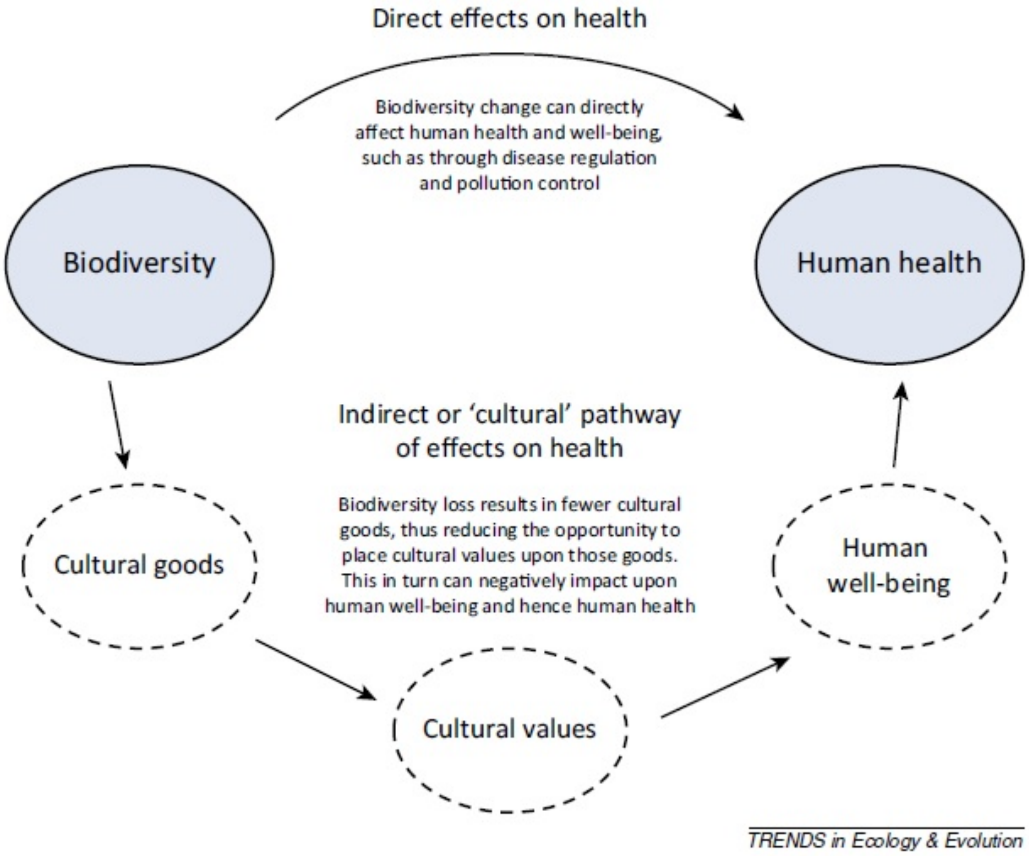

Figure A2.79 The direct and indirect (cultural) pathways from biodiversity to human health.

Source: Clark, N.E., Lovell, R., Wheeler, B.W., Higgens, S.L., Depledge, M.H. & Norris, K. (2014) Biodiversity, cultural pathways, and human health: a framework. *Trends in Ecology & Evolution*, 29:198-204
Figure 1, page 200. Open Access, published by Elsevier.

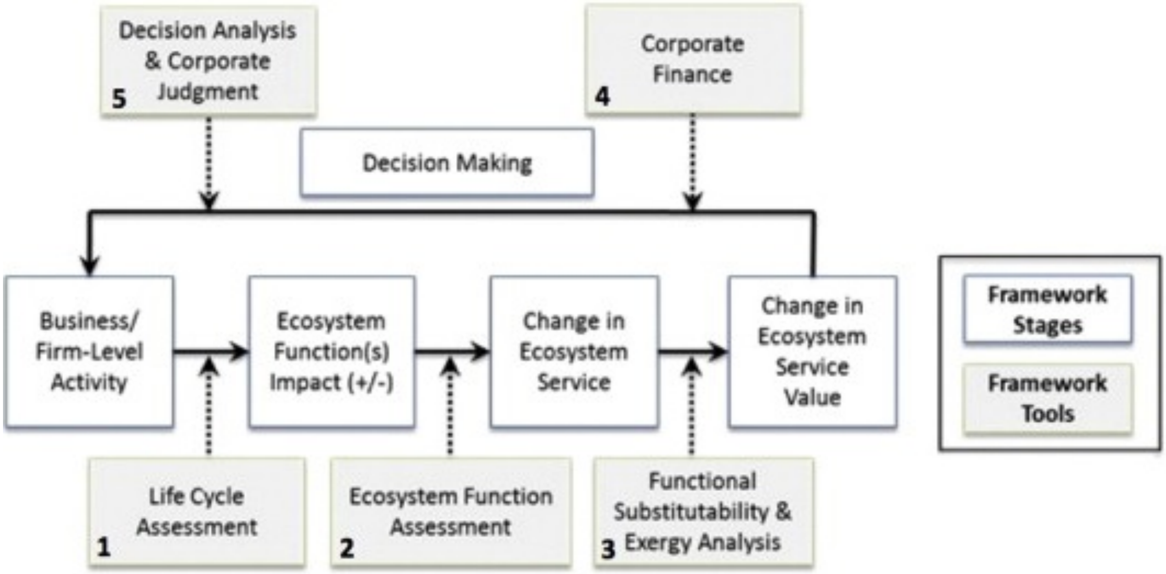

Figure A2.80 Firm-level ecosystem service valuation framework. Modified from Comello & Lepech (2011) and Comello et al. (2012).

Source: Comello, S.D., Maltais-Landry, G., Schwegler, B.R. & Lepech, M.S. (2014) Firm-level ecosystem service valuation using mechanistic biogeochemical modeling and functional substitutability. *Ecological Economics,* 100:63-73
Figure 1, page 64. Reproduced with permission from Elsevier.

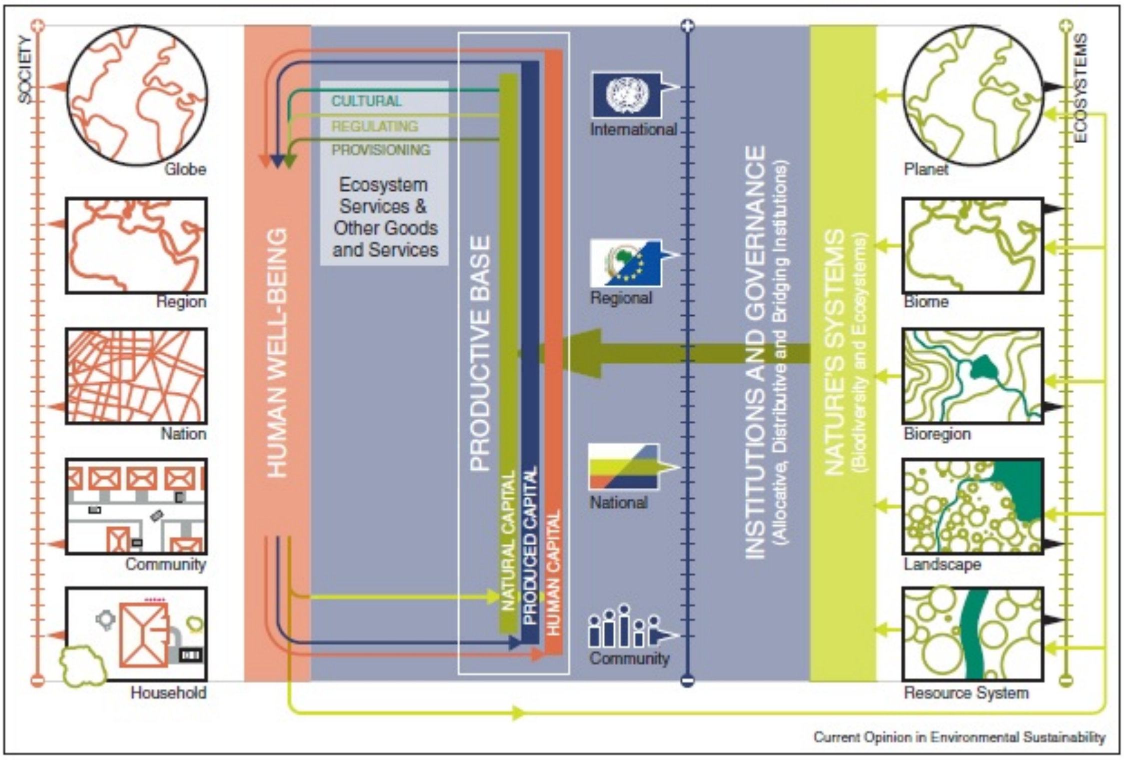

Figure A2.81 A multi-scale conceptual framework on nature, the productive base of societies and human well-being.

Source: Duraiappah, A.K., Asah, S.T., Brondizio, E.S., Kosoy, N., O'Farrell, P.J., Prieur-Richard A.H., et al. (2014) Managing the mismatches to provide ecosystem services for human well-being: a conceptual framework for understanding the New Commons. *Current Opinion in Environmental Sustainability,* 7:94–100
Figure 1, page 96. Reproduced with permission from Elsevier.

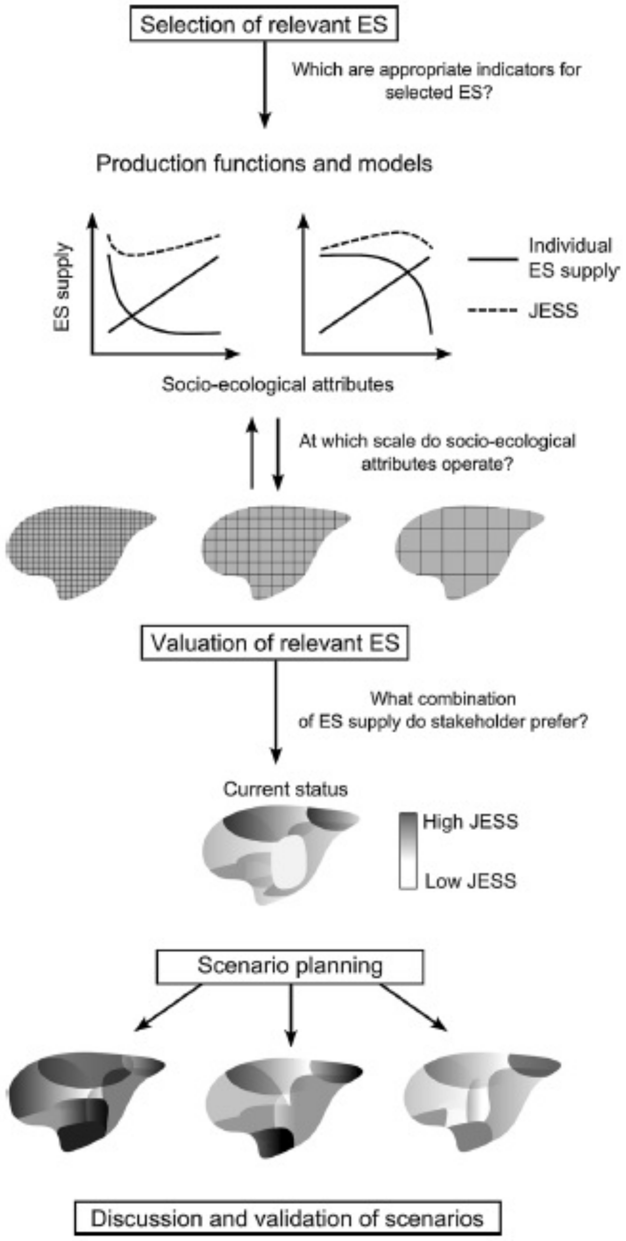

Figure A2.82 Methodological approach proposed to guide research for the assessment of a socially-relevant, process-based landscape multifunctionality, and thus to inform landscape planning aimed at improving local well-being. (Text in boxes indicates instances of stakeholders' participation. JESS =joint ecosystem service supply).

Source: Mastrangelo, M.E., Weyland, F., Villarino, S.H., Barral, M.P., Nahuelhual, L. & Laterra, P. (2014) Concepts and methods for landscape multifunctionality and a unifying framework based on ecosystem services. *Landscape Ecology,* 29:345–358
Figure 4, page 355. Reproduced with permission from Springer Netherlands

.

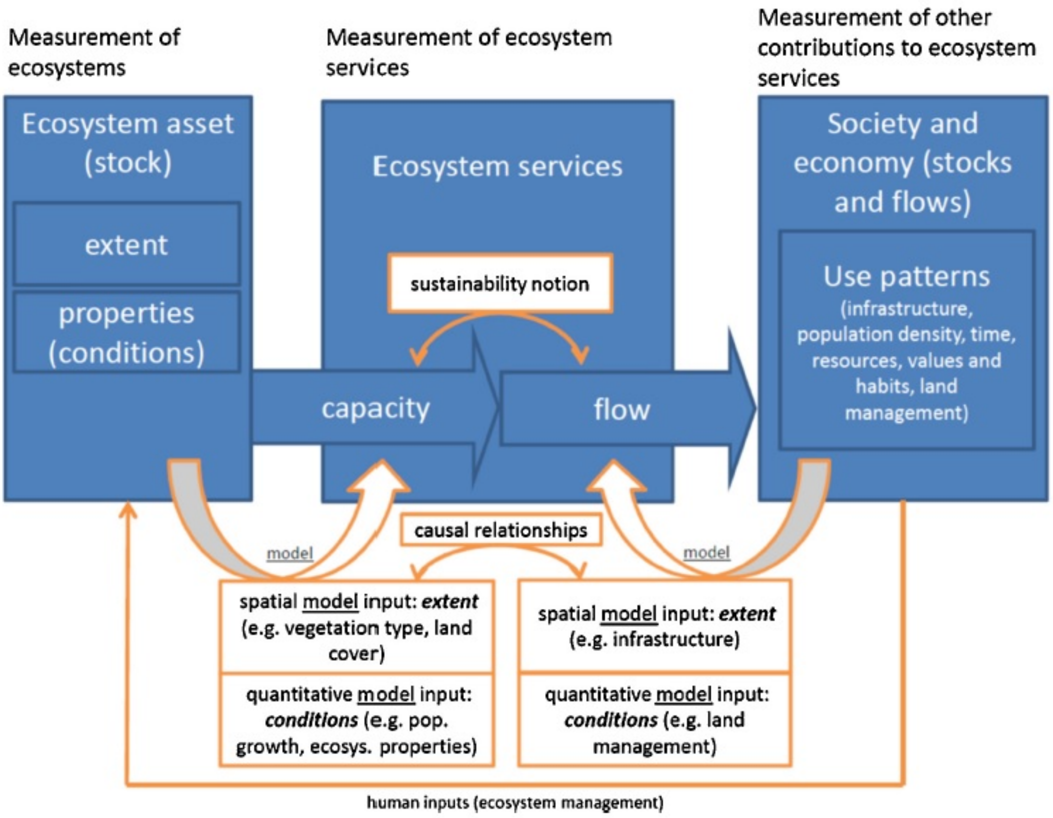

Figure A2.83 Integration of ES capacity and flow models in ecosystem accounting.

Source: Schröter, M., Barton, D.N., Remme, R.P. & Hein, L. (2014) Accounting for capacity and flow of ecosystem services: a conceptual model and a case study for Telemark, Norway. *Ecological Indicators,* 36:539– 551
Figure 1, page 541. Reproduced with permission from Elsevier

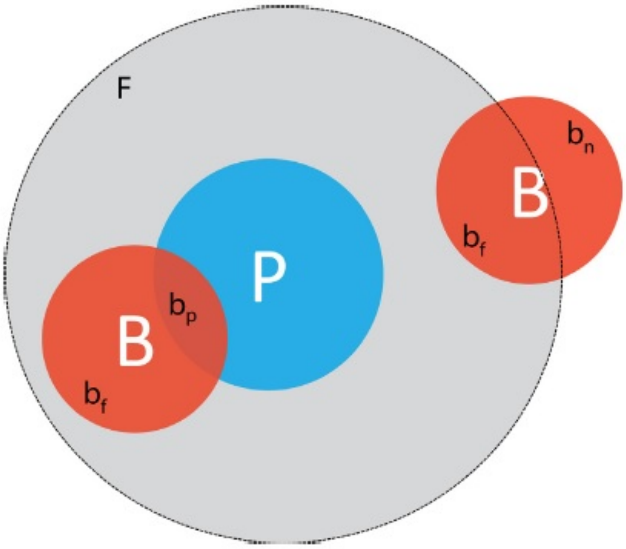

Figure A2.84 Framework to analyze and quantify ecosystem service flow (Red circles with B, represent benefiting areas, while blue circle with P represents provisioning areas. F is the flow area within which services from provisioning area can potentially be delivered; bf is the benefiting area not overlapping with P but within F; bn is the benefiting area not-overlapping with the provisioning area and outside F; bp is the benefiting area overlapping with the provisioning area)

Source: Serna-Chavez, H.M., Schulp , C.J.E., van Bodegom, P.M., Bouten, W., Verburg, P.H. & Davidson, M.D. (2014) A quantitative framework for assessing spatial flows of ecosystem services. *Ecological Indicators,* 39:24– 33
Figure 1, page 25. Reproduced with permission from Elsevier
