# Supplementary material for: Integrating Human and Ecosystem Health Through Ecosystem Services Frameworks
Source: Ecohealth. 2015 Sep 24;12:660–71. doi: 10.1007/s10393-015-1041-4 (PMC4700085; doi:10.1007/s10393-015-1041-4)
Supplement: Supplementary file 3 — Supplementary material 3 (PDF 141 kb) [file 10393_2015_1041_MOESM3_ESM.pdf]

Appendix 3: Analysis of key features in ecosystem services frameworks

Key feature analysis
(red= not represent, amber= partially represented, green= strongly represented)

Author	Year	Figure number (see Appendix 2)	Human health	Ecosystem health	Determinants/ drivers of human health	Determinants/ drivers of ecosystem health	Feedback mechanisms (between human health and ecosystem health)	Time	Space
De Groot	1987	A2.1	Not explicitly depicted. However ‘human needs and activities’ is included.	Not explicitly depicted. However ‘natural processes and components’ is included.	‘Natural processes and components’ are determinants of ‘human needs and activities.’	‘Human needs and activities’ are determinants of ‘natural processes and components.’	A two-way feedback mechanism of ‘functional interrelations’ is shown between ‘human needs and activities’ and ‘natural processes and components’.	Not represented	Not represented
Costanza & Daly	1992	A2.2	Not represented - only 'population' and ‘human capital’ and its decay.	Not explicitly depicted. However ‘renewable natural capital’, along with its 'decay' and 'growth and maintenance' (suggesting change in condition), are included. 'Ecosystem services' and 'ecosystem goods' are included.	N/A. Human health not represented (and there are no determinants of 'population').	‘Solar energy’ is a determinant of ‘renewable natural capital’. ‘Economic demand’ is a determinant of ‘ecosystem services' and 'ecosystem goods'.	N/A as there is no representation of human health. However, a complex network of feedback mechanisms are depicted between the other components, with interactions between human, manufactured and natural capital, economic and ecosystems goods and services, and economic demand.	Not represented	Not represented
Rapport et al	1998	A2.3	Represented as ‘human health risks’, which is a distinct component of the framework.	Represented as ‘changed ecosystem structure and function’, including decreased biodiversity and resilience, increased disease, change in community structure towards r-selected species, and eutrophication (i.e. change in condition to poor ecosystem health).	‘Human health risks’ are determined directly by ‘changed ecosystem structure and function’ and ‘decreased ecosystem services’ (these are ultimately determined by 'human pressures on ecosystems and landscapes’ such as harvesting, waste residuals, physical restructuring, magnified extreme events, and exotic species introductions)	‘Changed ecosystem structure and function’ are determined by ‘human pressures on ecosystems and landscapes’, such as harvesting, waste residuals, physical restructuring, magnified extreme events, and exotic species introductions.	There is a circular feedback mechanism between the components of the framework, thus linking 'human health risks' with 'changed ecosystem structure and function', via (amongst other components) ‘human society’s response’ (e.g. improved environmental management, decreased pressure on ecosystems and landscapes).	Not represented	Not represented
Scoones	1998	A2.4	‘Well-being and capabilities improved’ is included as a 'sustainable livelihoods outcome'.	Not explicitly included. However ‘natural resources base sustainability ensured’ is included as a 'sustainable livelihoods outcome'. 'Sustainability' may suggest an element of ecosystem health.	‘Well-being and capabilities improved' is determined by 'livelihood strategies', which in turn are affected by other factors including context (e.g. history, politics, macro-economic conditions, climate etc), resources (natural, economic/financial, social, human capital) and institutional processes.	‘Natural resources base sustainability’ is determined by 'livelihood strategies', which in turn are affected by other factors including context (e.g. history, politics, macro-economic conditions, climate etc), resources (natural, economic/financial, social, human capital) and institutional processes.	As ‘well-being and capabilities improved’ and ‘natural resources base sustainability ensured' are both listed under ‘sustainable livelihood outcomes’, the feedback between them is not clearly depicted. However, there is a two-way feedback mechanism between ‘context condition and trends’ with ‘sustainable livelihood outcomes’ (with additional factors, including resources, institutional processes and livelihood strategies, in between).	Not represented	Not represented
Leach et al	1999	A2.5	Not represented	Not represented. Only ‘environmental goods and services’ depicted in a generic sense.	N/A. Human health not represented.	‘Environmental goods and services’ are determined by ‘capabilities’ of ‘differentiated social actors’, which are also affected by 'endowments' and 'entitlements'. 'Institutions' also impact upon the system.	N/A as there is no representation of human health. However, there is a circular feedback mechanism between ‘environmental goods and services’ and ‘differentiated social actors’, and two-way interactions between institutions and endowments/ entitlements factors.	Not represented	Institutions are shown to be operating at different spatial scales (micro, meso, macro).
Perrings et al	2002	A2.6	Not represented	Partially represented (in terms of poor ecosystem health) as ‘degraded resources’, and ‘degraded energy, pollution’ in the ecological system.	N/A. Human health not represented.	‘Degraded resources’ and ‘degraded energy, pollution’ in the ecological system are caused by the ‘economic system’.	N/A as there is no representation of human health. However, there is some feedback between the ecological and economic system; the ecological system feeds into the economic system through ecological services, natural resources and energy, and the economic system impacts upon the ecological system through degradation of resources and energy, and pollution.	Not represented	Not represented
De Groot et al	2002	A2.7	Not represented. However, human health is discussed by the authors in relation to ‘socio-cultural value’ of ecosystem services and goods, both represented in the framework.	Not explicitly depicted. However, 'ecosystem structure and processes' are included, contributing to 'ecosystem functions'- which includes regulating functions (described by the authors in the text as contributing to ecosystem health).	N/A. Human health not represented.	‘Ecosystem structure and processes’ are determined by ‘decision-making processes to determine policy options and management measures’ (which are influenced by the values derived from ecosystem services).	N/A as there is no representation of human health. However, there is a circular feedback mechanism between the main components of the framework: ecosystem structure and processes, ecosystem functions, ecosystem services and goods, values and decision-making processes.	Not represented	Not represented
Ekins et al	2003	A2.8	‘Health’ is represented as one of three components of ‘human welfare’, which is a key component of the framework.	Elements of 'natural capital' and 'functions of natural capital' are included, the latter which are described as being 'responsible for ecosystem resilience'.	‘Human welfare’ (which includes human health) is determined by ‘functions for people’, which are derived from 'natural capital' (the latter of which is determined by 'influences' (social, economic, environmental, ethical)).	‘Natural capital’, which includes 'functions' (that are responsible for ecosystem resilience) are determined by ‘influences’ (including social, economic, environmental and ethical).	There is a circular feedback mechanism between ‘influences’, ‘natural capital’, ‘functions for people’ and ‘human welfare’.	Not represented	‘Importance of spatial scale’ is shown to influence ‘functions of natural capital’ and ‘functions for people’- but no more detail regarding spatial scale is shown.
Millennium Ecosystem Assessment (a)	2005	A2.9	‘Health', comprising of ‘strength’, ‘feeling well’ and ‘access to clean air and water’, is considered as one element of five core elements of ‘human well-being’, which is the key focus of the framework (in addition to ecosystem services).	Not explicitly depicted. However 'ecosystem services' are included and categorised, and include supporting services (such as nutrient cycling, soil formation, primary production), which relate to ecosystem functioning.	Ecosystem services' are portrayed as determinants of human health- particularly provisioning and regulating services, and to a lesser extent cultural services. Supporting services underpin these other ecosystem services. However, no additional external drivers shown.	Not represented - no determinants of 'ecosystem services' are shown.	Not present- only a one-directional relationship between ecosystem services and human well-being.	Not represented	Not represented (although ecosystem services are embedded within a broader domain of ‘life on earth-biodiversity’)
Millennium Ecosystem Assessment (a)	2005	A2.10	Health' is depicted as one of five components of ‘human well-being and poverty reduction’, which is a prominent focus of the framework.	Not explicitly depicted. However 'ecosystem services' are included and categorised, and include supporting services (e.g. soil formation and primary production), which relate to ecosystem functioning.	‘Human well-being and poverty reduction’ (which includes 'health') is determined by 'ecosystem services', 'direct drivers of change' (e.g. changes in local land use cover, species introduction or removal, etc.) and 'indirect drivers of change' (demographic, economic, socio-political, science & technology, cultural & religious).	'Ecosystem services' are influenced by ‘direct drivers of change’ (e.g. changes in local land use cover, species introduction or removal, technology adaption and use, climate change etc.) which in turn are affected by ‘indirect drivers of change’.	There is a circular feedback mechanism between the four main components of the framework, thus providing feedback between 'ecosystem services' and 'human well-being and poverty reduction'. Other two-way interactions and circular feedbacks are also depicted.	Time is represented through two arrows showing short-term and long-term scales.	Space is represented as an ‘axis’ of local, region and global scale. (‘Ecosystem services’ also sit within the broader ‘life on earth-biodiversity’)
Millennium Ecosystem Assessment (a)	2005	A2.11	'Health' is depicted as one of three components of 'human well-being' .	‘Changes in ecosystem condition’ (biophysical, chemical and biodiversity) are included (as well as 'changes in ecosystem services').	'Human well-being', which includes 'health', is determined by 'changes in ecosystem services', which are ultimately driven by 'endogenous and exogenous drivers.’	‘Changes in ecosystem condition’ are determined by ‘endogenous and exogenous drivers.’	Only one directional interactions are shown, starting with ‘endogenous and exogenous driver’ through to ‘human well-being’ and ‘evaluating trade-offs’.	Not represented	Not represented
Millennium Ecosystem Assessment (a)	2005	A2.12	'Health' is depicted as one of three components of 'human well-being', which is one of six main components of the framework.	Represented through 'ecosystem processes' which includes 'resilience' and 'resistance' as well as several processes (e.g. nutrient cycling). Components of 'biodiversity' (richness, evenness, composition, interactions) are also included.	'Human well-being', which includes 'health' is determined by 'ecosystem services' (which are determined by global changes -including climate, biogeochemical cycles, land use and species introductions- through their impacts on 'biodiversity')	'Ecosystem processes' which includes 'resilience' and 'resistance' are determined by 'biodiversity' which is impacted on by global changes (including climate, biogeochemical cycles, land use and species introductions)	Several circular feedback mechanism shown between different components (biodiversity, functional traits, ecosystem processes, ecosystem services, human well-being, and global changes)- thus 'health' and ecological 'resilience'/'resistance' feedback on one another.	Not represented	Not represented (although 'global changes' are recognised as impacting on biodiversity)
Millennium Ecosystem Assessment (b)	2005	A2.13	Human health is central to the framework, represented through potential negative impacts to health grouped into three categories- (i) direct health impacts (e.g. floods, heat waves, water shortage, landslides etc) (ii) ‘ecosystem-mediated’ health impacts (e.g. altered infectious disease risk, reduced food yields, depletion of natural medicine etc) (iii) indirect, deferred and displaced health impacts (e.g. diverse health consequences of livelihood loss, population displacement etc).	Partially represented (in terms of poor ecosystem health) through the component ‘environmental changes and ecosystem impairment’, for example ‘biodiversity loss’ and ‘freshwater depletion and contamination’ etc.	Human health is portrayed as being determined by ‘environmental changes and ecosystem impairment’ (such as ‘forest clearance and land cover change’, ‘urbanisation’ etc.) which in turn is driven by ‘escalating human pressure on the global environment’.	‘Environmental changes and ecosystem impairment' is driven by generic ‘escalating human pressure on the global environment’	Not present- only one way relationship between environmental changes/ecosystem impairment and health impacts	Not represented	Not represented
National Research Council	2005	A2.14	Not represented	Not explicitly depicted. However, ecosystem 'structure' and 'functions' are depicted, leading to ES.	N/A. Human health not represented.	The 'Ecosystem' (including its 'structure' and 'functions') is determined by ‘human actions (private/public)’	N/A as there is no representation of human health. However, there is a circular feedback mechanism between the 'ecosystem' and 'values', linked by 'ecosystem good and services' and 'human actions'.	Not represented	Not represented
Hein et al	2006	A2.15	Not represented	Not represented. However, the 'ecosystem' is depicted along with 'ecosystem services' , which are categorised, but do not including supporting services (which are more closely aligned with ecosystem health).	N/A. Human health not represented.	N/A Ecosystem health not represented. No determinants of 'ecosystem' shown.	N/A as there is no representation of human or ecosystem health. Only one-directional relationships shown.	Not represented	Not represented
Chapin et al	2006	A2.16	Not represented (although does include a number of other social features)	Not explicitly represented. However a number of ecological components and processes are depicted in the ‘ecological subsystem’ component, e.g. soil disturbance, soil nitrate, functional types etc.	N/A. Human health not represented.	The 'ecological subsystem', which includes various ecological processes, is determined by ‘exogenous controls’, the 'globe' and 'human actors' (with influence of 'institutional responses')	N/A as there is no representation of human health. However, via institutional responses, there are circular feedback mechanisms between ‘human actors’ with both slow and fast variables in both the ecological and social subsystem, which in turn impact upon human actors.	Temporal scale is depicted, including slow variables (relatively constant over years to decades) and fast variables (change on daily, seasonal and interannual timescales)	Spatial scale is depicted, from global and exogenous controls, to more local variables, with examples/detail provided for each level.
Chapin et al	2006	A2.17	Not represented	Not represented. However, the 'ecological subsystem' is categorised into different ecosystem goods/ services, but this does not include supporting services (which are more closely aligned with ecosystem health).	N/A. Human health not represented.	Ecosystem health not represented, however, the 'ecological subsystem', consisting of ecosystem services, is influenced by 'human actors' via different types of institutions.	N/A as there is no representation of human health. However, via institutional responses, there are circular feedback mechanisms between ‘human actors’ with the ecological and social subsystem, which in turn impact upon human actors.	Not represented	Not represented
Brauman et al	2007	A2.18	Not represented	Not explicitly represented. However, ‘biophysical generation of ES’ is included and one policy question included is ‘how does service delivery relate to the condition of an ecosystem?’ - both of which relate to ecosystem health.	N/A. Human health not represented.	‘Biophysical generation of ecosystem services’ and 'trends in ecosystem services generation' is influenced by ‘policy’ via ‘protection and management’.	N/A as there is no representation of human health. However, there is a circular feedback mechanism between the ‘Biophysical generation of ecosystem services’ and 'trends in ecosystem services generation', 'beneficiaries and producers', 'valuation', and 'policy'.	Not explicitly represented, however, one policy question depicted is ‘what services are produced by various ecosystems and at what spatial and temporal scales?’	Not explicitly represented, however, policy question depicted include ‘what services are produced by various ecosystems and at what spatial and temporal scales?’ and ‘what is the spatial relationship between ecosystem service supply and consumption?'
Wallace	2007	A2.19	Not represented	Not explicitly depicted. However, ‘structure and composition of ecosystem’ is included, as well as 'ecosystem processes redistribute matter and energy amongst assets, or evolve new assets', which includes various supporting services.	N/A. Human health not represented.	‘Structure and composition of ecosystem’ leads to 'ecosystem processes redistribute matter and energy amongst assets, or evolve new assets' which determines a 'new structure and composition of ecosystem'.	N/A as there is no representation of human health. However, there is a temporal feedback mechanism between ‘structure and composition of ecosystem’ and 'ecosystem processes redistribute matter and energy'.	Time is a key component, with two points of time represented, whereby the ‘structure and composition of ecosystem’ alters to a new structure at a later point in time.	Not represented
Wallace	2007	A2.20	Not represented	Not represented. However, various biotic and abiotic components/processes of ecosystems are included (e.g. photosynthesis, pollination, biomass production, soil/nutrient formation etc).	N/A. Human health not represented.	N/A Ecosystem health not represented. However, 'Sunlight, rainfall, soil/nutrient formation, and atmosphere' are shown as determinants of ecosystem service deliver.	N/A as there is no representation of human or ecosystem health. Only one-directional interactions, showing an ecosystem pathway, resulting in ecosystem services/goods.	Not represented	Not represented
Cowling et al	2008	A2.21	Not represented	Represented as an axis of ‘status of social-ecological system’, from vulnerable to resilient.	N/A. Human health not represented.	‘Status of social-ecological system’ is determined by the project phase - ranging from vulnerable at the assessment stage, through to resilient at the management implementation stage.	N/A as there is no representation of human health. There is circular feedback from management implementation (which includes adaptive management), to earlier project phases.	Not represented.	Spatial scale represented as one axis - from regional to local- and this is shown to correspond with different stages for safeguarding ecosystem services.
Fisher et al	2008	A2.22	Not represented	Not explicitly depicted. However ecosystem services are categorised, and include 'intermediate services' e.g. soil formation, nutrient cycling, which relate to ecosystem functioning.	N/A. Human health not represented.	Not represented- no determinants of intermediate ecosystem services shown.	N/A as there is no representation of human health. Only one-directional relationship between intermediate services, final services and benefits, with the input of other capital.	Not represented	Not represented
Loring et al	2008	A2.23	‘Health’ is depicted in a minor way as a one of four 'service outcomes'	Not represented- only ‘services’ (as in ecosystem services) shown.	'Health', as a 'service outcome', is determined by 'consumer and provider behaviour' (which in turn is determined by 'service execution context').	N/A Ecosystem health not represented.- and no determinants of ‘services’ shown	N/A as there is no representation of ecosystem health, and no feedback from 'health' to 'service'. Only one-directional relationships shown between different service features and consumer/provider behaviour	Not represented	Not represented

Turner & Daily	2008	A2.24	Not represented	Not explicitly depicted. However, 'ecosystem functions' are shown, along with ecosystem services (including intermediate services).	N/A. Human health not represented.	'Ecosystem services' (which link to 'ecosystem functions') are determined by 'environmental change process'.	N/A as there is no representation of ecosystem health. However, there are circular and cross-directional interactions between decision-making/management processes, governance, ecosystem services and environmental change processes.	Temporal scale is mentioned- 'ecosystem scales (spatial/temporal)' and 'marginal changes in services provision across spatial and temporal scales'.	Spatial scale is mentioned- 'ecosystem scales (spatial/temporal)' and 'marginal changes in services provision across spatial and temporal scales'.
Carpenter et al	2009	A2.25	'Individual well-being' and 'community well-being' is depicted, as part of a cycle of local feedbacks	Not represented. Only 'ecosystems and services' in a generic sense at different scales.	'Individual well-being' and 'community well-being' is determined by 'local ecosystems and services' and 'local governance', as well as the overall system (i.e. Governance and ecosystems at local to global level).	Ecosystem health not represented, however 'ecosystems and services' are affected by governance at multiple scales.	Circular feedback loop/ two-way interactions shown at a local scale between 'community well-being', 'individual well-being', 'local ecosystems and services' and 'local governance'. Multi-scale feedback also shown between 'ecosystems and services' and 'governance'.	Not represented	Spatial scale is a prominent feature, depicted from local ecosystem and governance, through to regional then global. Also includes local, regional and global feedbacks.
Daily et al	2009	A2.26	Not represented	Not explicitly depicted. However models of ecosystem services are categorised, and include 'supporting services' as well as aspects of 'biodiversity' - 'species' and 'habitats'.	N/A. Human health not represented.	Determinants of ecosystem services not shown, only the stages that precede the modelling of ecosystem services.	N/A as there is no representation of human health. Only one directional interaction between decision-making stages	Not represented	Not represented
Daily et al	2009	A2.27	Not represented	Not represented - just 'ecosystems' and 'services' in a generic sense.	N/A. Human health not represented.	'Ecosystems' are determined by 'decisions' via 'actions and scenarios' (and 'decisions' are determined by 'institutions' via 'incentives')	N/A as there is no representation of human health. However, there is a circular feedback mechanism shown between different decision-making stages (decisions, ecosystems, services, values and institutions)	Not represented	Not represented
Fisher et al	2009	A2.28	Not represented	Not explicitly depicted. However ecosystem services are categorised and include 'intermediate services' e.g. soil formation, water regulation, which are generated through interactions in 'structure and processes'.	N/A. Human health not represented.	No determinants of 'intermediate services' (or specifically 'structure and processes') shown.	N/A as there is no representation of human health. Only one-directional relationship between intermediate services, final services and benefits, with the input of other capital.	Not represented	Not represented
Fisher et al	2009	A2.29	Not represented	Not represented- only ecosystem service production and benefit areas.	N/A. Human health not represented.	N/A Ecosystem health not represented and no determinants of ecosystem service production areas shown.	N/A as there is no representation of human health. Only one directional relationship between ecosystem service production areas and benefit areas.	Not represented	Spatial dimension is the main feature of the framework- it depicts spatial relationships between ecosystem service production areas and service benefit areas using four different potential landscape scenarios.
McLeod & Leslie	2009	A2.30	Not represented	Not represented- just 'ecosystems services', 'ecosystems' and 'ecological domain' in a generic sense	N/A. Human health not represented.	'Ecosystem services', and the 'ecosystems' within the 'ecological domain', are determined by the 'social domain'.	N/A as there is no representation of human health. However, two-way feedback mechanisms are depicted between ES and the social and ecological domains.	Not represented	Multiple spatial scales are a prominent feature, and are depicted for the social domain- individuals, institutions, culture- and the ecological domain – local ecosystems, regional seascapes and large marine ecosystems, although no further detail provided.
Robinson et al	2009	A2.31	Not represented	Not represented - just 'ecosystem natural capital' and 'ecosystem services' at a generic level.	N/A. Human health not represented.	Within the 'regional scale', 'ecosystem natural capital' is determined by 'regional decision-making' via 'human action'. The 'regional scale' is also determined by the 'national scale' including economic activity, national regulatory/ governance/ policy system. Similarly it is also determined by the 'international scale- consequences for the earth system', including 'global and regional land use & cover: global and regional climate'.	N/A as there is no representation of human health. However, there is circular feedback between ecosystem natural capital, ecosystem services and valuation and regional decision-making. There is also two-way feedback between the different spatial scales.	Not represented	Spatial scale is a prominent feature- the framework is split into regional, national and international scale, with particular reference to information transfer between them.
Bryan et al	2010	A2.32	Not represented	Not explicitly depicted. However ecosystem services are included and categorised, and include 'supporting services', which relate to ecosystem functioning.	N/A. Human health not represented.	No determinants of ecosystem services shown.	N/A as there is no representation of human health, and there are no feedback mechanisms shown.	Not represented	Not represented
Collins et al	2010	A2.33	'Health' (and also 'quality of life') represented as a 'human outcome' within the 'social template'.	'Community structure' (including 'species turnover time, trophic structure, microbial diversity') and 'ecosystem function' (including 'flux, transport, storage, transformation, stoichiometry, primary productivity) are shown to interact, within the 'biophysical template'.	External drivers (climate and globalisation)' and 'ecosystem services', are determinants of the 'social template' which includes 'health'. Within the 'social template', 'human outcomes' (which includes 'health') is determined by 'human behavior' (which consists of 'policy, markets, reproduction and migration').	The 'biophysical template' (which consists of 'community structure' and 'ecosystem function') is determined by external drivers (climate and globalisation)' as well as pulse events (e.g. fire, drought, storms, dust events etc) and press events (e.g. climate change, nutrient loading, sea-level rise etc). These in turn are determined by the 'social template'.	There is a circular feedback mechanism between the 'social template' (which includes 'health') and the 'biophysical template' (which consists of 'community structure' and 'ecosystem function'), linked by 'ecosystem services' and pulse/press events.	Not represented	Not represented
De Groot et al (a)	2010	A2.34	Not represented	Not explicitly depicted. However, 'ecosystem and landscape properties' and 'ecosystem and landscape functions (incl. goods and services)' are shown.	N/A. Human health not represented.	Ecosystem and landscape functions (incl. goods and services) are determined by 'ecosystem and landscape properties' which are determined by 'management instruments' and 'financing mechanisms'.	N/A as there is no representation of human health. However, a circular feedback is shown between the key components, from understanding ecosystems and landscape properties, to valuing, trade-off analysis, planning and management to financial mechanism, leading back to ecosystem properties.	Not represented	Not represented
De Groot et al (b)	2010	A2.35	'Human well-being' is represented as a major component, and includes 'contribution to human health etc.' under 'benefits.'	Within 'ecosystems & biodiversity', 'biophysical structure or process (e.g. vegetation cover or Net Primary Productivity)' and 'function (e.g.slow water passage, biomass)' are shown leading to 'service'.	'Human well-being' is determined by 'service' derived from 'ecosystems & biodiversity'. There is also influence from 'institutions & human judgments' ' via 'feedback between value perception and use of ecosystem services'.	'Biophysical structure or process' and 'function' (within 'ecosystems & biodiversity') is determined by 'institutions and human judgements' via 'management/restoration'	There is a circular feedback loop between 'ecosystems & biodiversity' and 'human well-being', via 'service' (as in ecosystem service provision), and 'institutions & human judgments'.	Not represented	Not represented
De Groot et al (b)	2010	A2.36	'Human well-being' is a prominent component, consisting of economic, social and ecological dimensions.	Ecological structures or processes' (e.g. photosynthesis, nutrient cycling, food-chain dynamics etc) and 'functions' (production, regulation, habitat, information) is included, leading to 'services'.	'Human well-being' is determined 'service' from the 'ecosystems and biodiversity' component (which in turn is determined by direct, indirect and external drivers).	'Ecological structures or processes' and 'functions' is determined by 'direct drivers' (e.g. land use (change), habitat destruction, pollution & disturbance, resource use etc) and 'external drivers' (e.g. climate change). 'Direct drivers' are determined by 'indirect drivers' (e.g. demography, economy, technology etc).	There is a circular feedback mechanism between 'human well-being' and the 'ecosystems & biodiversity' component, via 'services' provision, and 'governance and decision-making' and indirect/direct/external drivers.	Not represented	Only weakly represented through recognition of 'international policy-makers' and 'local policy-makers'.
Feld et al	2010	A2.37	Not represented	Components of 'biodiversity' are shown, including 'abiotic/biotic, genetic, structural, functional', as well as 'functions/processes' relating to 'ecosystem services'.	N/A. Human health not represented.	No determinants of 'biodiversity' or 'ecosystem services' shown.	N/A as there is no representation of human health. However, there are feedback mechanisms (interdependency) between the different criteria for indicator suitability.	Not represented	The appropriateness of the spatial scale ('spatial scale appropriate?') of the ES indicator is included as a key criteria, as well as scalability ('indicator up/down-scalable?')
Haines-Young & Potschin	2010	A2.38	Not represented	Landscape structure or process (e.g. woodland habitat, net primary productivity') and 'functions (capacities) (e.g. slow passage of water, biomass)', leading to 'service (flows)', are included.	N/A. Human health not represented.	Landscape structure or process' is influenced by 'pressures'.	N/A as there is no representation of human health. However, there is circular feedback between involving values, leading to pressures, which may be influenced by policy decisions, impacting upon 'biophysical structure or process' which leads back through to values.	Not represented	Not represented
Haslett et al	2010	A2.39	Not explicitly represented - only 'societal needs'	Represented as 'ecosystem sustainability and integrity'	'Societal needs' are determined by 'management for sustainable ecosystem services'	'Ecosystem sustainability and integrity' is determined by 'sectoral policy management', and also elements of traditional conservation, including 'static site-based PAs and networks' and 'conservation policy and management strategy'.	There are circular feedback mechanisms within the traditional conservation strategy loop and the wider the wider societal and ES loop, the latter of which links 'societal needs' with 'ecosystem sustainability and integrity'. The two spheres also interact through other one-way/two-way feedback mechanisms.	Not represented	Not represented
Klug & Jennewien	2010	A2.40	Not represented	Not explicitly depicted. However ecosystem services are included and categorised, and include 'supporting services' and 'preserving services', which relate to ecosystem functioning.	N/A. Human health not represented.	No determinants of ecosystem services shown.	N/A as there is no representation of human health, and no feedback mechanisms shown.	Not represented	Not represented
López-Hoffman et al	2010	A2.41	'Society, human well-being' is depicted as a key component of a cycle.	Not explicitly depicted. However 'ecosystem functions' are included.	'Society, human well-being' is impacted upon by 'ecosystem services' and 'indirect drivers'. 'Transboundary interventions' also affect the overall system.	'Ecosystem functions' are affected by 'direct drivers' which in turn are influenced by 'indirect drivers'. 'Transboundary interventions' also affect the overall system.	There is a circular feedback mechanism linking 'society, human well-being' with 'ecosystem function', via 'ecosystem services' and indirect/direct drivers. There is also a circular feedback mechanism between different spatial localities.	Not represented	The spatial dimension is a key feature, showing relationship between ES/human well-being in one part of the globe with another, linked by 'transboundary interventions', within a global 'shared environment'.
Maynard et al	2010	A2.42	Represented as a minor component, as 'ecosystem services benefits' are depicted as 'constituents of well-being'.	Not explicitly depicted. However, 'ecosystem functions' are included.	Ecosystem services benefits (constituents of well-being) are determined by 'ecosystem services'	'Ecosystem functions' are determined by 'geographic location'.	Not represented – only one directional relationships from geographic location through to values.	Not represented	Represented only in the sense of 'geographic location' being a component.
Paetzold et al	2010	A2.43	Not represented	Not represented, only the concept of 'sustained provision of ecosystem services'.	N/A. Human health not represented.	N/A Ecosystem health not represented	N/A as there is no representation of human or ecosystem health. Only one-directional connections between process steps.	Not represented	Not represented
Potschin and Haines-Young	2010	A2.44	Represented in a minor way under 'benefit' – which includes 'aspects of well-being such as health and safety'.	'Biophysical structure or process (e.g. woodland habitat, net primary productivity') and 'function' (e.g. slow passage of water, biomass)', leading to 'service' are included.	'Benefit', which includes human health, is produced by 'service', derived from 'biophysical structure or process'.	'Biophysical structure or process' is determined by 'pressures'.	There is circular feedback between 'biophysical structure or process' and 'benefit' (which includes health)- via 'function', 'service', 'value' and 'pressures'.	Not represented	Not represented
Rounsevell et al	2010	A2.45	Not represented	Not represented. However 'states' which includes the 'supporting system' is included, and 'ecosystem service providers' at a general level.	N/A. Human health not represented.	'States' which includes the 'supporting system' is determined by 'pressures' (e.g. climate change, land use change, invasive species, air pollution). This is determined by 'drivers' (e.g. economy, demography, society, technology) and 'responses' (policy, strategic decisions and management).	N/A as there is no representation of human health. However, there is a circular and cross-directional feedback mechanism between drivers, pressures, states, impacts and responses.	Not explicitly represented, although 'baseline/ futures' suggests a temporal element.	Not represented
Schreckenberg et al	2010	A2.46	'Health' is included as one of three assets within the 'human' asset category.	Not explicitly represented. However, 'natural' assets are depicted, including 'supporting services' which relate to ecosystem functioning.	Not represented- no determinants of 'health' depicted.	Not represented - no determinants of 'natural' assets depicted.	Not represented- No feedback mechanisms shown	Not represented	Not represented
White et al	2010	A2.47	Social development and well-being' is depicted as a key feature.	Ecosystem function', and its relationship with 'biodiversity' (via 'ecological processes'), as well as 'physico-chemical environment', are shown.	The 'social development and well-being' sub-system is determined by the ecosystem services sub-system via 'input' (and influence of 'governance & equity). 'Input' is also determined (within the social sub-system) by 'legislation, incentive and technological development'.	'Ecosystem function' (which is determined directly by 'biodiversity' and the 'physico-chemical environment') is driven by earth/atmospheric processes and influenced by 'direct & indirect inputs (e.g. volcanoes, earthquakes)' in the ecological system, 'consumptive use' of ecosystem services. and 'direct and indirect impacts (e.g. climate change, sea level rise, contamination)' stemming from the outputs of the social system.	There is a circular feedback mechanism between 'Social development and well-being' and the ecological sub-system (which includes 'ecosystem function') via various stages including ecosystem services and 'outputs' (via impacts). There are further feedback mechanisms between various components of the framework.	Not represented	Not represented
Atkins et al	2011	A2.48	Not represented	Biota-ecological structure (e.g. diversity)', 'physico-chemical (e.g. waves, sediments)', and 'biota-ecological functioning (e.g. predator-prey)' are shown to be interacting with each other.	N/A. Human health not represented.	'Biota-ecological structure' determines ecosystem functioning, but no external determinants shown. However, the DPSIR framework includes 'drivers' and 'pressures' in a generic fashion.	N/A as there is no representation of human health. However, there is a circular feedback loop within the components of the DPSIR framework, and two-way interactions between the component of the 'ecosystem services and societal benefits' systems.	Not represented	Not represented
Balmford et al	2011	A2.49	Not represented	Not represented - only the notion of 'biodiversity and ecosystems loss' at a general level.	N/A. Human health not represented.	'Policy actions' influences the 'causes of biodiversity and ecosystems loss'	N/A as there is no representation of human health. However, there is a circular feedback mechanism between the five components of the framework, whereby quantification/ mapping of biodiversity/ecosystem loss results in policy actions with affects the drivers of biodiversity/ecosystem loss.	Not represented	Not represented

Balmford et al	2011	A2.50	'Physical health' is depicted as a category of ecosystem benefits, and includes 'synthetic medicines, cultivated medicines, medicines from wild species, avoidance of injury, avoidance of pollution, avoidance of infection and physical exercise'. Similarly 'psychological wellbeing' is also depicted as type of ecosystem benefit, and includes 'tourism, recreation, spiritual/cultural wellbeing, aesthetic benefits, nature watching, pets and garden plants'.	Not explicitly represented, however, 'core ecosystem processes' are shown (including production, decomposition, ecological interactions, evolutionary processes etc).	'Physical health' and 'psychological wellbeing' are determined by beneficial ecosystem processes, which are produced by core ecosystem processes.	Not represented- no determinants of 'core ecosystem processes' shown.	Not represented- only one-directional interaction between the three components of the framework.	Not represented	Not represented
Comello & Lepech	2011	A2.51	Not represented	Not explicitly represented, however 'ecosystem function impacts' is included at a generic level.	N/A. Human health not represented.	'Ecosystem functions impacts' are affected by 'business/ firm-level activity'	N/A as there is no representation of human health. However, a circular feedback mechanism shown between the different components, linked at the end by 'decision analysis' which in turn affects 'business/ firm-level activity'.	Not represented	Not represented
Daw et al	2011	A2.52	'Human well-being' is depicted as the key outcome.	Not represented, only the generic concept of ecosystem services	'Human well-being' is determined by ecosystem service provision.	Not represented - no determinants of ecosystem services shown.	Not represented- only one-directional relationships shown (although other feedbacks exist)	Not represented	Not represented
Daw et al	2011	A2.53	Not depicted clearly in the framework itself, however well-being of the different beneficiaries is highly relevant to the framework.	Not represented, only the generic concept of ecosystem services	The beneficiaries (and therefore their well-being) are affected by ecosystem services.	Not represented- no determinants of ecosystem services shown.	Not represented - only one-directional relationships shown between the beneficiaries and ecosystem services (although other feedbacks exist)	Not represented	Not represented
Kumar et al	2011	A2.54	'Health' is included as a key livelihood outcome.	'Ecological character' is depicted, consisting of 'processes', 'services' and 'components'.	'Health' (as a 'livelihood outcome'), is determined by 'livelihood strategies' 'ecosystem settings' (which is determined by direct and indirect drivers of change)	'Ecological character' is determined by the 'vulnerability context', consisting of direct and indirect drivers of change.	There is a two-way feedback mechanism between the 'ecosystem settings' (which includes processes and components) with 'livelihood outcomes' (which includes 'health') via 'livelihood strategies'. There are also additional two-way feedbacks between the 'ecosystem settings' and 'vulnerability context'.	Not represented	Space is represented as an 'axis' of local, region and global scale.
UK National Ecosystem Assessment	2011	A2.55	'Health value' is shown as one of three key values relating to 'human well-being'.	Not represented – only 'ecosystems, 'ecosystem services' and 'air, land, water and all living things'	'Human well-being' (including 'health values) is determined by 'ecosystems/'ecosystem services' and 'goods'. The 'ecosystems' are in turn determined by 'drivers of change'	'Ecosystems' are determined by 'drivers of change (direct and indirect)' (e.g. demographic, economic, socio-political, management practices etc.).	There is a circular feedback loop which links 'human well-being' with 'ecosystems'- via ' goods' and 'drivers of change'	Not explicitly represented, however a temporal dimension is suggested through 'future scenarios for the UK'.	Not represented (although ecosystems are placed within the broader 'air, water, land and all living things')
UK National Ecosystem Assessment	2011	A2.56	'Health' is represented as one of three key values relating to 'well-being value'	Not explicitly represented. However ecosystem services are categorised to include 'ecosystem processes/intermediate services' e.g. primary production, water cycling, soil formation, nutrient cycling, decomposition, weathering, ecological interactions, evolutionary processes.	'Health' value is determined by 'goods' derived from 'final ecosystem services'.	Not represented - no determinants of 'ecological processes/intermediate services' shown.	Not represented - only one-directional relationship between 'ecological processes/ intermediate service's and 'well-being values', which are shown to affect 'people'.	Not represented	Not represented.
Wainger & Mazzotta	2011	A2.57	Not represented	Represented as 'change in ecosystem stressor or condition'.	N/A. Human health not represented.	'Change in ecosystem stressor or condition' is determined by 'change in human actions (management opportunity)'.	N/A as there is no representation of human health. Only one-directional relationship from human actions through to social benefits.	Not explicitly depicted, although change in state (which is temporal) is a key feature.	Not represented.
Bastian et al (a)	2012	A2.58	Not represented	Within 'properties of ecosystems & landscapes', several processes/components are included: 'Structures, components/processes (functioning) and fluxes/ types/ interactions among spatial elements/ dynamics, change'.	N/A. Human health not represented.	'Properties of ecosystems & landscapes' are determined by generic 'driving forces', but also interact with the other pillars of the framework (services and potentials).	N/A as there is no representation of human health. However, there are two-way feedback mechanisms between the all main components of the framework (properties, potentials, services and driving forces)	Represented only weakly within the properties pillar, as 'dynamics, changes' - referred to in the text as having a temporal dimension.	Represented only weakly within the properties pillar, as 'interactions with spatial elements'.
Bastian et al (b)	2012	A2.59	Not represented	Not represented - ecosystem are only represented at a generic level (e.g. 'ecosystems', 'landscape', 'habitat' etc.)	N/A. Human health not represented.	Not represented- no determinants of 'ecosystems' etc. shown.	N/A as there is no representation of human or ecosystem health. No other feedback mechanisms shown.	Not represented	Spatial dimension is the key feature of the diagram, depicting (on a continuum) local/micro through to global/macro- space, with examples of different types of natural or constructed space for each spatial level
Bowd et al	2012	A2.60	Not represented	Not represented- just the 'resource' and 'ecosystem services' at a generic level.	N/A. Human health not represented.	'Resource' is impacted on by 'resource users' and' public infrastructure providers'.	N/A as there is no representation of human health. However, each of the four components (the resource, the resource users, public infrastructure, and public infrastructure providers) interlink, through different assessment steps.	Not represented	Not explicitly represented, however a spatial dimension relating to resource users is recognised ('those that live amongst, adjacent to, upstream, downstream of the ecosystem services' and 'those that travel to use the ecosystem services')
Chan et al (a)	2012	A2.61	Not represented	Not explicitly represented, however 'categories of ecosystem services' are shown, including 'supporting services' consisting of 'processes (e.g. pollination), organisms, sites, habitats'.	N/A. Human health not represented.	Not represented - no determinants of ecosystem services shown.	N/A as there is no representation of human health. Only one-directional relationship from 'categories of ecosystem services' through to 'kinds of values'.	Not represented	Not represented
Chan et al (b)	2012	A2.62	Not represented	Not represented - just 'ecosystems services' at a generic level, and also 'socio-ecological context' which includes the 'biophysical'.	N/A. Human health not represented.	The 'benefits, ES and values' stage is determined by the 'socio-ecological context' stage which is determined by the 'decisions' stage.	N/A as there is no representation of human health. However, other than step 1 (consent), the remaining four steps are interlinked, both through a circular feedback mechanism and two-way interactions.	Not represented	Not represented
Comello et al	2012	A2.63	Not represented	Not explicitly represented, however 'assess ecosystem functions' is included.	N/A. Human health not represented.	The 'assess ecosystem functions' stage is influenced by the previous stage of 'determine lifecycle inventory', which is determined by 'decision analysis'.	N/A as there is no representation of human health. However, a circular feedback loop is shown between the different components of the valuation process.	Not represented	Not represented
Luck et al	2012	A2.64	Not represented	Not represented - only the concept of species 'traits' and 'environmental change'.	N/A. Human health not represented.	N/A Ecosystem health not represented and no determinants of 'traits' shown.	N/A as there is no representation of human or ecosystem health. Only one-directional step-by-step process.	Not represented	Not represented
Bastian et al	2013	A2.65	Represented in a minor way as 'well-being' under 'benefits, values'.	'Structure, components, processes, fluxes' are included within the 'properties' pillar. 'Potentials for land use' (including resilience) is also included.	Benefits, values (well-being)' are determined by ecosystem 'services', as well 'spatial aspects' (spatial scales, dimension, patterns) and 'temporal aspects' (time scale, driving forces, changes and scenarios). Also influenced by 'handling (stakeholders)' and 'valuation (socio-economic)'.	'Properties' (incl. structure, processes etc.) are determined by 'use, management, decision, participation, steering', as well 'spatial aspects' (spatial scales, dimension, patterns) and 'temporal aspects' (time scale, driving forces, changes and scenarios).	There is circular feedback mechanisms between the five pillars, thus linking 'properties' and 'potentials' with 'benefits, values (well-being)' (via 'services', 'users, beneficiaries' and 'use, management, decision, participation, steering.')	The temporal aspect (time scale, driving forces, changes and scenarios) is illustrated as linking to the five pillars of the system through a number of processes (basis, opportunities, utilization, valuation, handling).	The spatial aspect of assessment (spatial scales, dimension, patterns) links with each of the five pillars of the system.
Ernstson	2013	A2.66	Not represented	Not explicitly represented- however the generation of ecosystem services is a feature, and ecological dynamics are represented as nodes, interconnecting with each other through ecological functions and flows	N/A. Human health not represented.	No external determinants of the ecological nodes shown, although they interact with each other.	N/A as there is no representation of human health. However, different components interact with each other, e.g. ecological nodes in diagram A, and the 'social arena', 'actors' and 'artefacts' in diagram B.	Not represented	The spatial dimension is represented in two key ways- firstly, through presentation of a meso-scale (city-wide) diagram and a local-scale diagram. Secondly, the first diagram depicts the spatial connectivity of socioecological processes (reflecting the influence of different locations on one another)
Honrado et al	2013	A2.67	Not represented	Not represented - just the general term 'ecosystems' and 'ES identification'.	N/A. Human health not represented.	No determinants of 'ecosystems' shown.	N/A as there is no representation of human or ecosystem health. No interactions shown.	Not represented	Not represented
Kelble et al	2013	A2.68	Not represented	Represented under 'states', which refers to 'the condition of the physical, chemical, and biological attributes' of the environment.	N/A. Human health not represented.	'State' (includes ecosystem condition) is determined by 'pressures' (physical, chemical and biological mechanisms that are the proximal causes of change in the ecosystem), which is affected by 'drivers' (ultimate causes of change in the ecosystem... any combination of biophysical, human and institutional actions or processes). 'State' is also driven by 'responses'.	N/A as there is no representation of human health. However, there is a circular feedback mechanism between 'Ecosystem Services', 'State', 'Pressures' and 'Drivers', and there are two-way feedbacks between each of these components with 'Responses'.	Not represented	Not represented
Lavorel et al	2013	A2.69	Not represented	Not represented, although different species traits are depicted.	N/A. Human health not represented.	'Environmental driver' , which affect 'response traits', is included e.g. 'grassland management intensity'.	N/A as there is no representation of human health. Only one-directional step-by-step process shown.	Not represented	Not represented
Lopes & Videira	2013	A2.70	Not represented	Not represented - only the notion of how decisions affect ecosystem services.	N/A. Human health not represented.	N/A Ecosystem health not represented	N/A as there is no representation of human or ecosystem health. However, there is a circular feedback mechanism between the three decision-making stages, but also within components of the stages. Decisions being implemented can also lead back to new decisions being required.	Not explicitly represented, although there is reference to identifying 'long-term impacts on ecosystem services' within the 'deepening understanding' phase.	Not represented
Lopes & Videira	2013	A2.71	Not represented	Not represented - only 'values of ecosystem services'	N/A. Human health not represented.	'Values of ecosystem services' are determined by the four stages of the decision-making process, e.g. 'selecting the best solution' etc.	N/A as there is no representation of human health. However, there is a circular feedback mechanism between the four decision-making stages.	Not represented	Not represented
Maes et al	2013	A2.72	'Human well-being' is a key feature, and includes benefits such as 'health', 'safety', 'nutrition' etc. 'Health value' is also included as a type of 'value'.	Several different dimensions of 'biodiversity' are depicted within 'ecosystems' - 'ecological processes, functional traits, biophysical structures, genetic diversity, species richness, biotic interactions'. These lead to 'functions'.	'Ecosystem use and management' (derived from 'ecosystem service's), and 'other inputs', determine 'human well-being' benefits, including 'health'.	'Biodiversity' and 'ecosystems' are determined by 'drivers of change'.	There is a circular feedback mechanism between the 'socio-economic systems' which includes 'human well-being', and 'ecosystems', linked by 'ecosystem services' and 'drivers of change' (as well as 'values' and 'responses').	Represented as 'state- present and future'	Not represented
Morse et al	2013	A2.73	Not represented	Not explicitly represented, however 'Structure, patterns and processes' are included under 'patch mosaic' and 'patch level'.	N/A. Human health not represented.	Patch level/mosaic (which includes 'Structure, patterns and processes') is determined by 'actions/disturbance', which is also affected by the 'social systems' and 'actor's capacity'.	N/A as there is no representation of human health. However, there are other circular feedbacks throughout the framework, with 'feedbacks' depicted as a process in itself.	Time is captured weakly as a process of 'feedbacks/ time',	Not explicitly represented, however 'patches' are discrete spatial patterns within ecological systems- i.e. Patch mosaics have a spatial element.
Reed et al	2013	A2.74	Not represented	Not explicitly depicted. However ecosystem services are included and categorised, and include supporting services, which relate to ecosystem functioning.	N/A. Human health not represented.	'Stocks & flows of capital assets', which includes ecosystem services, are determined by the 'current context' (e.g. socio-economic, political and environmental, including tenure regime, agro-ecological zone, social norms etc.) and 'future change' (e.g. climate change, policy reform, socio-economic changes).	N/A as there is no representation of human health. There is a circular feedback mechanism between 'stocks & flows of capital assets' and 'vulnerability to socio-ecological change' via several processes, which also have further feedback mechanism between them.	The notion of 'future change (e.g. policy change, policy reform, socio-economic changes) in comparison to 'current context' (socio-economic, political and environmental, including tenure regime, agro-ecological zone, social norms) is depicted.	Not represented
Reis et al	2013	A2.75	'Health & well-being' is included under the 'Effect' component.	Not explicitly depicted. However ecosystem services are included and categorised, and include supporting services, which relate to ecosystem functioning.	'Determinants of health and well-being' are depicted in the 'Exposure/experience' component, broken down into 'security, material minima, social relations, and freedom & choice'. Additionally, 'Driver' and 'Pressure' determine 'State', which in turn determines 'Exposure/experience' which determines 'Effect (health & well-being)'.	Ecosystem services are determined by 'pressure'.	Mostly one-directional relationship shown, except between 'action' and the other components of the framework, which are two-way interactions, thus there is some feedback between 'state' (relating to ecosystems) and 'effect' (relating to health & well-being).	Not represented	Not represented
Reis et al	2013	A2.76	Not explicitly depicted, however 'determinants of health & well-being' are shown (within 'Experience' and 'Exposure').	Not explicitly depicted. However ecosystem services are included and categorised, and include supporting services, which relate to ecosystem functioning	'Determinants of health and well-being' are depicted in the 'Exposure/experience' component, broken down into 'security, material minima, social relations, and freedom & choice'. Additionally 'Pressure' affects 'State' (which includes ecosystem services) which in turn impacts upon the determinants of human health and well-being.	'Ecosystem services' are determined by 'pressure', but also are affected by 'determinants of health & well-being' including 'security, material minima, social relations, and freedom & choice'	Feedback loops shown between 'Ecosystem services' and 'determinants of health & well-being' (thus linking 'State' and 'Exposure/Experience')	Not represented	Not represented

Wang et al	2013	A2.77	Not represented	Not represented- only 'natural ecosystem' and the delivery of 'ecosystem services'	N/A. Human health not represented.	The 'natural ecosystem' is affected by 'human impacts' stemming from the social and economic system.	N/A as there is no representation of human health. However, there is a circular feedback mechanism between the 'natural ecosystem' and the 'social system'. Also feedback loops with the economic system.	Not represented	Not represented
Ahern et al	2014	A2.78	Not represented	Not represented - only 'ecosystem service goals'.	N/A. Human health not represented.	N/A Ecosystem health not represented and no determinants of 'ecosystem services goals' shown.	N/A as there is no representation of human or ecosystem health. However, feedback loops shown between the final step (apply findings) with earlier steps in the process (defining & prioritising ES goals, designing the experiment). Also two-way feedback between the different steps/actions and 'actors'.	Not represented	Not represented
Clark et al	2014	A2.79	Human health is the key 'outcome' in the framework, stemming from 'human well-being'.	Not represented - only 'biodiversity'.	Human health is affected by biodiversity via two pathways- direct effects (e.g. the regulation of the emergence and transmission of disease and pollution control) and indirect effects, through the loss of cultural goods and services.	N/A Ecosystem health not represented (and no determinants of biodiversity shown).	Not represented- only one-directional relationship from biodiversity to human health.	Not represented	Not represented.
Comello et al	2014	A2.80	Not represented	Not explicitly represented, however 'ecosystem function impacts' is included.	N/A. Human health not represented.	'Ecosystem function impacts' are determined by 'business/ firm-level activity'	N/A as there is no representation of human health. However, circular feedback mechanism shown between the different valuation components.	Not represented	Not represented
Duraiappah et al	2014	A2.81	'Human well-being' is a key component of the framework.	Not represented. However ecosystem services are included and categorised, although does not include supporting services (which most closely relate to ecosystem functioning). 'Nature's systems' - consisting of biodiversity and ecosystems- is also depicted.	'Human well-being' is determined by the 'productive base', which consists of human, productive and natural capital, the latter producing 'ecosystem services & other goods and services'.	'Nature's systems' are determined by 'human well-being'. Natural capital (providing ecosystem services) is determined by 'human well-being' and 'nature's systems' (with input from 'institutions and governance').	Circular feedback mechanism shown between the social and ecological system (human well-being and nature's systems). Also circular feedback between human well-being and the productive base (consisting of human, productive and natural capital).	Not represented	Spatial scale is depicted as a prominent feature, in three ways, relating to: (i) society (household, community, nation, region, globe); (ii) institutions & governance (community, national, regional, international) and (iii) ecosystems (resource system, landscape, bioregion, biome, planet).
Mastrangelo	2014	A2.82	Not represented	Not represented, just the concept of ES and their supply/production.	N/A. Human health not represented.	N/A Ecosystem health not represented (and no determinants of ES shown)	N/A as there is no representation of human or ecosystem health. Mostly one-directional relationships shown, however two-way feedback illustrated relating to assessing socio-ecological attributes and production functions & models.	Not represented	Only weakly represented- with the question of 'at which scale do socio-ecological attributes operate?'
Schröter et al	2014	A2.83	Not represented	Partially represented as 'ecosystem asset', which includes 'extent' and 'properties (condition)'.	N/A. Human health not represented.	'Ecosystem assets' (including 'properties (condition)') is determined by 'human inputs (ecosystem management)'.	N/A as there is no representation of human health. However there is a circular feedback loop between 'ecosystem assets' , 'ecosystem services' and 'society & economy' via 'human inputs (ecosystem management)'	Time' is mentioned as a factor relating to 'use patterns' of ES.	Spatial models are depicted as part of the input into ES measurement.
Serna-Chavez et al	2014	A2.84	Not represented	Not represented - only the idea of ecosystem service provisioning, flow and benefiting areas.	N/A. Human health not represented.	N/A Ecosystem health not represented, and no determinants of ecosystem service provisioning areas shown.	No feedbacks shown.	Not represented	Spatial dimension is the key feature, showing the different spatial arrangement of areas providing ES, the spatial extent of the flow of ES from that area, and the possible spatial relationship with ES benefiting areas.
